# Comparative Antioxidant Evaluation and GC-MS Identification of Bioactive Constituents in *Litsea cubeba* (Lour.) Pers. Fractions

**DOI:** 10.3390/molecules31091506

**Published:** 2026-04-30

**Authors:** Mengyue Wei, Zihan Yu, Wenyi Fang, Yunbin Zhang, Xiaofei Zhou

**Affiliations:** 1 Institute of Biomaterials and Biomedicine, School of Food and Pharmacy, Shanghai Zhongqiao Vocational and Technical University, Shanghai 201514, China; weimengyue8@163.com (M.W.); yzh963769256@163.com (Z.Y.); 17746086923@163.com (W.F.); 2 Shanghai Urban Construction Vocational College, School of Food and Tourism, Shanghai 201514, China; zhangyunbin@succ.edu.cn

**Keywords:** *Litsea cubeba* (Lour.) Pers., citral, antioxidant activity, solvent fractionation, GC-MS analysis

## Abstract

In this study, five solvent fractions from *Litsea cubeba* (Lour.) Pers. fruit were extracted and investigated for their antioxidant profiles. Results showed that the petroleum ether fraction (PEF) and n-butanol fraction (NBF) exhibited prominent free radical scavenging capacities in DPPH, ABTS, and hydroxyl radical assays. Gas chromatography–mass spectrometry (GC-MS) identified citral as the dominant bioactive component in both active fractions. Further mechanism analysis demonstrated that citral exerted potent antioxidant effects via dual pathways: direct free radical scavenging and transition metal ion chelation. These findings not only elucidate the material basis and molecular mechanism underlying the antioxidant activity of *L. cubeba* but also provide a scientific rationale for the high-value utilization of citral-rich fractions in functional foods, cosmetics, and healthcare products.

## 1. Introduction

Oxidative stress, caused by the imbalance between reactive oxygen species (ROS) production and endogenous antioxidant defense systems, is widely recognized as a key pathological factor in various chronic diseases, including cardiovascular disorders, neurodegenerative diseases, diabetes, and cancer [1,2]. Excessive ROS can damage lipids, proteins, and DNA, leading to cellular dysfunction and tissue damage. In this context, natural antioxidants derived from edible plants have attracted increasing attention over the past decades, as they are considered safer, more biocompatible, and more sustainable than synthetic antioxidants for food preservation, cosmetics, and pharmaceutical applications [3].

Traditionally, polyphenols and flavonoids have been regarded as the primary contributors to the antioxidant activity of plant extracts due to their excellent electron-donating, hydrogen-donating, and radical-scavenging capacities [4,5,6]. Numerous studies have confirmed a significant positive correlation between total phenolic content and antioxidant activity in various plant extracts [7,8]. However, increasing evidence has revealed that the antioxidant system of plants is far more complex than previously thought. Different in vitro antioxidant assays reflect distinct reaction mechanisms: DPPH and ABTS assays evaluate radical scavenging capacity, while FRAP measures the ferric ion reducing power [9]. Moreover, nonphenolic components such as terpenoids, fatty acids, and alkaloids can also play critical roles in specific radical-scavenging processes, leading to the decoupling of total phenolic/flavonoid content from antioxidant activity [10,11]. This phenomenon has been observed in many medicinal plants, but its underlying mechanism in *Litsea cubeba* (Lour.) Pers. remains poorly understood.

*L. cubeba*, a member of the Lauraceae family, is an important spice and medicinal plant widely distributed in southern China, Southeast Asia, and India [12,13]. In China, it is especially abundant in Guizhou, Yunnan, and Sichuan provinces, where its fruits have long been used as a traditional ethnic medicine for treating indigestion, colds, rheumatic pain, and abdominal pain [14]. Previous phytochemical and pharmacological studies have demonstrated that *L. cubeba* possesses a wide range of biological activities, including antioxidant, antimicrobial, anti-inflammatory, hypoglycemic, and anticancer effects [12,15,16]. Most of these studies, however, have focused primarily on its essential oil, which is rich in volatile components such as citral, limonene, and linalool [17,18]. Non-volatile components, as well as the systematic comparison of different polar solvent fractions from its fruits, remain largely unexplored [19]. A few previous attempts have been made to investigate the solvent fractions of *L. cubeba*, but most of them were conducted on leaves or branches rather than fruits and failed to clarify the underlying mechanism of the activity–component discrepancy [20]. For example, Wang et al. compared the non-volatile components and antioxidant activity between branches and leaves but did not analyze the polar fractionation profile [21]. According to their investigation, both branches and leaves of *L. cubeba* possess considerable in vitro antioxidant capacity, as evidenced by DPPH, ABTS, and reducing power assays. These tissues are rich in polyphenols, flavonoids, and terpenoids, which are considered the main contributors to their free radical-scavenging and antioxidant effects. These findings further demonstrate that different tissues of *L. cubeba* have notable antioxidant potential and contain abundant bioactive substances, providing a useful reference for understanding the antioxidant activity of this species.

Furthermore, most previous studies on *L. cubeba* have focused on essential oil or single extraction procedures, while systematic solvent fractionation combined with bioassay-guided isolation remains largely unexplored. Solvent polarity strongly influences phytochemical extraction efficiency and antioxidant activity of the obtained fractions [22,23]. Accordingly, systematic evaluation of polar fractions is critical to clarify the complex antioxidant system of *L. cubeba*. To address these gaps, the objective of this study was to screen antioxidant-active fractions among different solvent extracts of *L. cubeba* fruits, characterize their major chemical constituents, and identify the key antioxidant component. We systematically evaluated the antioxidant profiles of five solvent fractions, subjected the two most active fractions to GC-MS analysis, and successfully elucidated citral as the primary contributor to the antioxidant activity of *L. cubeba* fruits.

## 2. Results and Discussion

### 2.1. Yield, Total Phenolic, and Flavonoid Contents of Different Solvent Fractions

The yield and content characteristics of different solvent fractions from *L. cubeba* are summarized in Table 1. The extraction yield of the crude ethanol extract (LCE) reached 18.7%, which was significantly higher than that of the solvent fractions, indicating that ethanol effectively extracted the majority of phytochemicals. Among the fractions, the aqueous fraction (AQF, 7.5%) and n-butanol fraction (NBF, 5.3%) exhibited relatively higher yields, while the petroleum ether fraction (PEF, 3.6%) and ethyl acetate fraction (EAF, 3.8%) showed lower yields, consistent with the polarity distribution of plant secondary metabolites.

**Figure 1:**
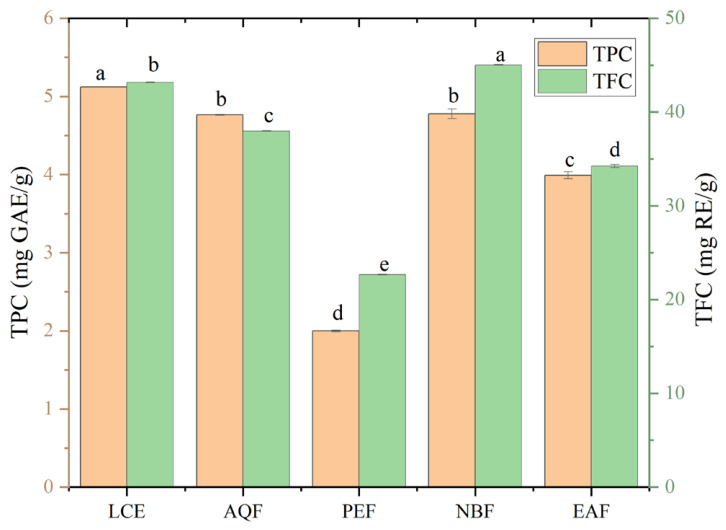
Total phenolic content (TPC) and total flavonoid content (TFC) of different solvent fractions from *L. cubeba*. Different lowercase letters above the bars indicate significant differences among different fractions (*p* < 0.05, Tukey’s multiple comparison test). LCE: crude ethanol extract; PEF: petroleum ether fraction; EAF: ethyl acetate fraction; NBF: n-butanol fraction; AQF: aqueous fraction.

The total phenolic content (TPC) and total flavonoid content (TFC) varied significantly across different fractions (Figure 1). TFC was determined with a rutin standard (y = 2.0939x + 0.0183, R^2^ = 0.9991) and expressed as rutin equivalents (REs). TPC was quantified with a gallic acid standard (y = 2.808x + 0.0012, R^2^ = 0.9994) and expressed as gallic acid equivalents (GAEs). It should be noted that TFC was higher than TPC in some fractions, which appears inconsistent with the fact that flavonoids are a subclass of phenolic compounds. This discrepancy is mainly attributed to the limitations of the quantitative methods. The Folin–Ciocalteu assay used for TPC has low specificity and may underestimate phenolics in the presence of lipophilic components. In contrast, the aluminum-based colorimetric method for TFC tends to overestimate flavonoid contents due to interference from other non-flavonoid reducing substances. Therefore, TPC and TFC values are not directly comparable, and the observed difference does not contradict the chemical classification. For TPC, LCE displayed the highest level (5.12 mg GAE/g), followed by NBF (4.78 mg GAE/g) and AQF (4.76 mg GAE/g), while PEF had the lowest TPC (2.00 mg GAE/g). Regarding TFC, NBF exhibited the maximum value (45.02 mg RE/g), which was higher than other fractions. LCE and AQF showed moderate TFC levels, whereas PEF and EAF had the lowest values, with PEF recorded as the minimum (22.66 mg RE/g). Notably, PEF, despite having the lowest TPC and TFC among all fractions, demonstrated distinct content distribution characteristics. The extraction efficiency of different solvents is closely related to solvent polarity and the molecular structure of bioactive compounds. According to the like-dissolves-like principle, low-polarity PEF mainly extracts lipophilic non-polar substances, leading to the lowest TPC and TFC among all fractions. In contrast, moderately polar NBF exhibits a favorable enrichment ability for phenolic and flavonoid components, thus possessing relatively high TPC and TFC values. This difference is mainly determined by the polarity matching between solvents and active ingredients. Collectively, these results revealed that solvent polarity significantly influenced the extraction efficiency of phenolics and flavonoids, with NBF and LCE identified as the main fractions rich in these bioactive components.

### 2.2. Antioxidant Activities and Correlation Analysis of Different Fractions from L. cubeba

**Figure 2:**
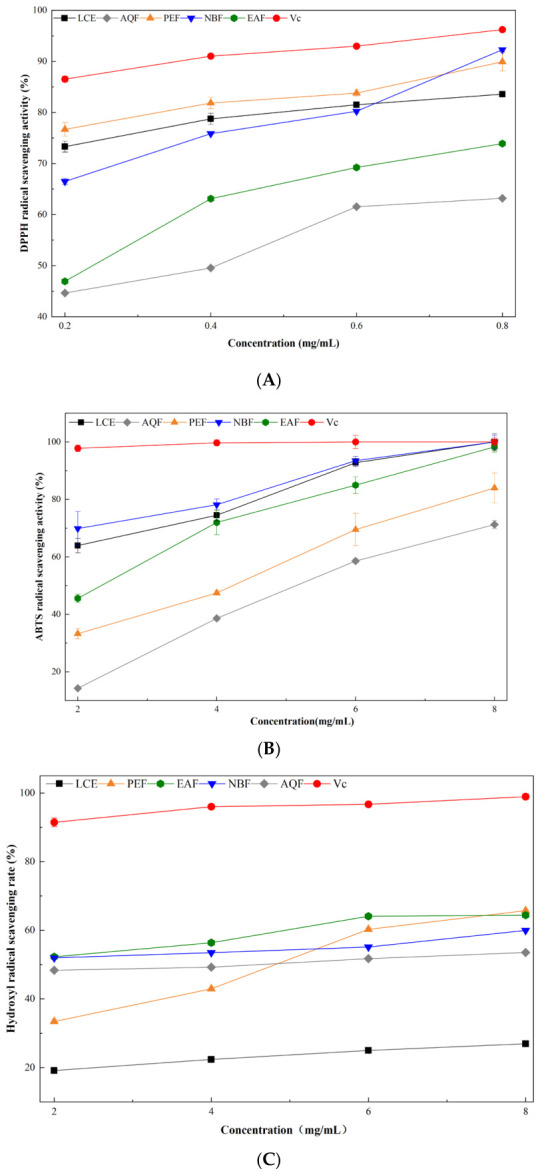

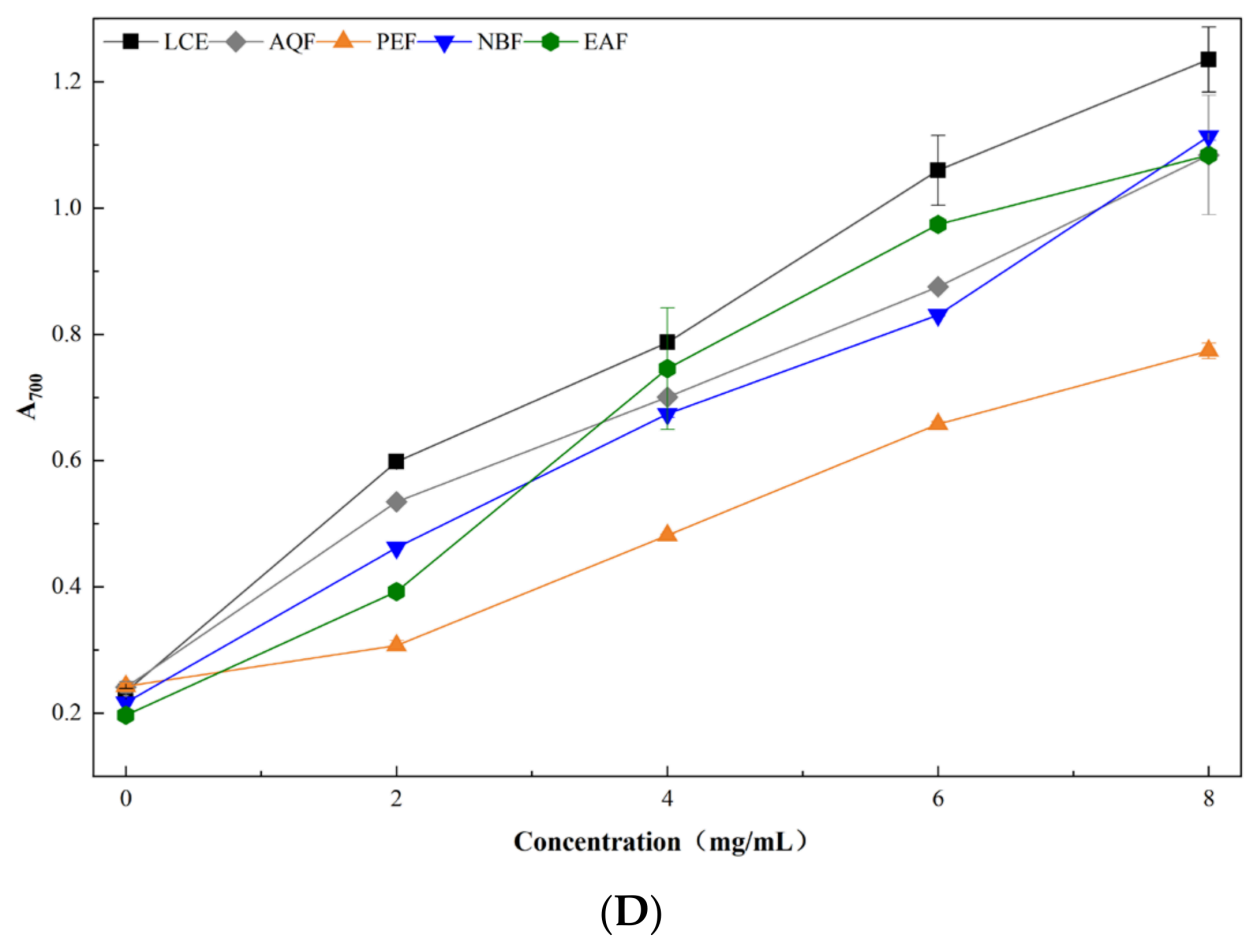
In vitro antioxidant activities of different fractions from *L. cubeba* ethanol extract and its fractions: (**A**) DPPH radical scavenging activity; (**B**) ABTS radical scavenging activity; (**C**) hydroxyl radical scavenging activity; (**D**) FRAP.

As illustrated in Figure 2A and Table S1, all *L. cubeba* extracts exhibited significant concentration-dependent DPPH radical scavenging activity across the tested concentration range (0.2–0.8 mg/mL). At the maximum concentration of 0.8 mg/mL, NBF and PEF showed the strongest scavenging capacity, with rates of 92.3% and 89.9%, respectively, which were only slightly lower than that of the positive control VC (96.2%) and markedly higher than the crude extract LCE (83.6%). AQF displayed the weakest activity, with a scavenging rate of 63.2% at 0.8 mg/mL. These results indicate that the potent DPPH-scavenging components of *L. cubeba* are predominantly enriched in the moderately polar NBF and non-polar PEF fractions.

Consistent with the DPPH assay, all *L. cubeba* fractions exhibited significant concentration-dependent ABTS radical scavenging activity across the range of 2–8 mg/mL (Figure 2B and Table S1). At the maximum concentration of 8 mg/mL, NBF and VC displayed the highest activity. PEF and EAF also exhibited strong antioxidant capacity, with activities reaching 83.9% and 98.3% at 8 mg/mL, respectively. In contrast, AQF showed the weakest ABTS scavenging activity, with a rate of only 71.3% at the highest concentration. These findings demonstrate that the potent ABTS-scavenging components of *L. cubeba* are primarily enriched in the non-polar (PEF) and moderately polar (NBF, EAF) fractions, while the aqueous phase (AQF) contains relatively fewer active compounds.

All *L. cubeba* fractions exhibited a concentration-dependent increase in hydroxyl radical scavenging activity across the tested concentration range of 2–8 mg/mL (Figure 2C and Table S1). At the maximum concentration of 8 mg/mL, VC showed the strongest •OH scavenging capacity, with a scavenging rate approaching 100%. Among the fractions, EAF and PEF displayed the highest activity, with scavenging rates of 64.4% and 65.8%, respectively, followed by NBF (60.0%), AQF (53.5%), and LCE (27.0%). These results indicate that EAF and PEF possess potent •OH scavenging properties, while LCE exhibits relatively weaker activity against hydroxyl radicals.

The FRAP assay was further employed to assess the total antioxidant capacity of the *L. cubeba* fractions. As shown in Figure 2D and Table S1, all tested fractions exhibited a concentration-dependent increase in A700 values, indicating a positive correlation between sample concentration and reducing ability. At the highest concentration of 8 mg/mL, LCE displayed the strongest reducing power with an A700 value of 1.24, followed by NBF (1.11), AQF (1.08), EAF (1.08), and PEF (0.77). These results collectively suggest that the crude extract (LCE) possesses the highest total reductive capacity, while NBF, AQF, and EAF also exhibit strong reducing power. In contrast, PEF shows relatively weaker ferric reducing ability, which is consistent with its lower content of polar reducing compounds.

As shown in Table 2, PEF exhibited the strongest DPPH radical scavenging activity (IC_50_ = 0.270 mg/mL). NBF showed optimal scavenging capacity against ABTS and hydroxyl radicals, with IC_50_ values of 1.238 mg/mL and 1.622 mg/mL, respectively. The positive control VC displayed potent activity in both ABTS and •OH assays, with IC_50_ values of 1.161 mg/mL and 0.2627 mg/mL, respectively. Combined with total polyphenol and flavonoid contents, the high polyphenol content in NBF contributes to its strong ABTS and •OH scavenging activity. Notably, PEF shows the strongest DPPH scavenging capacity despite the lowest polyphenol and flavonoid contents, indicating the presence of potent nonpolyphenolic antioxidants such as terpenoids. This discrepancy reveals the complex and diverse antioxidant system of *L. cubeba*, where synergistic effects among different fractions may enhance the overall antioxidant activity.

To further explore the potential relationship between total flavonoid and total polyphenol contents and antioxidant activities in different fractions of the ethanol extract of *L. cubeba*, correlation analysis was conducted using SPSS 27.0 with the Pearson correlation coefficient. TFC, TPC, and four in vitro antioxidant indicators (IC_50_ values for DPPH, ABTS, and hydroxyl radical scavenging, as well as FRAP values) were analyzed, and the results are presented in Table 3.

Both TPC and TFC showed highly significant positive correlations with FRAP (r = 0.962 and 0.887, respectively, *p* < 0.01), indicating that polyphenols and flavonoids are the main contributors to the ferric reducing activity. Meanwhile, TPC was significantly negatively correlated with the ABTS IC_50_ value (r = −0.669, *p* < 0.05). Notably, PEF had the lowest TPC and TFC but exhibited the strongest DPPH radical scavenging capacity. This inconsistency implies the existence of potent nonphenolic and lipophilic active components in PEF. Therefore, PEF and NBF were selected for further chromatographic separation to characterize their respective bioactive constituents: NBF exhibited the optimal scavenging capacity against ABTS and hydroxyl radicals, which was closely related to its high polyphenol content, while PEF showed the strongest DPPH scavenging activity despite low polyphenol and flavonoid contents, suggesting the presence of unique nonphenolic antioxidant components; together, these two fractions represent the most potent and functionally distinct antioxidant fractions, making them ideal for further isolation and identification of bioactive compounds.

### 2.3. Antioxidant Active Components of NBF and PEF

PEF and NBF were further separated into eight and six subfractions via silica gel column chromatography, respectively. After secondary activity verification, the most active subfractions (PEF-Fr and NBF-Fr) were subjected to GC-MS analysis to identify their main chemical components. The relative ABTS scavenging activities of the six NBF subfractions were 100.00 ± 0.44%, 93.87 ± 0.09%, 85.47 ± 0.22%, 79.03 ± 0.04%, 78.36 ± 0.05%, and 83.57 ± 0.46%, respectively. The relative FRAP values of the eight PEF subfractions were 100.00 ± 0.00%, 83.23 ± 0.01%, 78.22 ± 0.01%, 75.23 ± 0.01%, 68.95 ± 0.01%, 62.00 ± 0.01%, 57.29 ± 0.01%, and 52.88 ± 0.01%, respectively. Accordingly, the subfractions with the highest antioxidant activity from NBF and PEF were selected, designated as NBF-Fr and PEF-Fr, respectively, for subsequent GC-MS analysis to identify their bioactive components.

As presented in Table 4 and Table 5, the chemical compositions of the active fractions from PEF-Fr and NBF-Fr exhibited both similarities and differences. The PEF-Fr fraction was dominated by terpenoids (Supplementary File S1), among which citral (including cis- and trans-isomers) accounted for the highest content, with a total relative content of 50.76%, representing the characteristic component of this fraction. In addition, alkanes (heptacosane and substituted octadecane), alcohols (linalool), and a small amount of ketones (1-(2-hydroxyphenyl)-3-phenyl-1,3-propanedione) were also identified. For the NBF-Fr fraction (Supplementary File S2), the total content of citral reached 46.57%, which was similar to that in PEF-Fr. Meanwhile, this fraction was rich in n-decanoic acid (18.44%) and n-butanol (13.51%), indicating that the NBF active fraction was a composite system mainly composed of terpenoid aldehydes, fatty acids, and alcohols.

**Figure 3:**
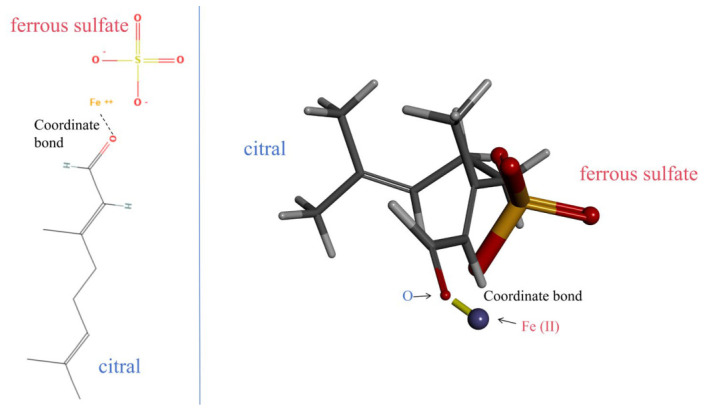
Chelation interaction between citral and ferrous sulfate. The optimal binding pose showed a Grid_Score of −5.29 kcal/mol with a van der Waals energy of −4.60 kcal/mol and an electrostatic energy of −0.70 kcal/mol. The stable chelation was verified by zero repulsive energy and a stable binding conformation.

GC-MS identification demonstrated that citral (cis- and trans-isomers) was the most abundant component in both the PEF-Fr and NBF-Fr fractions of *L. cubeba*, with total relative contents of 50.76% and 46.57%, respectively. As the dominant constituent shared by both active fractions, citral served as the key chemical basis for the strong antioxidant activities observed in both extracts. To date, although numerous studies have reported the antioxidant potential of *L. cubeba* essential oil and extracts, most have focused only on the overall activity evaluation and preliminary component analysis, while systematic investigations into the structure–activity relationship of its core active compounds and the corresponding free radical scavenging mechanisms remain insufficient [24]. As the dominant and common ingredient in the two most active fractions, citral was considered the key contributor to the antioxidant activity of *L. cubeba* fruit extracts. The α, β-unsaturated aldehyde group in the citral molecule is widely recognized as the key functional moiety responsible for its outstanding antioxidant capacity [25,26]. Citral efficiently neutralizes various ROS by donating labile hydrogen atoms and electrons, thereby stabilizing DPPH, ABTS, and other stable free radicals and terminating radical chain reactions [27]. Compared with ordinary aldehyde compounds, the conjugated double bond system in citral promotes the delocalization of unpaired electrons, which significantly enhances its free radical scavenging efficiency and broadens its antioxidant spectrum [28]. Recent studies have confirmed that citral, a monoterpene aldehyde, exhibits strong antioxidant, anti-inflammatory, and antimicrobial activities [28]. Citral can scavenge ROS and activate the Nrf2 antioxidant pathway, a key cellular defense system against oxidative stress. Activation of Nrf2 promotes the expression of downstream antioxidant proteins, thereby enhancing cellular resistance to oxidative damage [29]. This further explains the strong antioxidant activity of citral-rich *L. cubeba* fractions demonstrated in our present study. Nevertheless, the specific contribution of citral to the antioxidant activity of different polar solvent fractions and its role in explaining the activity–component decoupling phenomenon have not been systematically elucidated in *L. cubeba*. Such structural advantages explain why citral-rich fractions from *L. cubeba* exhibited excellent performance in multiple antioxidant evaluation systems in this study. In addition to direct free radical scavenging, the aldehyde group in citral also possesses strong metal ion chelating potential [30]. Molecular visualization further revealed that the aldehyde oxygen atom in citral could coordinate with Fe^2+^ ions to form a stable chelate complex, thereby effectively sequestering iron ions and inhibiting Fenton reaction-induced hydroxyl radical production (Figure 3).

As a valuable aromatic and medicinal plant widely used in traditional medicine and industrial production, *L. cubeba* has received extensive attention for its rich volatile components and remarkable biological activities [12,17,31]; however, most existing studies only focus on its essential oil, ignoring the active distribution and mechanism of key components in polar fractions such as n-butanol, and lack systematic explanation of its structure-activity relationship and action mechanism [32,33].

Notably, most previous studies focused only on essential oils, leaves, or branches, while a systematic comparison of fruit-derived polar fractions remains insufficient [34,35,36]. In comparison with previous phytochemical investigations on *L. cubeba* leaves and stems, vegetative tissues mainly accumulate simple phenolic acids and monoterpenes, while fruit fractions are richer in flavonoid derivatives and polyphenolic metabolites, showing obvious tissue-specific chemical distribution characteristics [21]. Our work complements these reports by revealing that both non-polar PEF and medium-polar NBF from *L. cubeba* fruits possess prominent antioxidant activity, even with distinct phenolic and flavonoid levels. Furthermore, the distinct antioxidant profiles observed in different solvent fractions highlight the significant influence of extraction solvent polarity on the enrichment and distribution of bioactive substances in *L. cubeba* fruits [37]. The present comparative investigation provides an overview of the antioxidant potential of each polarity-based fraction, which can serve as a rational reference for targeted extraction and efficient utilization of antioxidant components from this plant. These results also demonstrate that *L. cubeba* fruits contain a wide variety of antioxidant components with diverse polarities, rather than only volatile constituents commonly reported in essential oil studies [38,39].

The findings of this study confirm that citral is the core antioxidant component of different polar parts of *L. cubeba*, fill the research gap in the molecular mechanism of antioxidant activity of *L. cubeba*, and also lay a theoretical foundation for the deep processing and high-value utilization of *L. cubeba* resources in natural antioxidants, functional foods, cosmetics, and plant health products, which is of great significance for promoting the sustainable development and industrial chain extension of this important economic plant.

## 3. Materials and Methods

### 3.1. Materials and Reagents

*L. cubeba* fruits were collected from Guizhou Province, China. *L. cubeba* fruits were collected from mid-August to early September. At this collection stage, the fruits presented a purple-black appearance and reached full maturity with consistent ripeness. Reagents, namely absolute ethanol, petroleum ether, ethyl acetate, n-butanol, sodium carbonate, aluminum nitrate, sodium nitrite, potassium persulfate, ferrous sulfate, salicylic acid, hydrogen peroxide (30%), potassium ferricyanide, ferric chloride, and trichloroacetic acid, were of analytical grade and purchased from Sinopharm Chemical Reagent Co., Ltd. (Shanghai, China). Gallic acid (purity ≥ 98%) and rutin (purity ≥ 98%) standards were obtained from Shanghai Yuanye Bio-Technology Co., Ltd. (Shanghai, China). Folin–Ciocalteu reagent was supplied by Beijing Solarbio Science & Technology Co., Ltd. (Beijing, China). DPPH (purity ≥ 97%) and ABTS (purity ≥ 98%) were purchased from Shanghai Titan Scientific Co., Ltd. (Shanghai, China). Ultrapure water was used throughout the experiments.

### 3.2. Preparation of L. cubeba Ethanol Extract (LCE)

Extraction was performed using an ultrasonic-assisted heating reflux method. Dried *L. cubeba* fruits were crushed using an 800A multi-function grinder (Yongkang Red Sun Electromechanical Co., Ltd., Yongkang, China) and passed through a 40-mesh sieve. The powder (20.0 g) was mixed with 254 mL of absolute ethanol (solid–liquid ratio 1:12.7 g/mL) in a 1000 mL round-bottom flask and macerated at room temperature for 1 h. The mixture was then subjected to ultrasonic-assisted extraction in a water bath at 40 °C for 60 min. The extract was cooled and centrifuged at 6500 rpm at 4 °C for 15 min. The residue was washed with ethanol and centrifuged again, and the supernatants were combined and vacuum-filtered three times. The filtrate was concentrated using a rotary evaporator at 55 °C under reduced pressure, yielding a dark brown, viscous LCE. The extract was dried under vacuum to constant weight, sealed, and stored in the dark at 4 °C.

### 3.3. Fractionation of LCE

For further fractionation, an equal volume gradient solvent separation was carried out based on different solvent polarities. Briefly, 3 mL of LCE was mixed with 30 mL of petroleum ether and ultrasonically treated at 45 °C for 40 min. After incubation, the mixture was concentrated under reduced pressure at 40 °C to obtain PEF. Under consistent operational procedures, the crude extract was separately processed with ethyl acetate, n-butanol, and distilled water. The ethyl acetate fraction was prepared via ultrasonic treatment at 45 °C for 40 min and concentrated at 50 °C. Both n-butanol and aqueous samples were ultrasonically incubated at 96 °C for 40 min and then concentrated under reduced pressure at 95 °C and 102 °C, respectively, to remove residual solvents. Finally, five polar fractions, namely LCE, PEF, EAF, NBF, and AQF, were obtained. All samples were sealed and preserved in a refrigerator under dark conditions for subsequent experiments.

### 3.4. Determination of Total Phenolic and Flavonoid Contents

Total phenolic content (TPC) was determined using the Folin–Ciocalteu method. Sample solutions (10 µL) were mixed with 50 µL of Folin–Ciocalteu reagent and incubated for 2 min. Subsequently, 50 µL of 7.5% Na_2_CO_3_ and 90 µL of distilled water were added, mixed, and incubated in the dark at room temperature. Absorbance was measured at 765 nm. TPC was calculated as mg gallic acid equivalents per gram of extract (mg GAE/g).

Total flavonoid content (TFC) was measured using the aluminum nitrate colorimetric method. Standard or sample solutions (80 µL) were mixed with 20 µL of NaNO_2_ reagent and incubated at 25 °C for 6 min. Next, 20 µL of Al(NO_3_)_3_ reagent was added and incubated for 6 min, followed by the addition of NaOH reagent. After incubation at 25 °C for 15 min, absorbance was measured at 510 nm. All absorbance measurements in this study were recorded using an Agilent Cary 60 UV-Vis spectrophotometer (Agilent Technologies, Santa Clara, CA, USA). TFC was calculated as mg rutin equivalents per gram of extract (mg RE/g).

The target compound contents (TPC and TFC) were calculated using the following equation:

C = (c × V × d)/m

where C is the phenolic or flavonoid content expressed as standard equivalents (mg/g), c is the concentration derived from the standard calibration curve (mg/mL), V is the total volume of the sample extract (mL), d is the dilution factor, and m is the mass of the extracted sample (g).

### 3.5. In Vitro Antioxidant Activity Assays

The antioxidant capacities of the extracts were evaluated using DPPH, ABTS, hydroxyl radical (•OH) scavenging assays, and the Ferric Reducing Antioxidant Power (FRAP) assay. Calibration curves were established using vitamin C standard solutions at the following concentration ranges: 0.2–0.8 mg/mL for DPPH; 2–8 mg/mL for ABTS; and 2–8 mg/mL for hydroxyl radical. For the radical scavenging assays, the scavenging rate (%) was determined using the following equation with background correction:

Scavenging rate (%) = [1 − (A_1_ − A_2_)/A_0_] × 100

where

A_0_: absorbance of the blank control (radical solution + solvent);

A_1_: absorbance of the sample (radical solution + extract solution);

A_2_: absorbance of the sample background (extract solution + solvent without radicals).

The IC_50_ value, representing the concentration of the extract required to achieve 50% scavenging activity, was calculated via non-linear regression analysis of the dose-response curves using OriginPro 2026 software.

#### 3.5.1. DPPH Radical Scavenging Assay

Sample solutions (800 µL, 0.2–0.8%) were mixed with 20 mL of 0.1 mmol/L DPPH-ethanol solution and incubated in the dark for 30 min. The absorbance was measured at 517 nm. Vitamin C (VC) served as the positive control.

#### 3.5.2. ABTS Radical Scavenging Assay

The ABTS working solution was prepared by reacting 7.0 mmol/L ABTS with 2.45 mmol/L potassium persulfate in the dark for 12–16 h. The solution was then diluted with 10 mmol/L PBS (pH 7.4) to an absorbance of 0.70 ± 0.02 at 734 nm. Sample solutions (0.4 mL) were mixed with 15.6 mL of ABTS working solution, incubated for 10 min, and measured at 734 nm.

#### 3.5.3. Hydroxyl Radical (•OH) Scavenging Assay

The reaction mixture contained 5 mL of sample solution, 1.5 mL of 8 mmol/L FeSO_4_, 5 mL of 3 mmol/L salicylic acid–ethanol, and 1.25 mL of 20 mmol/L H_2_O_2_. After incubation at 37 °C for 30 min, the reaction was terminated with 2.25 mL of distilled water. Following centrifugation at 3000 rpm for 10 min, the absorbance was measured at 510 nm.

#### 3.5.4. Ferric Reducing Antioxidant Power (FRAP) Assay

Sample solutions (1 mL) were mixed with 2.5 mL of 1% K_4_ [Fe(CN)_6_] and 2.5 mL of PBS (0.2 mol/L, pH 6.6) and incubated at 50 °C for 20 min. After adding 2.5 mL of 10% trichloroacetic acid and centrifuging, 1.7 mL of 0.1% FeCl_3_ was added to the supernatant. The absorbance was measured at 700 nm after 10 min. Results are expressed as absorbance at 700 nm. Vitamin C was excluded due to out-of-range absorbance (>2.0).

### 3.6. Bioassay-Guided Column Chromatography and Secondary Screening

Based on the initial antioxidant screening, thin-layer chromatography (TLC) was used to optimize the solvent system for the active PEF and NBF fractions. Petroleum ether/ethyl acetate/acetic acid (9:2:0.2, *v*/*v*/*v*) was selected as the optimal eluent, providing clear separation with no tailing. Normal-phase silica gel column chromatography was performed on a wet-packed column (100–200 mesh). Samples were mixed with silica gel (1:1.5, *w*/*w*) and dry-loaded onto the column. Isocratic elution was applied at 2–3 mL/min, and eluates were collected and pooled according to TLC profiles to obtain primary subfractions. These subfractions were further subjected to repeated silica gel column chromatography, yielding 6 purified fractions from NBF and 8 purified fractions from PEF.

For secondary activity screening, ABTS scavenging was used for the NBF fractions and FRAP for the PEF fractions, targeting those with significantly enhanced activity. After dose-effect verification, the most active fractions were chosen for further identification by GC-MS.

### 3.7. Gas Chromatography–Mass Spectrometry (GC-MS) Analysis

The chemical composition of the peak active subfractions was analyzed using an Agilent GC-MS system equipped with an electron ionization (EI) source. Separation was performed on an HP-5MS capillary column (30 m × 0.25 mm i.d., 0.25 μm film thickness). Helium was used as the carrier gas at a constant flow rate of 1.0 mL/min. The injection volume was 1.0 μL with a split ratio of 10:1. The injector temperature was maintained at 250 °C. The oven temperature program was initiated at 60 °C (held for 2 min), ramped to 150 °C at 5 °C/min, then increased to 280 °C at 10 °C/min and held for 5 min. The mass spectrometer was operated in EI mode at 70 eV. Mass spectra were acquired in full scan mode with a mass range of *m*/*z* 35–550. Compound identification was conducted by comparing the obtained mass spectra with the NIST 17 mass spectral library. Only compounds with a match score greater than 600 were considered acceptable. The relative percentage of each compound was calculated based on the peak area normalization method. In addition, only compounds with relative content ≥0.5% were selected and listed in Table 4 and Table 5. These criteria ensure the accuracy and reliability of compound identification.

### 3.8. Molecular Simulation

The 3D chemical structures of citral (PubChem CID: 638011) and ferrous sulfate (PubChem CID: 24393) were retrieved from the PubChem database. A molecular docking simulation was performed using the DOCK 6.9 program (UCSF, San Francisco, CA, USA). The total binding affinity was evaluated by Grid_Score, which was further decomposed into van der Waals energy (Grid_vdw_energy) and electrostatic energy (Grid_es_energy). Molecular visualization, hydrogen addition, and interaction pattern analysis were carried out using BIOVIA Discovery Studio Visualizer 2021 (Dassault Systèmes, San Diego, CA, USA).

### 3.9. Statistical Analysis

Data are expressed as mean ± standard deviation (SD). Statistical analysis and Pearson correlation coefficients were calculated using SPSS 27.0 software. *p* < 0.05 was considered statistically significant.

## 4. Conclusions

This study revealed that both PEF and NBF from *L. cubeba* fruits possessed potent antioxidant activity. GC-MS analysis identified citral as the predominant component in both active subfractions (PEF-Fr and NBF-Fr), which exerted strong antioxidant effects through direct radical scavenging and metal ion chelation. These results confirm citral as the core lipophilic antioxidant responsible for the activity–component decoupling phenomenon, clarify its structure–activity mechanism, and provide a scientific basis for the high-value utilization of *L. cubeba* in functional foods, cosmetics, and health products.

## Figures and Tables

**Table 1:** Extraction yields of different solvent fractions from *L. cubeba*.

Sample	Extraction Yield (%)
Crude ethanol extract (LCE)	18.7
n-Butanol fraction (NBF)	5.3
Aqueous fraction (AQF)	7.5
Ethyl acetate fraction (EAF)	3.8
Petroleum ether fraction (PEF)	3.6

**Table 2:** IC_50_ values of LCE and its fractions.

Items	Free Radical	VC	LCE	PEF	EAF	NBF	AQF
IC_50_ (mg/mL)	DPPH	0.200	0.273	0.270	1.015	2.255	2.905
	ABTS	1.161	1.482	3.543	2.257	1.238	5.040
	•OH	0.2627	10.000	4.362	1.689	1.622	2.749

**Table 3:** Pearson correlation coefficients between TFC, TPC, and antioxidant activities.

Item	TFC	TPC	DPPH	ABTS	•OH	FRAP
TFC	1					
TPC	0.941 *	1				
DPPH	0.612	0.449	1			
ABTS	−0.548	−0.669 *	0.229	1		
•OH	−0.197	−0.281	−0.160	0.354	1	
FRAP	0.887 **	0.962 **	0.404	−0.661	−0.288	1

Note: * Significant correlation (*p* < 0.05); ** highly significant correlation (*p* < 0.01).

**Table 4:** Chemical composition of PEF-Fr identified by GC-MS.

No.	Compound	Common Names	Molecular Formula	CAS No.	Relative Content (%)	Class
1	1-(2-hydroxyphenyl)-3-phenyl-1,3-propanedione	O-Hydroxydibenzoylmethane	C_15_H_12_O_3_	1469-94-9	1.52	Ketones
2	Isopropylamine	MIPA	C_3_H_9_N	75-31-0	11.84	Amines
3	(+)-Limonene ((4R)-limonene)	D-Limonene	C_10_H_16_	5989-27-5	1.91	Terpenoids
4	Linalool	Linalyl alcohol	C_10_H_18_O	78-70-6	3.45	Terpenoids
5	Neral (cis-Citral)	β-Citral	C_10_H_16_O	106-26-3	21.19	Terpenoids
6	Citral	-	C_10_H_16_O	5392-40-5	29.57	Terpenoids
7	Tetradecamethyl cycloheptasiloxane	Cycloheptasiloxane	C_14_H_42_O_7_Si_7_	107-50-6	3.24	Siloxanes
8	trans-Caryophyllene	β-Caryophyllene	C_15_H_24_	87-44-5	1.79	Terpenoids
9	Heptacosane	n-Heptacosane	C_27_H_56_	593-49-7	4.48	Alkanes
10	1,1′-[1,3-Propanediylbis(oxy)]bisoctadecane	1,3-Dioctadecyloxypropane	C_39_H_80_O_2_	17367-38-3	1.91	Ethers
11	1,1,3,3,5,5,7,7,9,9,11,11,13,13,15,15-Hexadecamethyloctasiloxane	Octasiloxane,1,1,3,3,5,5,7,7,9,9,11,11,13,13,15,15-hexadecamethyl	C_16_H_50_O_7_Si_8_	19095-24-0	1.82	Siloxanes
12	3-ethyl-5-(2-ethylbutyl)octadecane	Octadecane,3-ethyl-5-(2-ethylbutyl)-	C_26_H_54_	55282-12-7	3.36	Alkanes
13	1,1,3,3,5,5,7,7,9,9,11,11-Dodecamethylhexasiloxane	Hexasiloxane,1,1,3,3,5,5,7,7,9,9,11,11-dodecamethyl-	C_12_H_38_O_5_Si_6_	995-82-4	2.43	Siloxanes
14	1,1,3,3,5,5,7,7,9,9,11,12-Dodecamethylheptasiloxane	Heptasiloxane,1,1,3,3,5,5,7,7,9,9,11,12-dodecamethyl-	C_12_H_38_O_5_Si_7_	998-82-4	1.54	Siloxanes

Note: The total relative content of the identified compounds accounted for 90.05% of the total detected peaks.

**Table 5:** Chemical composition of NBF-Fr identified by GC-MS.

No.	Compound	Common Names	Molecular Formula	CAS No.	Relative Content (%)	Class
1	Ethyl nitrite	Nitrous ether	C_2_H_5_NO_2_	109-95-5	10.69	Esters
2	n-Butanol	Butyl alcohol	C_4_H_10_O	71-36-3	13.51	Alcohols
3	Butyl Acetate	Butyl ethanoate	C_6_H_12_O_2_	123-86-4	0.77	Esters
4	Linalool	Linalyl alcohol	C_10_H_18_O	78-70-6	3.13	Terpenoids
5	α-Terpineol	-	C_10_H_18_O	98-55-5	0.92	Terpenoids
6	Neral (cis-Citral)	β-Citral	C_10_H_16_O	106-26-3	19.02	Terpenoids
7	Citral	-	C_10_H_16_O	5392-40-5	27.55	Terpenoids
8	n-Decanoic acid	Capric acid	C_10_H_20_O_2_	334-48-5	18.44	Organic Acids
9	β-Caryophyllene	-	C_15_H_24_	13877-93-5	1.98	Terpenoids

Note: The total relative content of the identified compounds accounted for 96.01% of the total detected peaks.

## Data Availability

The dataset is available upon request from the authors.

## Supplementary Materials

The following supporting information can be downloaded at: https://www.mdpi.com/article/10.3390/molecules31091506/s1, File S1; File S2. Table S1: In vitro antioxidant activity of different solvent fractions from *Litsea cubeba* fruits.

## Abbreviations

The following abbreviations are used in this manuscript:

GC-MS	Gas chromatography–mass spectrometry
ROS	Reactive oxygen species
DPPH	2,2-Diphenyl-1-picrylhydrazyl
ABTS	2,2′-Azino-bis(3-ethylbenzothiazoline-6-sulfonic acid)
FRAP	Ferric reducing antioxidant power
TPC	Total phenolic content
TFC	Total flavonoid content
IC_50_	Half-maximal inhibitory concentration
LCE	*Litsea cubeba* ethanol extract
PEF	Petroleum ether fraction
EAF	Ethyl acetate fraction
NBF	n-Butanol fraction
AQF	Aqueous fraction
SD	Standard deviation
