# Supplementary material for: Comparative Antioxidant Evaluation and GC-MS Identification of Bioactive Constituents in Litsea cubeba (Lour.) Pers. Fractions
Source: Molecules. 2026 Apr 30;31(9):1506. doi: 10.3390/molecules31091506 (PMC13164878; doi:10.3390/molecules31091506)
Supplement: Supplementary file 1 [file molecules-31-01506-s001.zip › Supplementary File. S1.pdf]

Single Injection Report

**Data file:**	/TO2021/CL/Results/20250313 XCY 01 BS2021001.rslt\01 1.dx		
**Sample name:**	1		
**Description:**			
**Sample amount:**	0.000	**Sample type:**	Sample
**Instrument:**	BS2021001	**Location:**	102
**Injection date:**	2025-03-13 15:07:49+08:00	**Injection:**	1 of 1
**Acq. method:**	RS-GC-MS(Front) 02.amx	**Injection volume:**	0.010
**Analysis method:**	*RS-GC-MS 20250313 XCY 01 PM 01.pmx	**Acq. operator:**	chunyuan xia
**Last changed:**	2025-03-13 16:06:40+08:00		

Data Analysis Method: **RS-GC-MS 20250313 XCY 01 PM 01.pmx**

Path: **/TO2021/CL/Results/20250313 XCY 01 BS2021001.rslt**

Method Parameters

MS Library Search Parameters

Automatically search TIC peaks:	Yes
MS Library:	C:\NIST17\MSSEARCH\mainlib;C:\NIST17\MSSEARCH\replib;C:\NIST17\MSSEARCH\nist_msms;C:\NIST17\MSSEARCH\nist_msms2;C:\NIST17\MSSEARCH\nist_ri
Maximum number of hits returned:	3
Minimum spectrum match score:	600

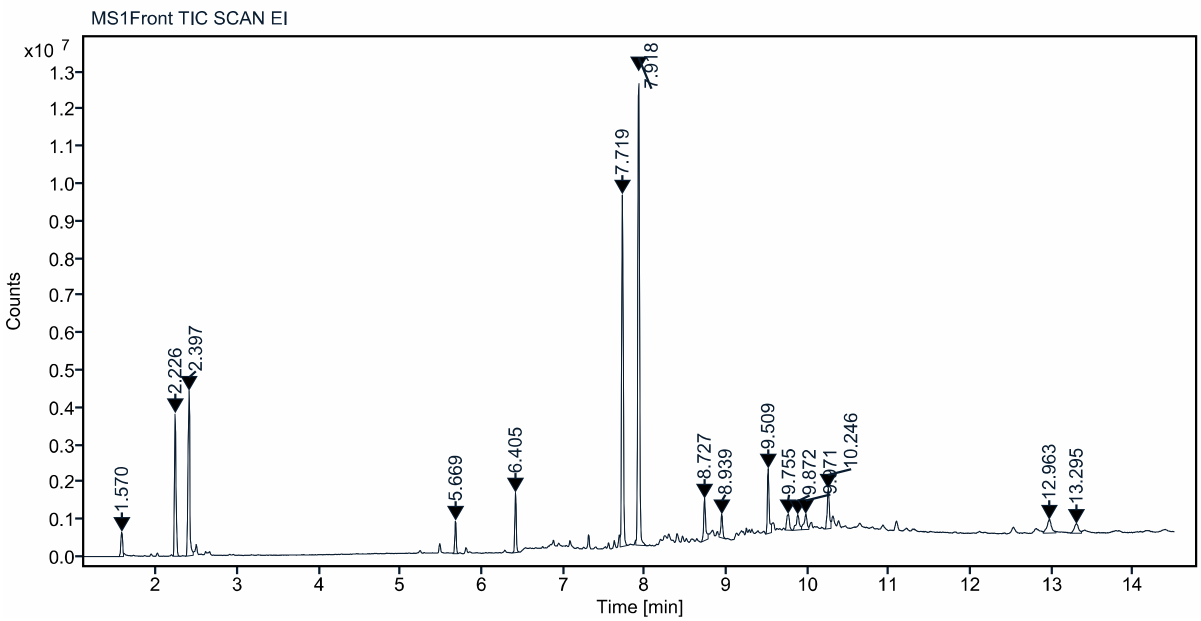

Peak @ 1.570 **Area** 903582.535 **Area %** 1.52

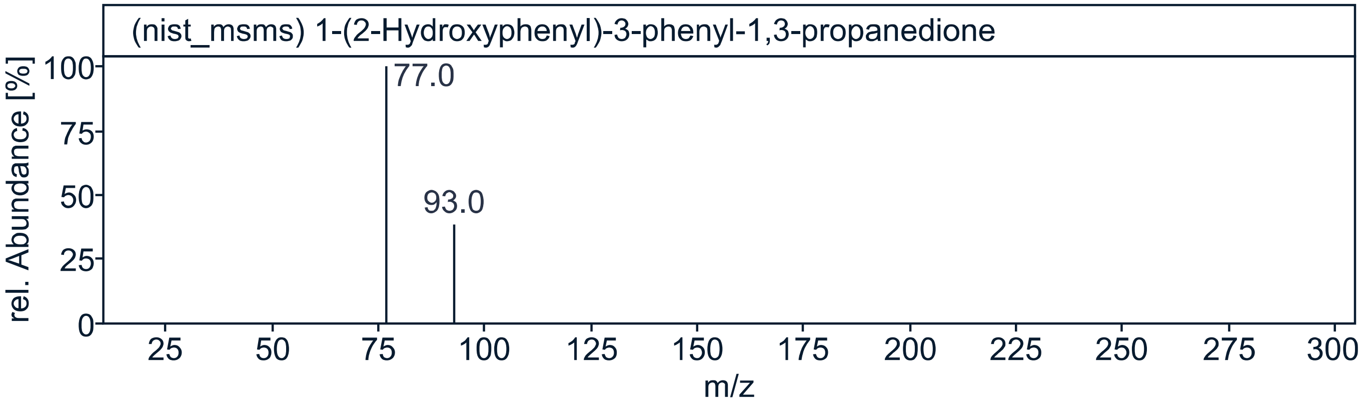

Ion Table

77.0 999 • 93.0 383

Compound Name	Score	Rev. Score	Prob. %	Library Name	CAS #	Library Id
1-(2-Hydroxyphenyl)-3-phenyl-1,3-propanedione	792	981	83.35	nist_msms	1469-94-9	493494

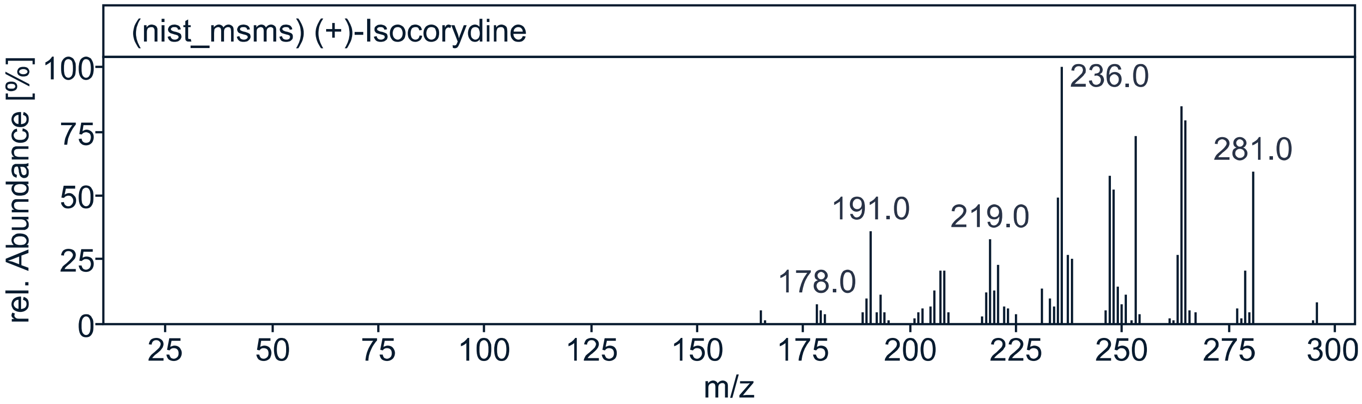

Ion Table

236.0 999 • 264.0 849 • 265.0 794 • 253.0 730 • 281.0 591 • 247.0 577

Compound Name	Score	Rev. Score	Prob. %	Library Name	CAS #	Library Id
(+)-Isocorydine	631	631	2.06	nist_msms	475-67-2	385251

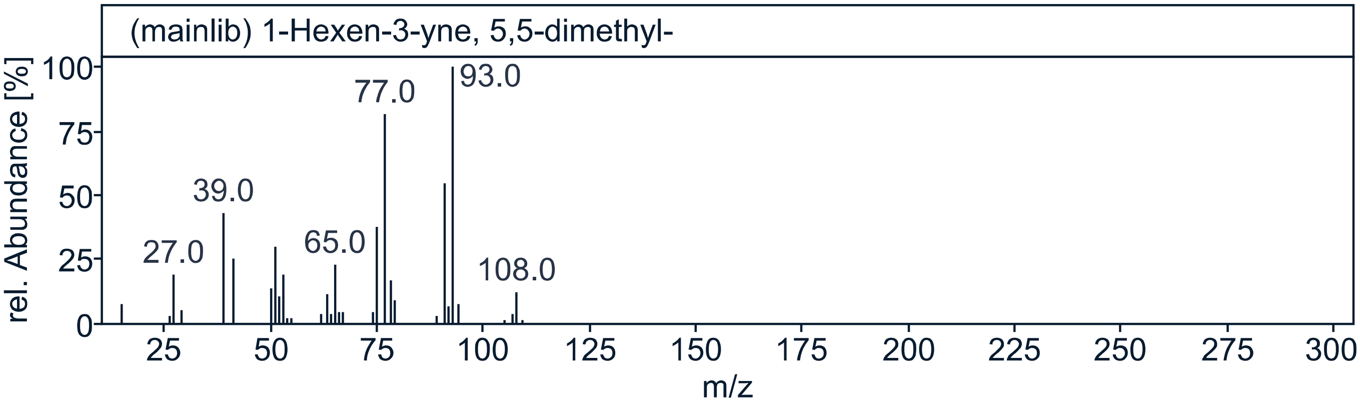

Ion Table

93.0 999 • 77.0 815 • 91.0 549 • 39.0 428 • 75.0 375 • 51.0 299

Compound Name	Score	Rev. Score	Prob. %	Library Name	CAS #	Library Id
1-Hexen-3-yne, 5,5-dimethyl-	628	735	1.82	mainlib	4911-58-4	72664

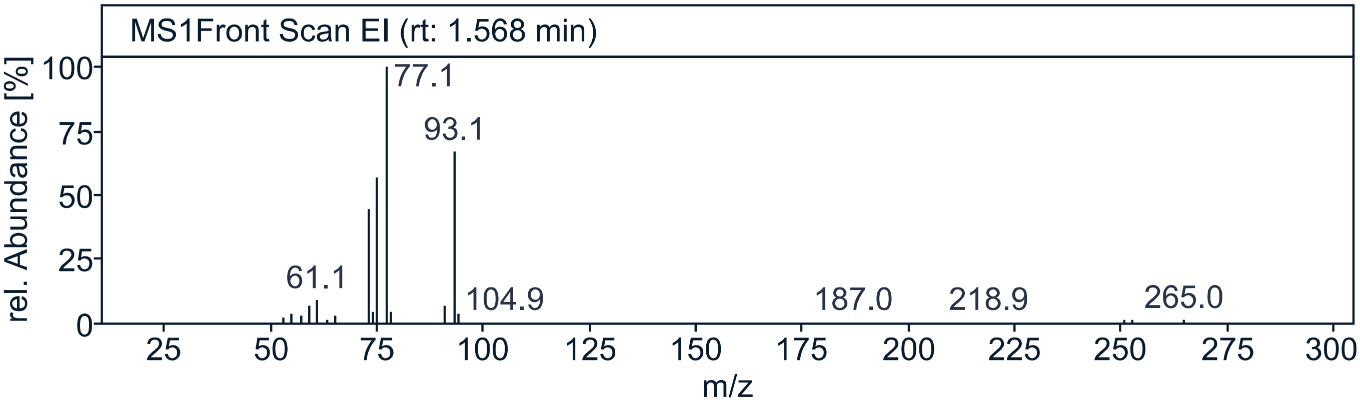

Ion Table

77.1 999 • 93.1 671 • 75.1 571 • 73.1 446 • 61.1 86 • 59.0 69

Summary Hit Table

Compound Name	Score	Rev. Score	Prob. %	Library Name	CAS #	Library Id
1-(2-Hydroxyphenyl)-3-phenyl-1,3-propanedione	792	981	83.35	nist_msms	1469-94-9	493494
(+)-Isocorydine	631	631	2.06	nist_msms	475-67-2	385251
1-Hexen-3-yne, 5,5-dimethyl-	628	735	1.82	mainlib	4911-58-4	72664

Peak @ 2.226 **Area** 4930634.936 **Area %** 8.32

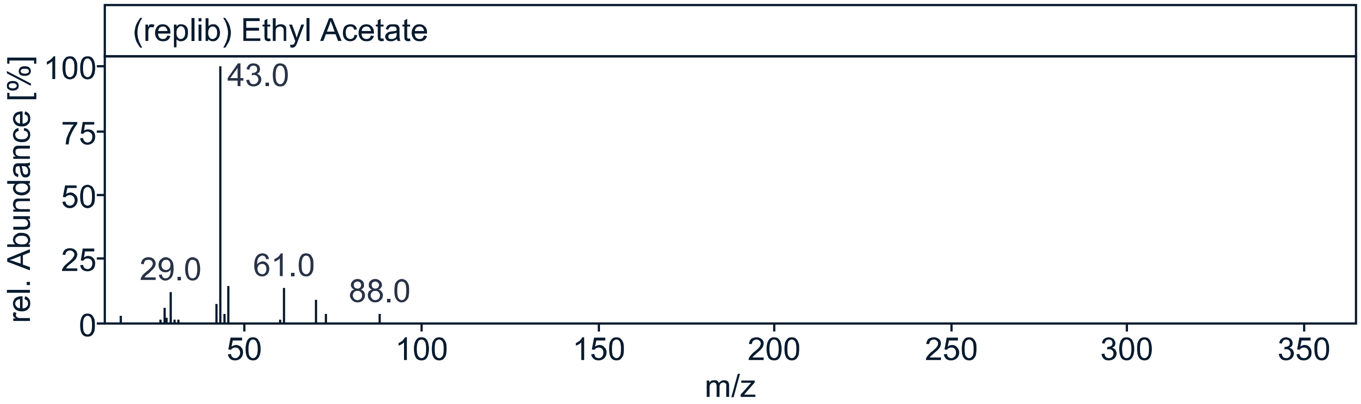

Ion Table

43.0 999 • 45.0 142 • 61.0 138 • 29.0 123 • 70.0 90 • 42.0 70

Compound Name	Score	Rev. Score	Prob. %	Library Name	CAS #	Library Id
Ethyl Acetate	922	947	96.78	replib	141-78-6	2336

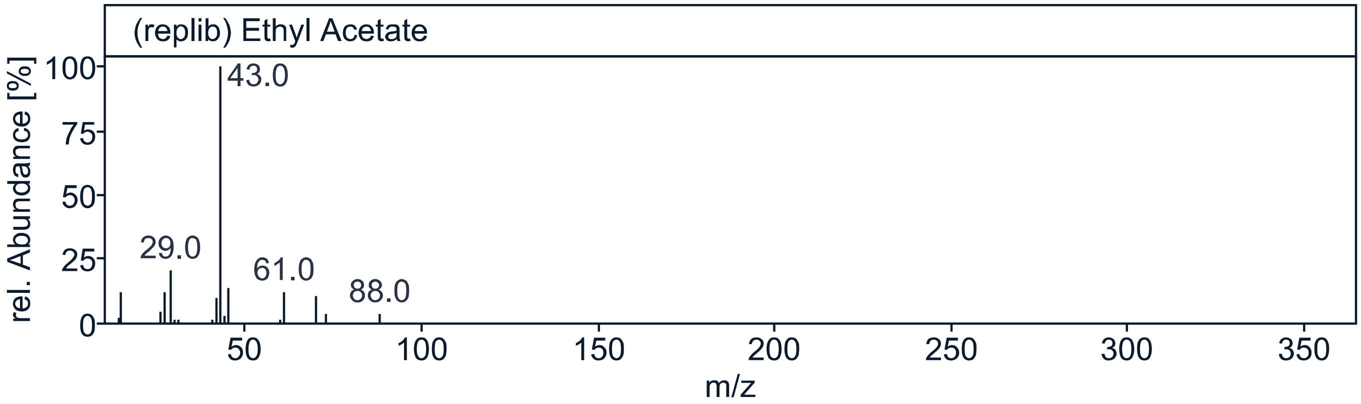

Ion Table

43.0 999 • 29.0 208 • 45.0 138 • 61.0 123 • 27.0 122 • 15.0 117

Compound Name	Score	Rev. Score	Prob. %	Library Name	CAS #	Library Id
Ethyl Acetate	912	952	96.78	replib	141-78-6	1961

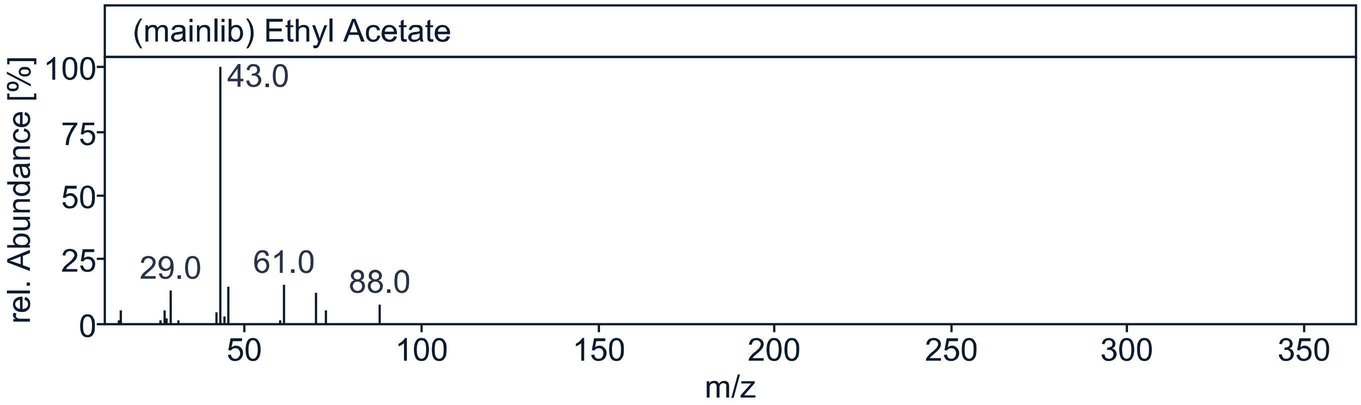

Ion Table

43.0 999 • 61.0 153 • 45.0 146 • 29.0 124 • 70.0 118 • 88.0 70

Compound Name	Score	Rev. Score	Prob. %	Library Name	CAS #	Library Id
Ethyl Acetate	895	906	96.78	mainlib	141-78-6	8863

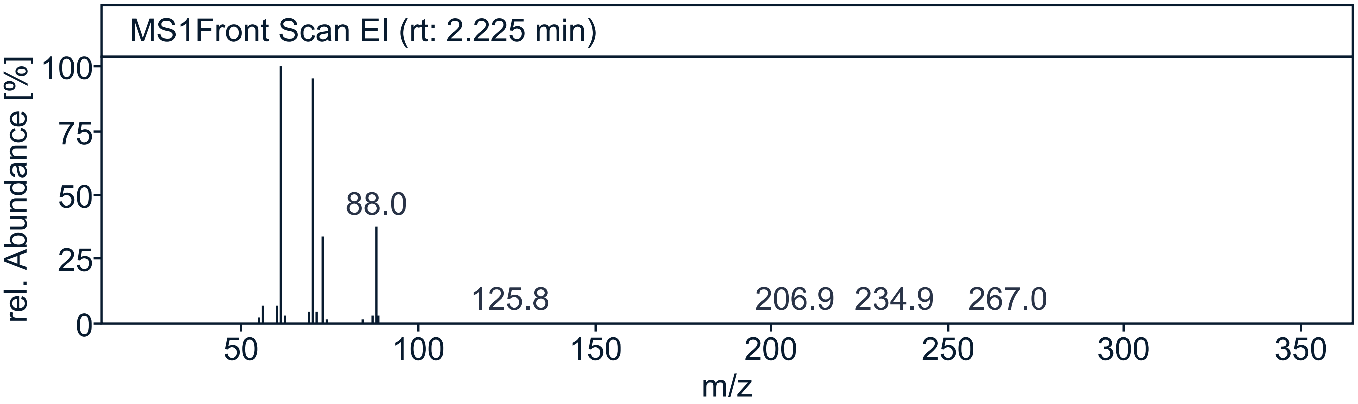

Ion Table

61.0 999 • 70.0 954 • 88.0 373 • 73.0 339 • 56.1 69 • 60.1 64

Summary Hit Table

Compound Name	Score	Rev. Score	Prob. %	Library Name	CAS #	Library Id
Ethyl Acetate	922	947	96.78	replib	141-78-6	2336
Ethyl Acetate	912	952	96.78	replib	141-78-6	1961
Ethyl Acetate	895	906	96.78	mainlib	141-78-6	8863

Peak @ 2.397 **Area** 7018732.058 **Area %** 11.84

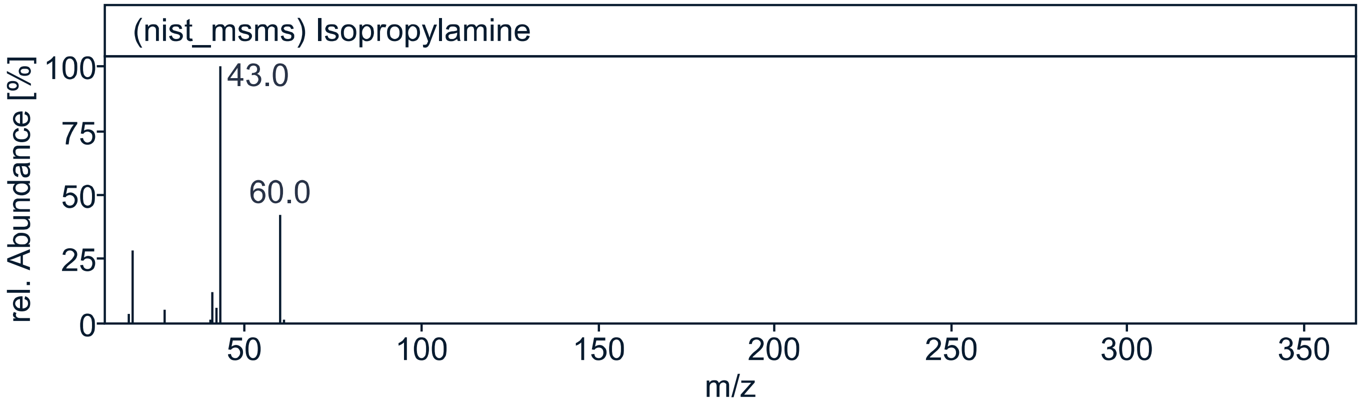

Ion Table

43.0 999 • 60.0 424 • 18.0 283 • 41.0 119 • 42.0 60 • 27.0 49

Compound Name	Score	Rev. Score	Prob. %	Library Name	CAS #	Library Id
Isopropylamine	987	992	7.22	nist_msms	75-31-0	943

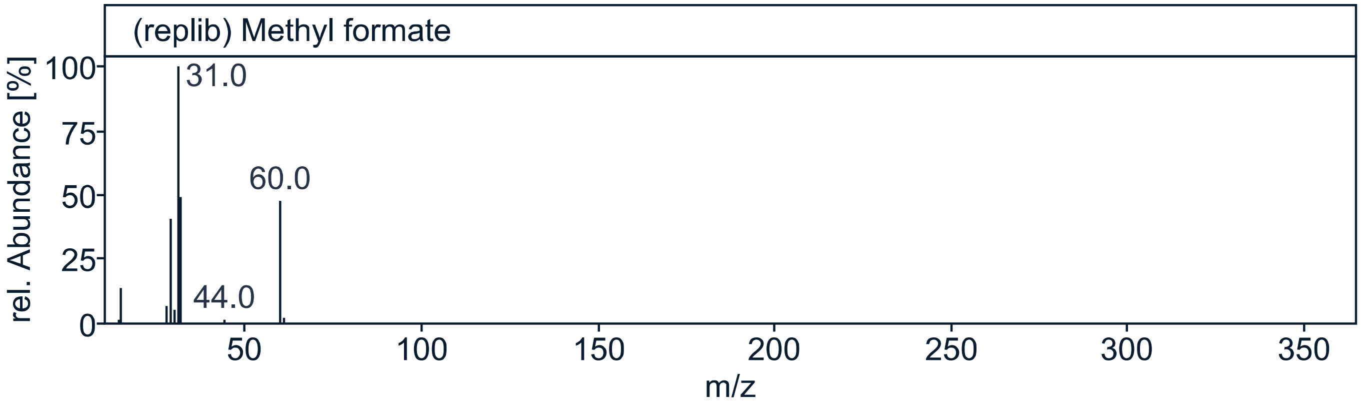

Ion Table

31.0 999 • 32.0 494 • 60.0 476 • 29.0 406 • 15.0 134 • 28.0 63

Compound Name	Score	Rev. Score	Prob. %	Library Name	CAS #	Library Id
Methyl formate	986	992	6.94	replib	107-31-3	702

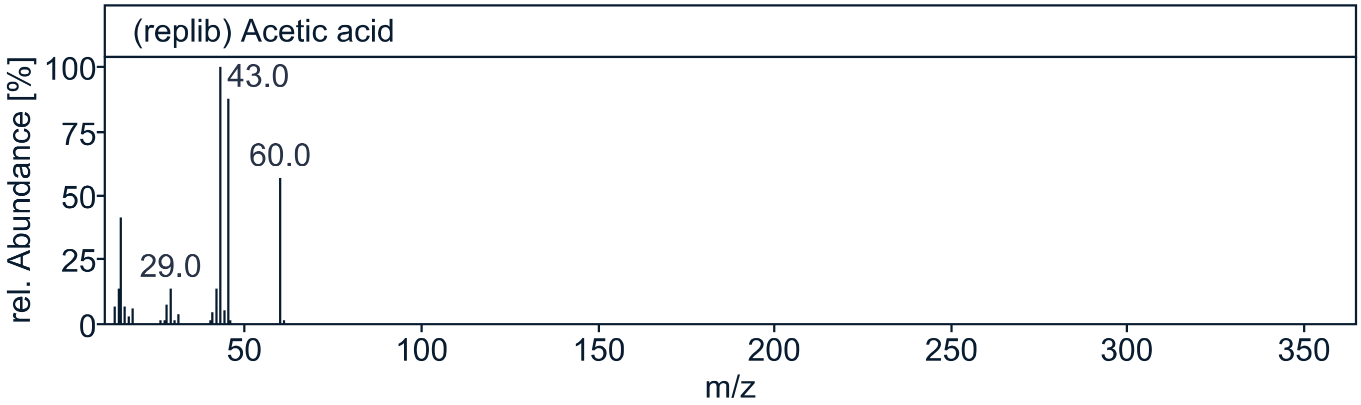

Ion Table

43.0 999 • 45.0 874 • 60.0 570 • 15.0 416 • 42.0 137 • 14.0 133

Compound Name	Score	Rev. Score	Prob. %	Library Name	CAS #	Library Id
Acetic acid	964	966	2.74	replib	64-19-7	2329

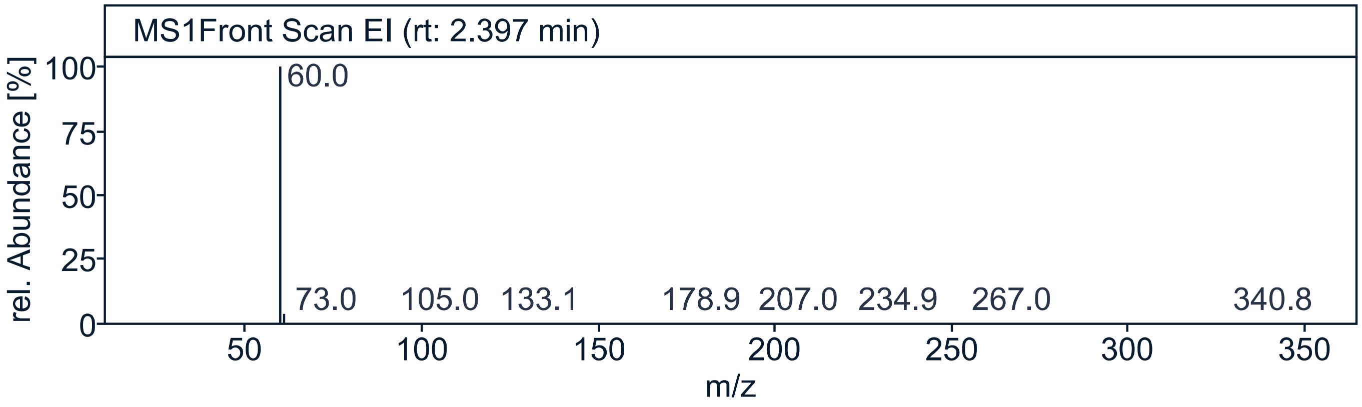

Ion Table

60.0 999 • 61.0 32 • 62.0 5 • 56.0 2 • 57.1 2 • 73.0 1

Summary Hit Table

Compound Name	Score	Rev. Score	Prob. %	Library Name	CAS #	Library Id
Isopropylamine	987	992	7.22	nist_msms	75-31-0	943
Methyl formate	986	992	6.94	replib	107-31-3	702
Acetic acid	964	966	2.74	replib	64-19-7	2329

Peak @ 5.669 **Area** 1132097.122 **Area %** 1.91

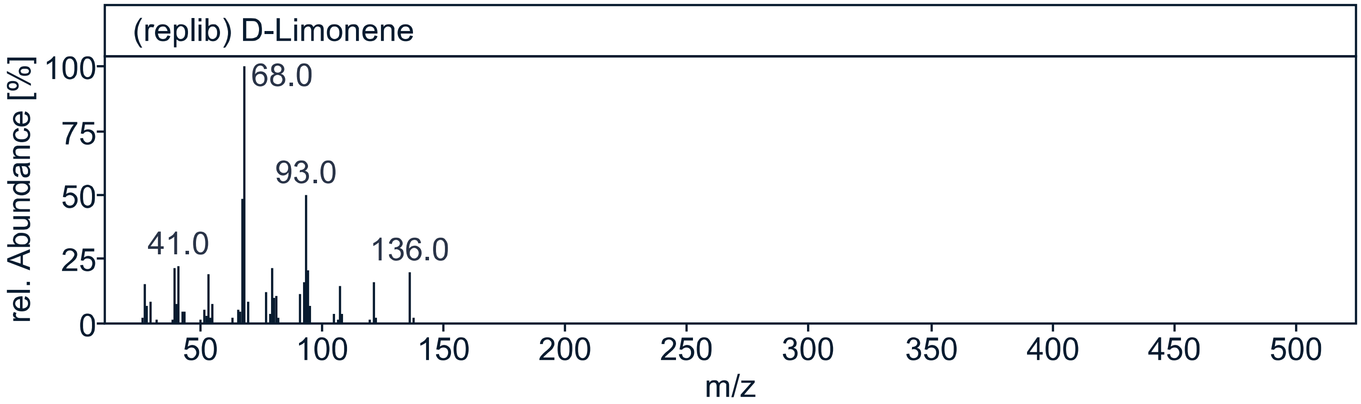

Ion Table

68.0 999 • 93.0 500 • 67.0 485 • 41.0 217 • 79.0 212 • 39.0 209

Compound Name	Score	Rev. Score	Prob. %	Library Name	CAS #	Library Id
D-Limonene	852	928	21.8	replib	5989-27-5	9643

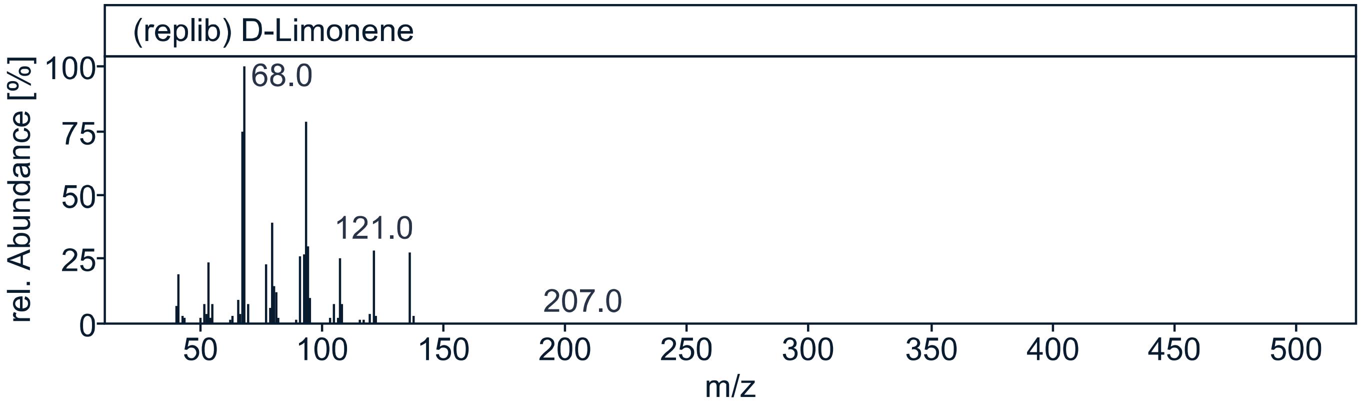

Ion Table

68.0 999 • 93.0 783 • 67.0 746 • 79.0 391 • 94.0 300 • 121.0 286

Compound Name	Score	Rev. Score	Prob. %	Library Name	CAS #	Library Id
D-Limonene	849	924	21.8	replib	5989-27-5	9644

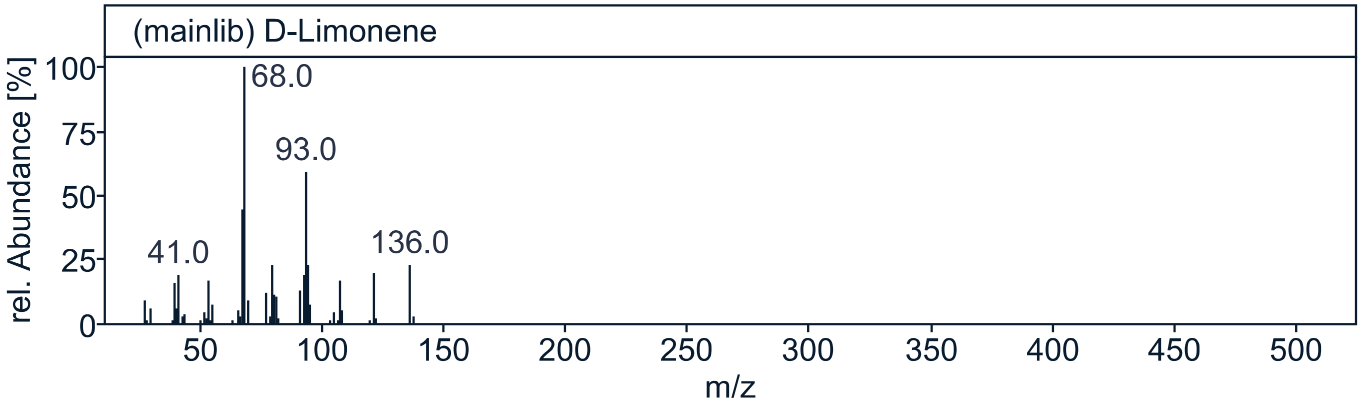

Ion Table

68.0 999 • 93.0 590 • 67.0 446 • 79.0 227 • 94.0 225 • 136.0 225

Compound Name	Score	Rev. Score	Prob. %	Library Name	CAS #	Library Id
D-Limonene	849	915	21.8	mainlib	5989-27-5	36349

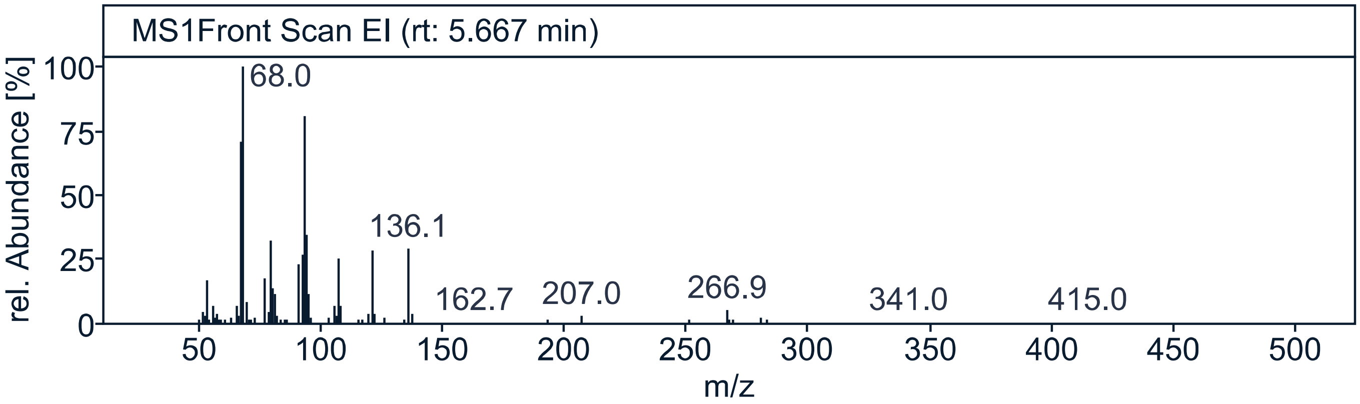

Ion Table

68.0 999 • 93.0 806 • 67.0 705 • 94.0 346 • 79.1 320 • 136.1 288

Summary Hit Table

Compound Name	Score	Rev. Score	Prob. %	Library Name	CAS #	Library Id
D-Limonene	852	928	21.8	replib	5989-27-5	9643
D-Limonene	849	924	21.8	replib	5989-27-5	9644
D-Limonene	849	915	21.8	mainlib	5989-27-5	36349

Peak @ 6.405 **Area** 2042035.142 **Area %** 3.45

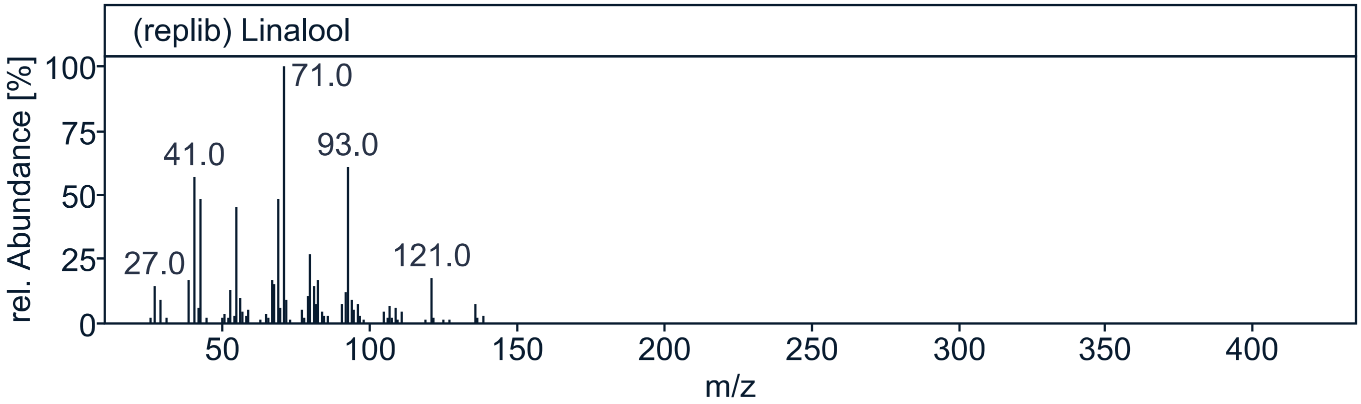

Ion Table

71.0 999 • 93.0 610 • 41.0 571 • 43.0 486 • 69.0 486 • 55.0 455

Compound Name	Score	Rev. Score	Prob. %	Library Name	CAS #	Library Id
Linalool	862	900	44.85	replib	78-70-6	10682

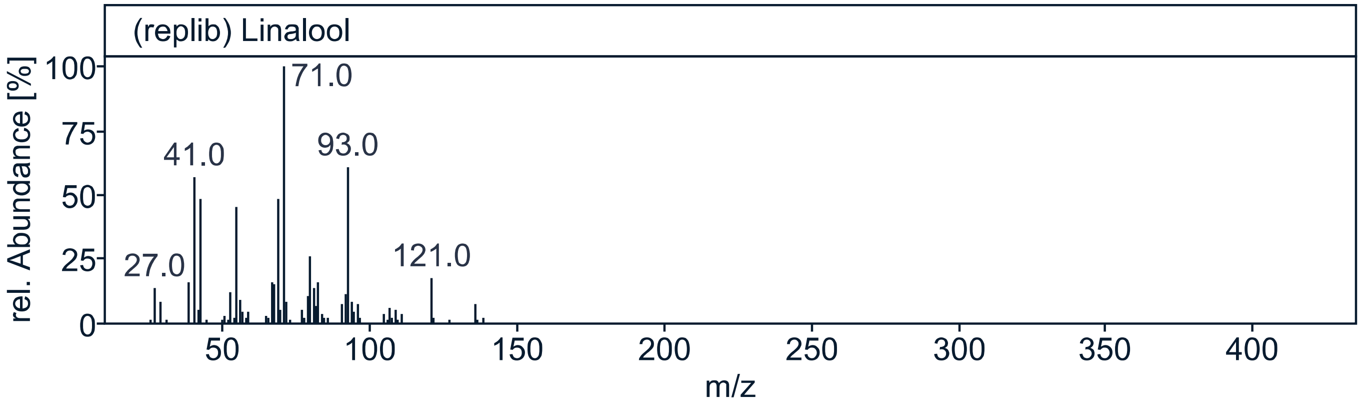

Ion Table

71.0 999 • 93.0 609 • 41.0 569 • 43.0 482 • 69.0 482 • 55.0 450

Compound Name	Score	Rev. Score	Prob. %	Library Name	CAS #	Library Id
Linalool	855	893	44.85	replib	78-70-6	10681

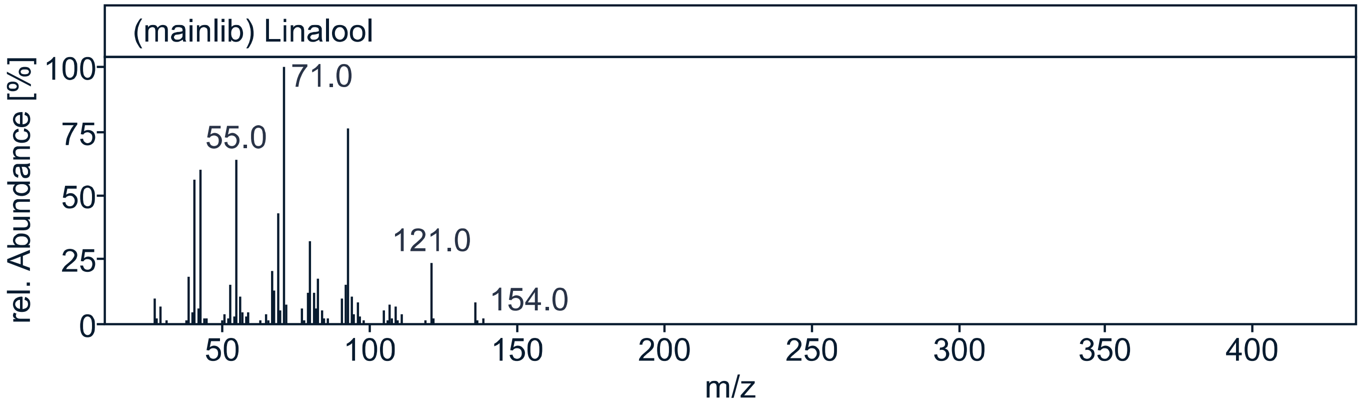

Ion Table

71.0 999 • 93.0 760 • 55.0 637 • 43.0 598 • 41.0 564 • 69.0 426

Compound Name	Score	Rev. Score	Prob. %	Library Name	CAS #	Library Id
Linalool	851	890	44.85	mainlib	78-70-6	42410

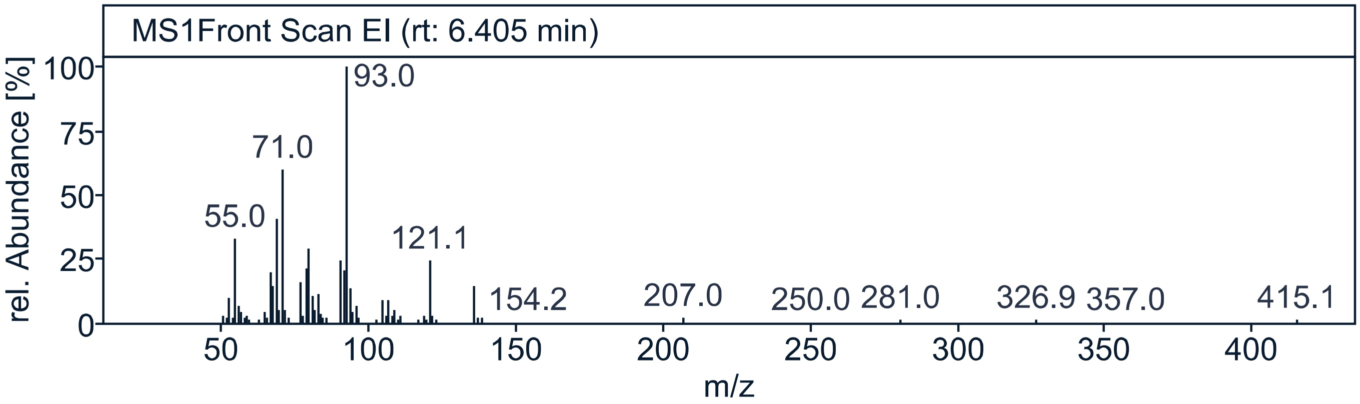

Ion Table

93.0 999 • 71.0 602 • 69.0 406 • 55.0 328 • 80.0 292 • 121.1 244

Summary Hit Table

Compound Name	Score	Rev. Score	Prob. %	Library Name	CAS #	Library Id
Linalool	862	900	44.85	replib	78-70-6	10682
Linalool	855	893	44.85	replib	78-70-6	10681
Linalool	851	890	44.85	mainlib	78-70-6	42410

Peak @ 7.719 **Area** 12561812.019 **Area %** 21.19

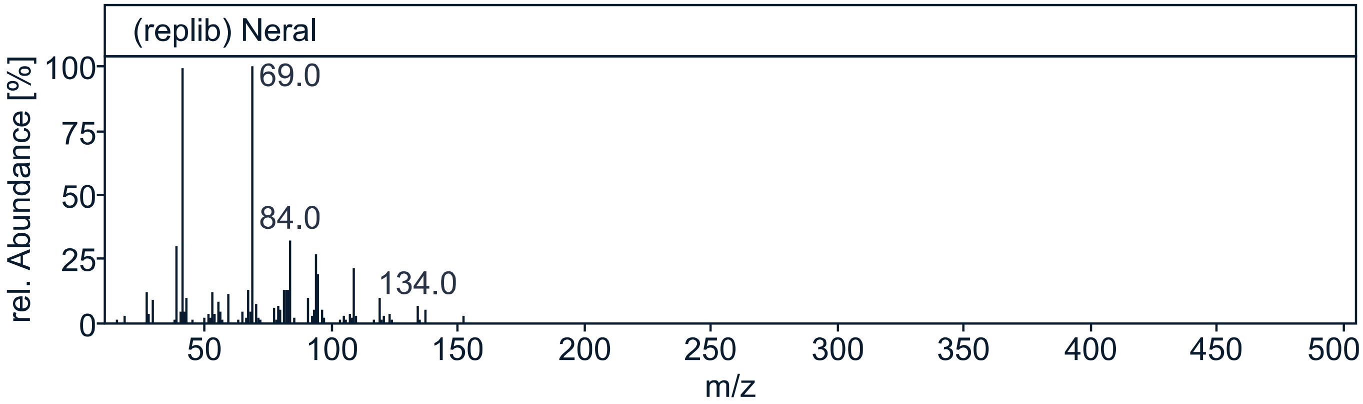

Ion Table

69.0 999 • 41.0 995 • 84.0 323 • 39.0 296 • 94.0 270 • 109.0 216

Compound Name	Score	Rev. Score	Prob. %	Library Name	CAS #	Library Id
Neral	909	915	43.5	replib	106-26-3	9833

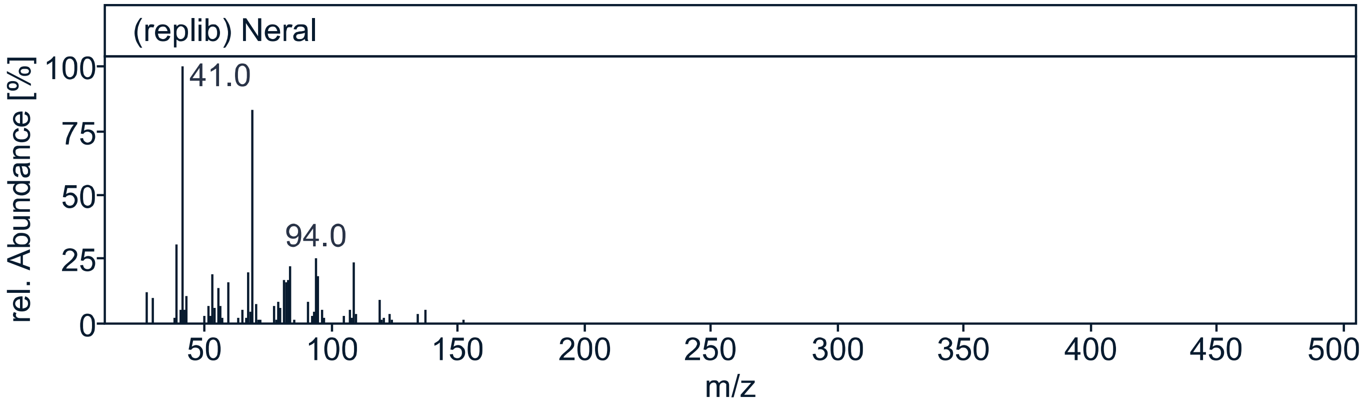

Ion Table

41.0 999 • 69.0 829 • 39.0 309 • 94.0 253 • 109.0 237 • 84.0 220

Compound Name	Score	Rev. Score	Prob. %	Library Name	CAS #	Library Id
Neral	898	908	43.5	replib	106-26-3	1334

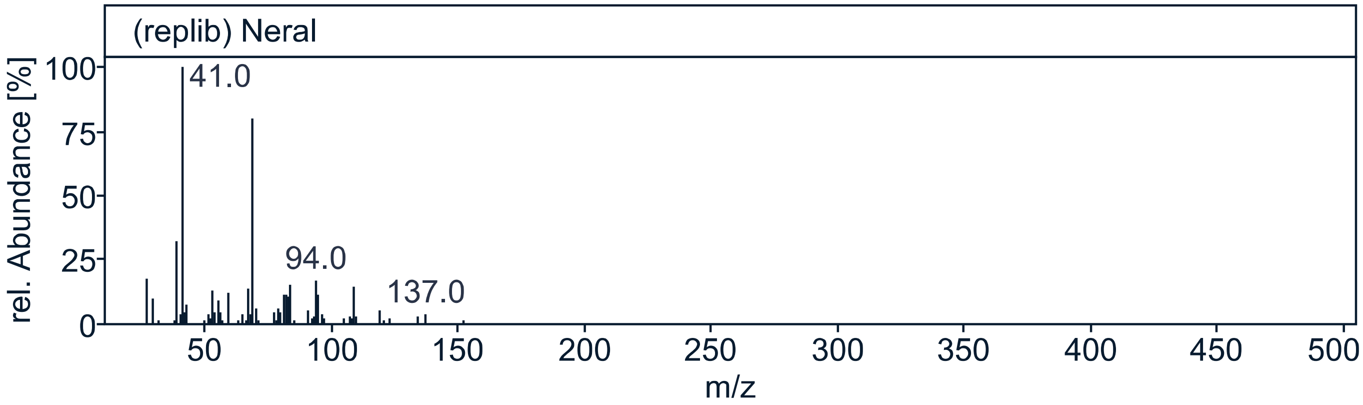

Ion Table

41.0 999 • 69.0 804 • 39.0 319 • 27.0 174 • 94.0 163 • 84.0 153

Compound Name	Score	Rev. Score	Prob. %	Library Name	CAS #	Library Id
Neral	893	941	43.5	replib	106-26-3	1335

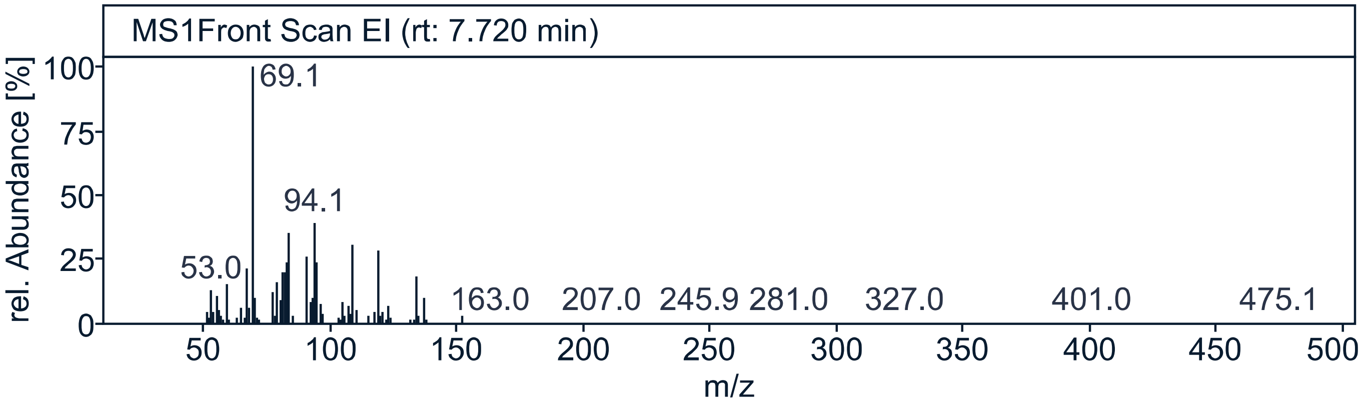

Ion Table

69.1 999 • 94.1 391 • 84.0 350 • 109.1 302 • 119.1 279 • 91.0 256

Summary Hit Table

Compound Name	Score	Rev. Score	Prob. %	Library Name	CAS #	Library Id
Neral	909	915	43.5	replib	106-26-3	9833
Neral	898	908	43.5	replib	106-26-3	1334
Neral	893	941	43.5	replib	106-26-3	1335

Peak @ 7.918 **Area** 17528587.981 **Area %** 29.57

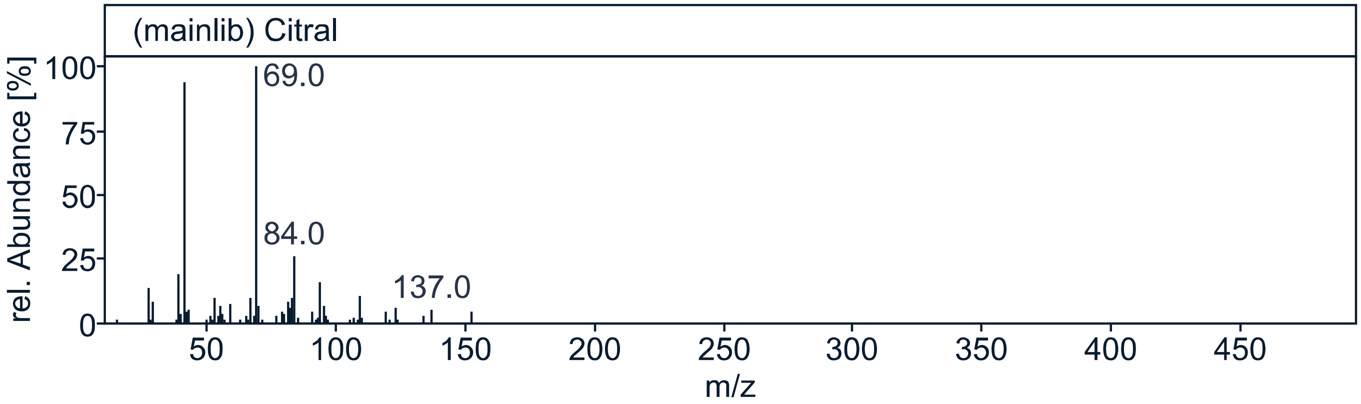

Ion Table

69.0 999 • 41.0 939 • 84.0 256 • 39.0 186 • 94.0 155 • 27.0 136

Compound Name	Score	Rev. Score	Prob. %	Library Name	CAS #	Library Id
Citral	925	932	42.43	mainlib	5392-40-5	37075

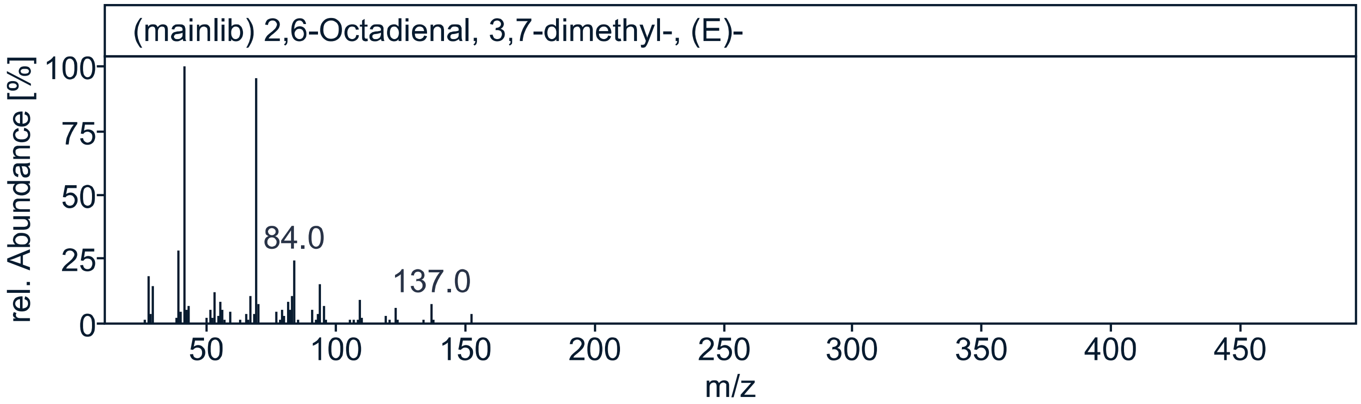

Ion Table

41.0 999 • 69.0 956 • 39.0 281 • 84.0 242 • 27.0 184 • 94.0 150

Compound Name	Score	Rev. Score	Prob. %	Library Name	CAS #	Library Id
2,6-Octadienal, 3,7-dimethyl-, (E)-	924	932	40.78	mainlib	141-27-5	3605

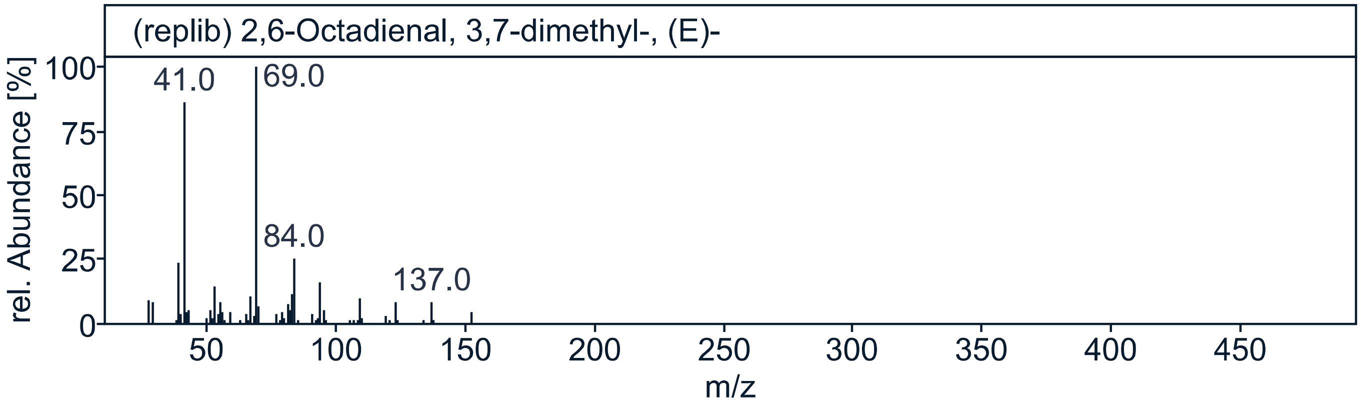

Ion Table

69.0 999 • 41.0 864 • 84.0 254 • 39.0 236 • 94.0 157 • 53.0 142

Compound Name	Score	Rev. Score	Prob. %	Library Name	CAS #	Library Id
2,6-Octadienal, 3,7-dimethyl-, (E)-	923	930	40.78	replib	141-27-5	9838

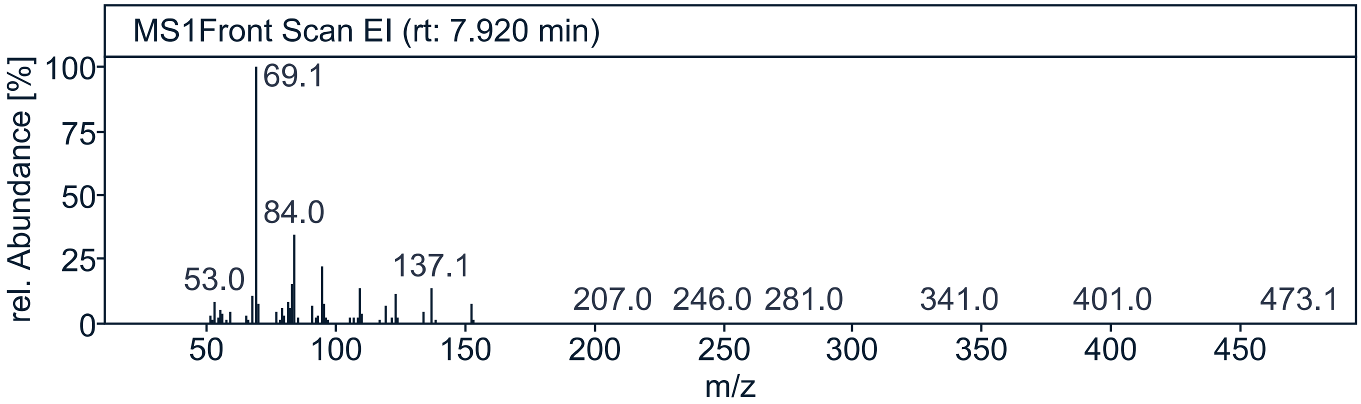

Ion Table

69.1 999 • 84.0 343 • 94.1 220 • 83.1 147 • 109.1 138 • 137.1 137

Summary Hit Table

Compound Name	Score	Rev. Score	Prob. %	Library Name	CAS #	Library Id
Citral	925	932	42.43	mainlib	5392-40-5	37075
2,6-Octadienal, 3,7-dimethyl-, (E)-	924	932	40.78	mainlib	141-27-5	3605
2,6-Octadienal, 3,7-dimethyl-, (E)-	923	930	40.78	replib	141-27-5	9838

Peak @ 8.727 **Area** 1921939.983 **Area %** 3.24

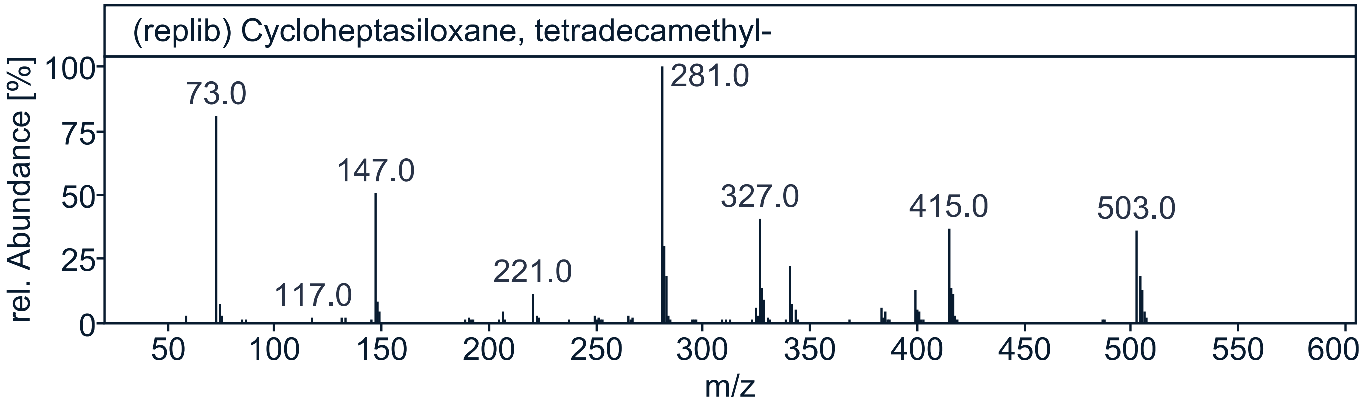

Ion Table

281.0 999 • 73.0 809 • 147.0 509 • 327.0 409 • 415.0 369 • 503.0 359

Compound Name	Score	Rev. Score	Prob. %	Library Name	CAS #	Library Id
Cycloheptasiloxane, tetradecamethyl-	640	834	10.86	replib	107-50-6	37342

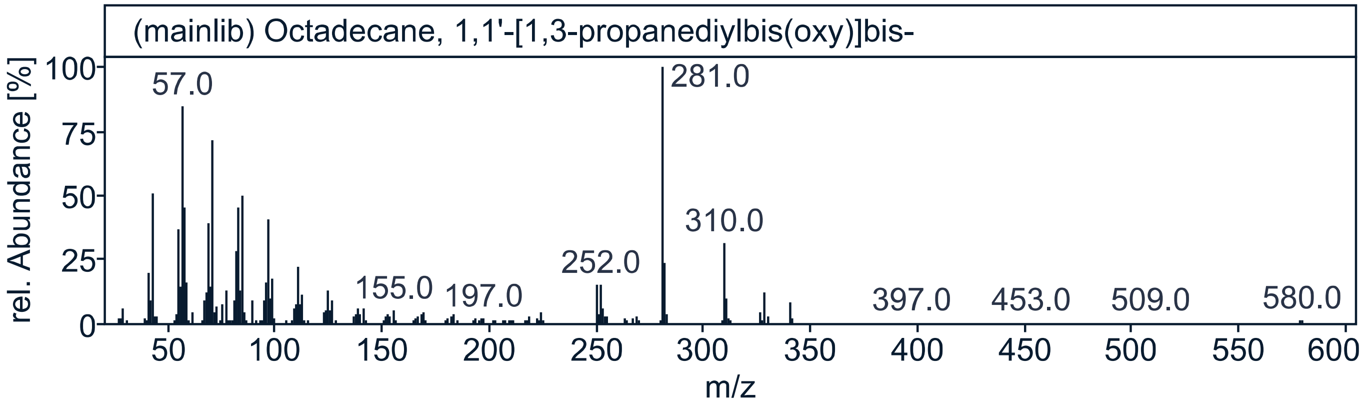

Ion Table

281.0 999 • 57.0 844 • 71.0 715 • 43.0 505 • 85.0 500 • 58.0 451

Compound Name	Score	Rev. Score	Prob. %	Library Name	CAS #	Library Id
Octadecane, 1,1'-[1,3-propanediylbis(oxy)]bis-	635	656	8.75	mainlib	17367-38-3	241948

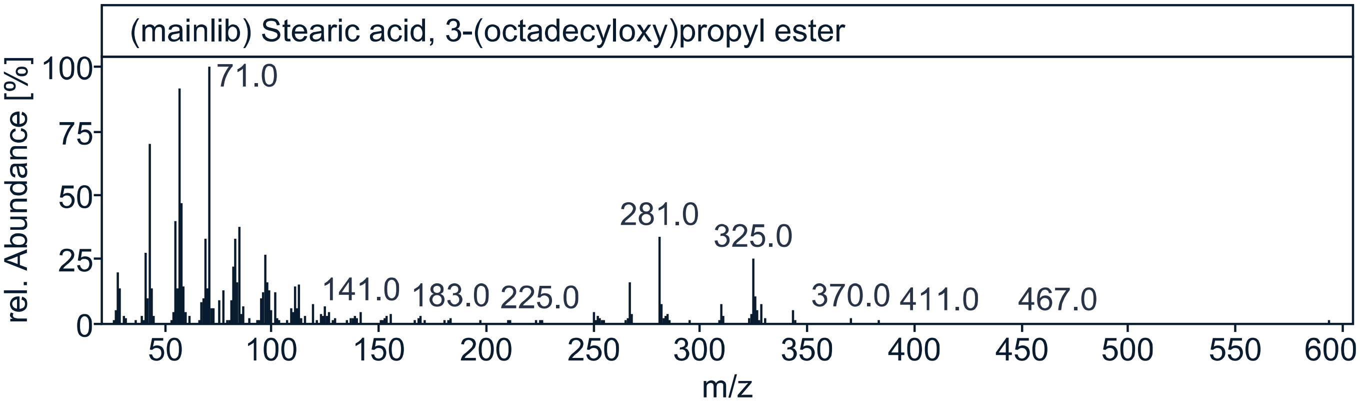

Ion Table

71.0 999 • 57.0 920 • 43.0 698 • 58.0 468 • 55.0 396 • 85.0 373

Compound Name	Score	Rev. Score	Prob. %	Library Name	CAS #	Library Id
Stearic acid, 3-(octadecyloxy)propyl ester	621	664	5.48	mainlib	17367-40-7	42013

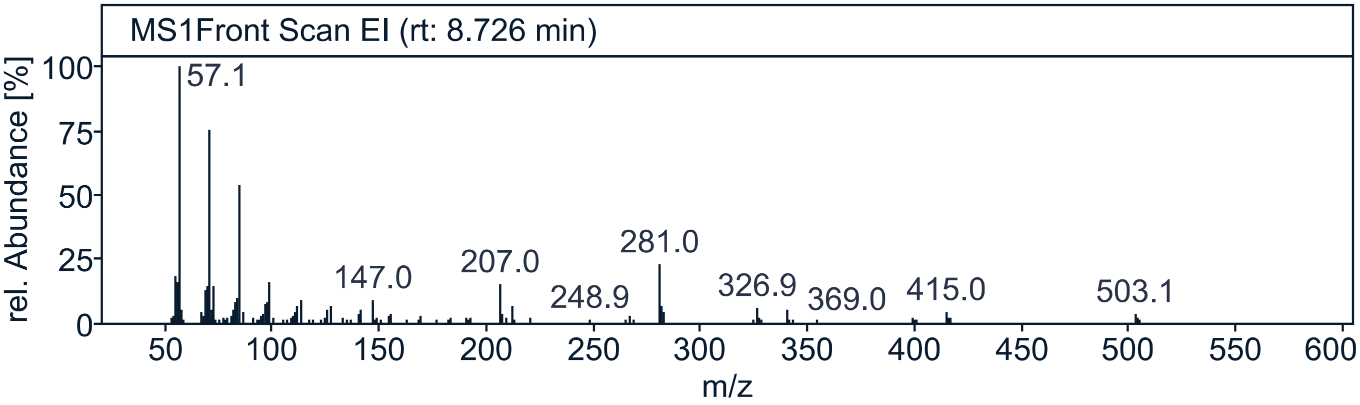

Ion Table

57.1 999 • 71.1 754 • 85.1 539 • 281.0 227 • 55.0 178 • 99.1 160

Summary Hit Table

Compound Name	Score	Rev. Score	Prob. %	Library Name	CAS #	Library Id
Cycloheptasiloxane, tetradecamethyl-	640	834	10.86	replib	107-50-6	37342
Octadecane, 1,1'-[1,3-propanediylbis(oxy)]bis-	635	656	8.75	mainlib	17367-38-3	241948
Stearic acid, 3-(octadecyloxy)propyl ester	621	664	5.48	mainlib	17367-40-7	42013

Peak @ 8.939 **Area** 1058484.971 **Area %** 1.79

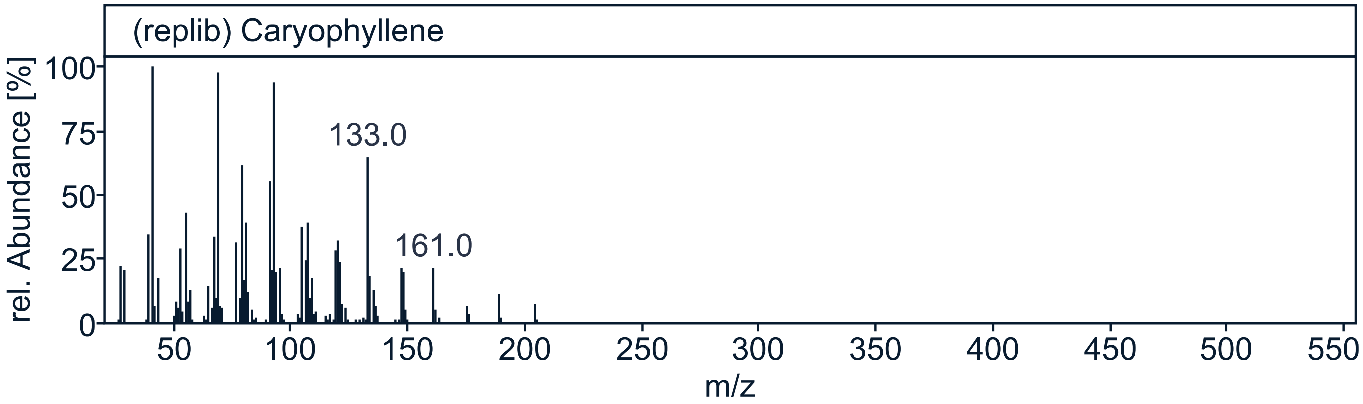

Ion Table

41.0 999 • 69.0 976 • 93.0 937 • 133.0 646 • 79.0 614 • 91.0 551

Compound Name	Score	Rev. Score	Prob. %	Library Name	CAS #	Library Id
Caryophyllene	662	885	30.86	replib	87-44-5	1425

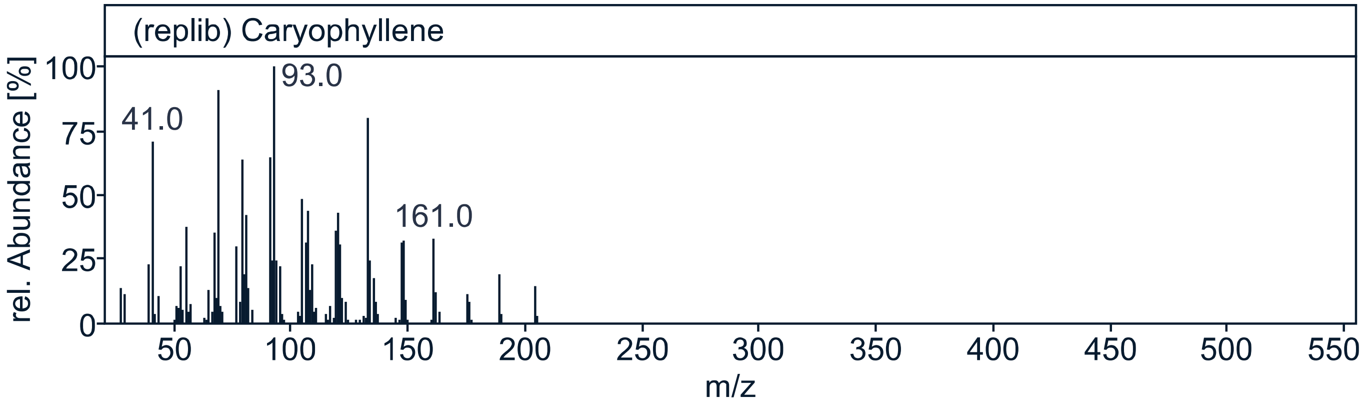

Ion Table

93.0 999 • 69.0 908 • 133.0 802 • 41.0 710 • 91.0 648 • 79.0 640

Compound Name	Score	Rev. Score	Prob. %	Library Name	CAS #	Library Id
Caryophyllene	633	810	30.86	replib	87-44-5	16194

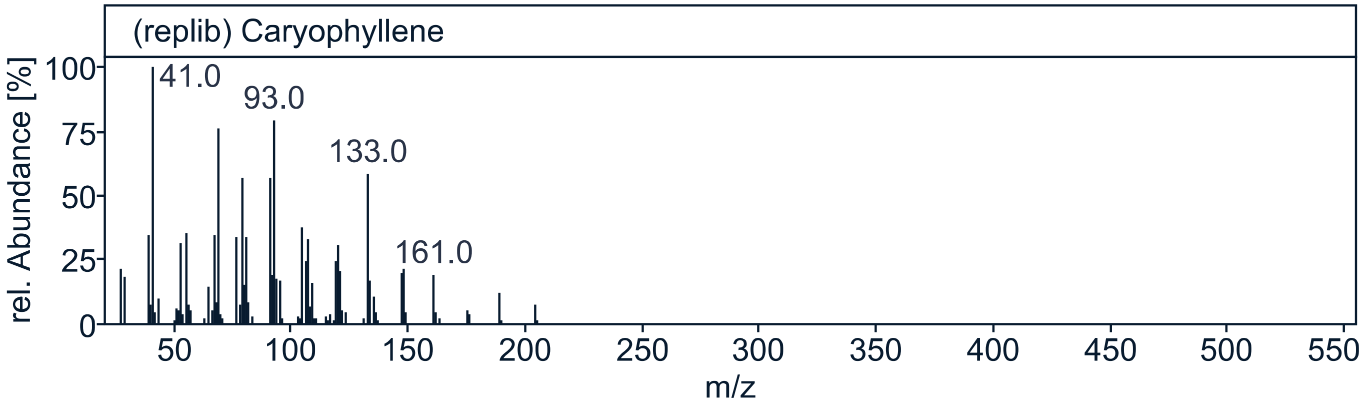

Ion Table

41.0 999 • 93.0 789 • 69.0 759 • 133.0 582 • 79.0 571 • 91.0 565

Compound Name	Score	Rev. Score	Prob. %	Library Name	CAS #	Library Id
Caryophyllene	629	888	30.86	replib	87-44-5	1517

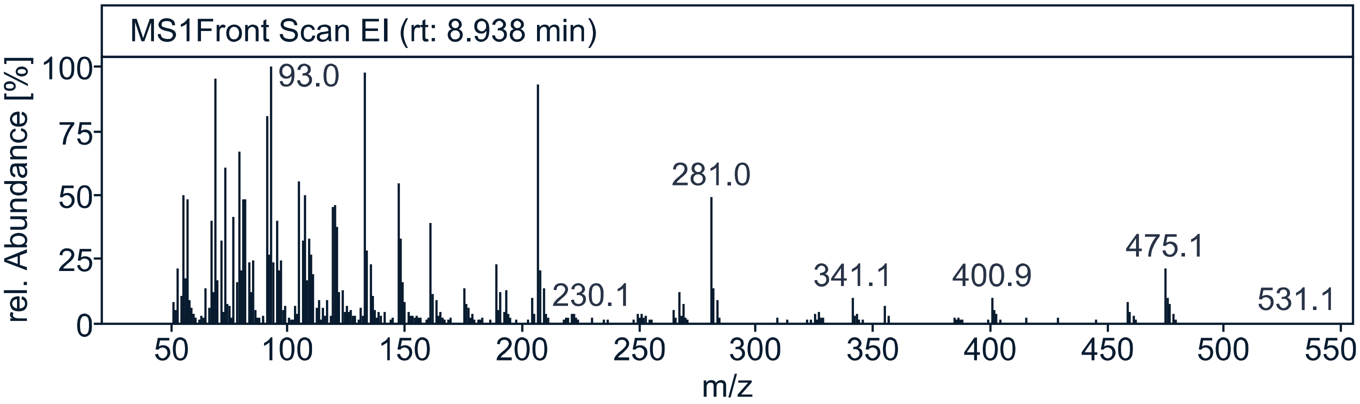

Ion Table

93.0 999 • 133.1 975 • 69.1 954 • 207.0 930 • 91.0 810 • 79.0 672

Summary Hit Table

Compound Name	Score	Rev. Score	Prob. %	Library Name	CAS #	Library Id
Caryophyllene	662	885	30.86	replib	87-44-5	1425
Caryophyllene	633	810	30.86	replib	87-44-5	16194
Caryophyllene	629	888	30.86	replib	87-44-5	1517

Peak @ 9.509 **Area** 2657824.001 **Area %** 4.48

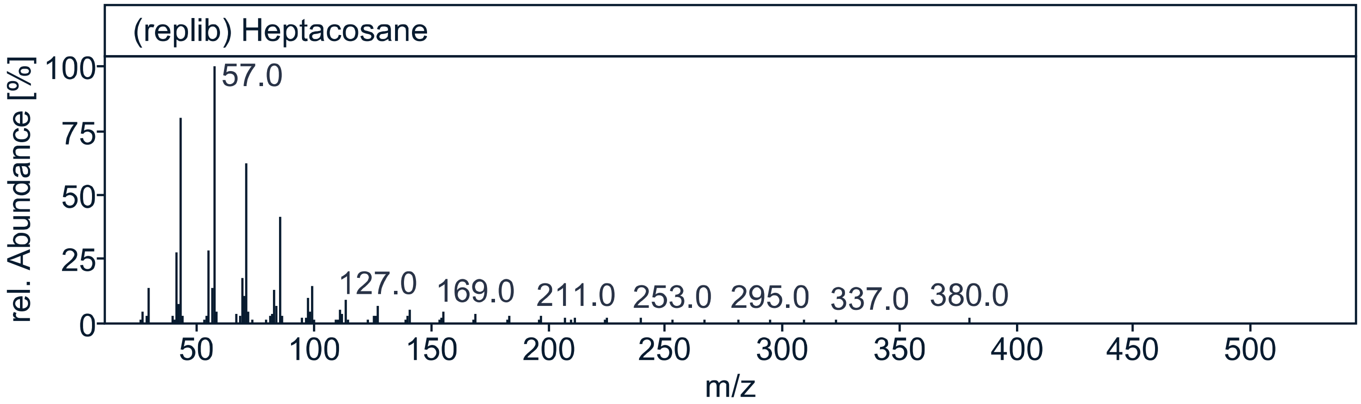

Ion Table

57.0 999 • 43.0 798 • 71.0 622 • 85.0 416 • 55.0 283 • 41.0 275

Compound Name	Score	Rev. Score	Prob. %	Library Name	CAS #	Library Id
Heptacosane	744	791	13.37	replib	593-49-7	7039

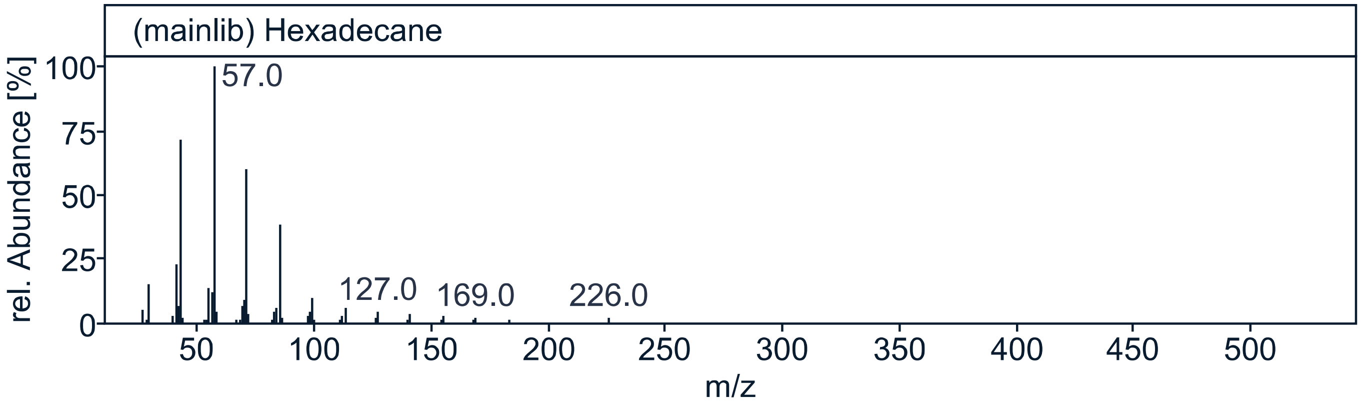

Ion Table

57.0 999 • 43.0 719 • 71.0 603 • 85.0 385 • 41.0 231 • 29.0 151

Compound Name	Score	Rev. Score	Prob. %	Library Name	CAS #	Library Id
Hexadecane	738	916	10.51	mainlib	544-76-3	26289

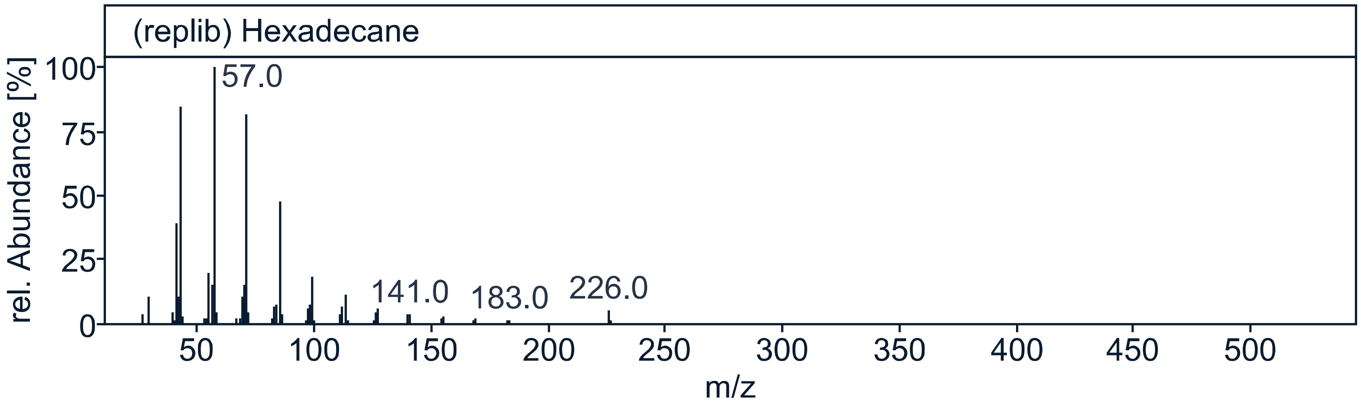

Ion Table

57.0 999 • 43.0 845 • 71.0 817 • 85.0 474 • 41.0 394 • 55.0 201

Compound Name	Score	Rev. Score	Prob. %	Library Name	CAS #	Library Id
Hexadecane	730	912	10.51	replib	544-76-3	7097

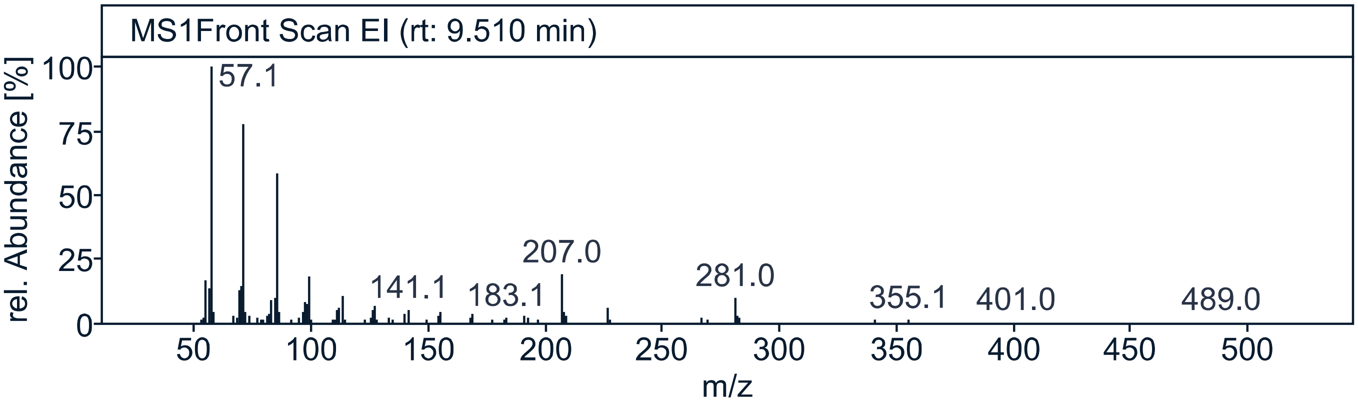

Ion Table

57.1 999 • 71.1 776 • 85.1 581 • 207.0 190 • 99.1 181 • 55.0 167

Summary Hit Table

Compound Name	Score	Rev. Score	Prob. %	Library Name	CAS #	Library Id
Heptacosane	744	791	13.37	replib	593-49-7	7039
Hexadecane	738	916	10.51	mainlib	544-76-3	26289
Hexadecane	730	912	10.51	replib	544-76-3	7097

Peak @ 9.755 **Area** 961114.154 **Area %** 1.62

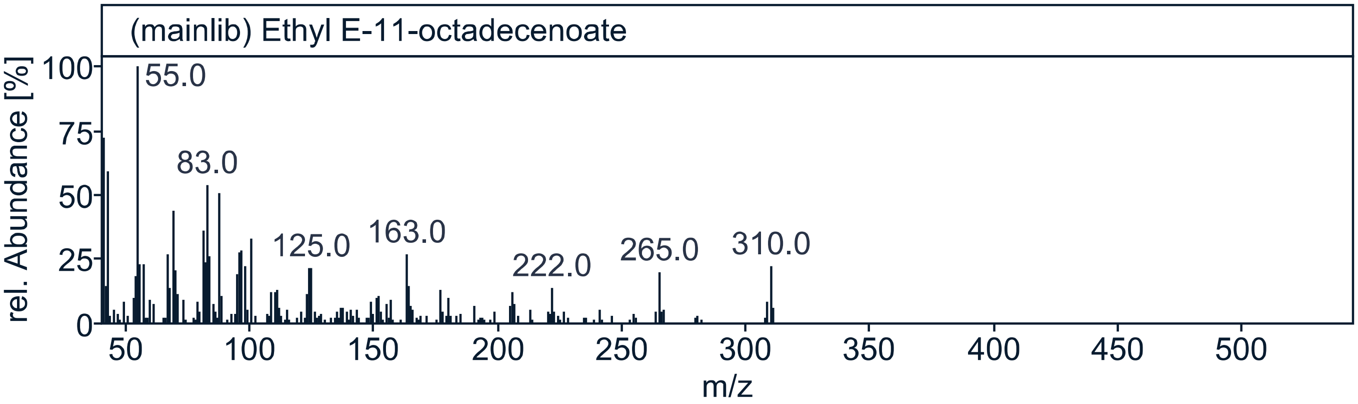

Ion Table

55.0 999 • 41.0 725 • 43.0 589 • 83.0 539 • 88.0 508 • 69.0 434

Compound Name	Score	Rev. Score	Prob. %	Library Name	CAS #	Library Id
Ethyl E-11-octadecenoate	604	661	13.48	mainlib		20622

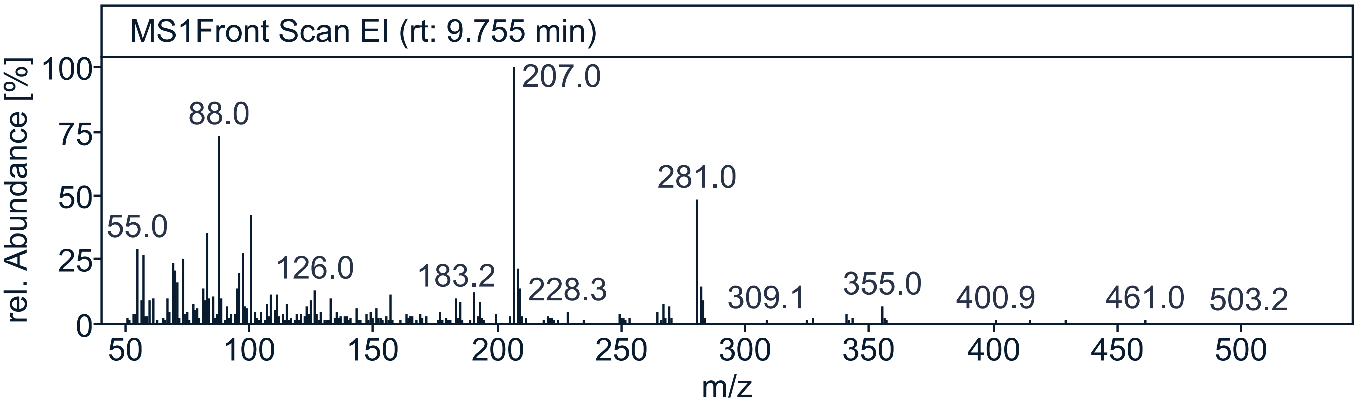

Ion Table

207.0 999 • 88.0 732 • 281.0 480 • 101.0 418 • 83.1 349 • 55.0 291

Summary Hit Table

Compound Name	Score	Rev. Score	Prob. %	Library Name	CAS #	Library Id
Ethyl E-11-octadecenoate	604	661	13.48	mainlib		20622

Peak @ 9.872 **Area** 1130463.337 **Area %** 1.91

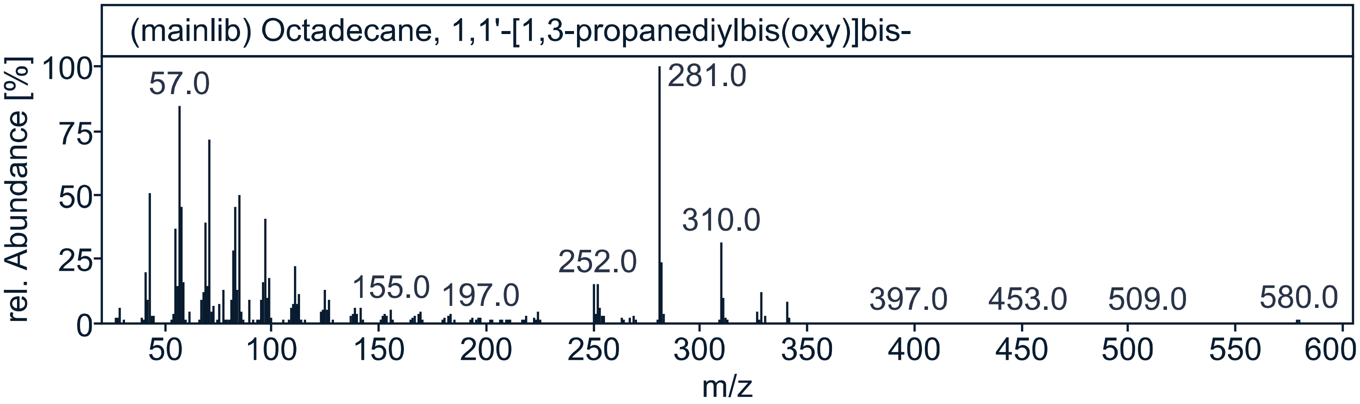

Ion Table

281.0 999 • 57.0 844 • 71.0 715 • 43.0 505 • 85.0 500 • 58.0 451

Compound Name	Score	Rev. Score	Prob. %	Library Name	CAS #	Library Id
Octadecane, 1,1'-[1,3-propanediylbis(oxy)]bis-	644	647	23.93	mainlib	17367-38-3	241948

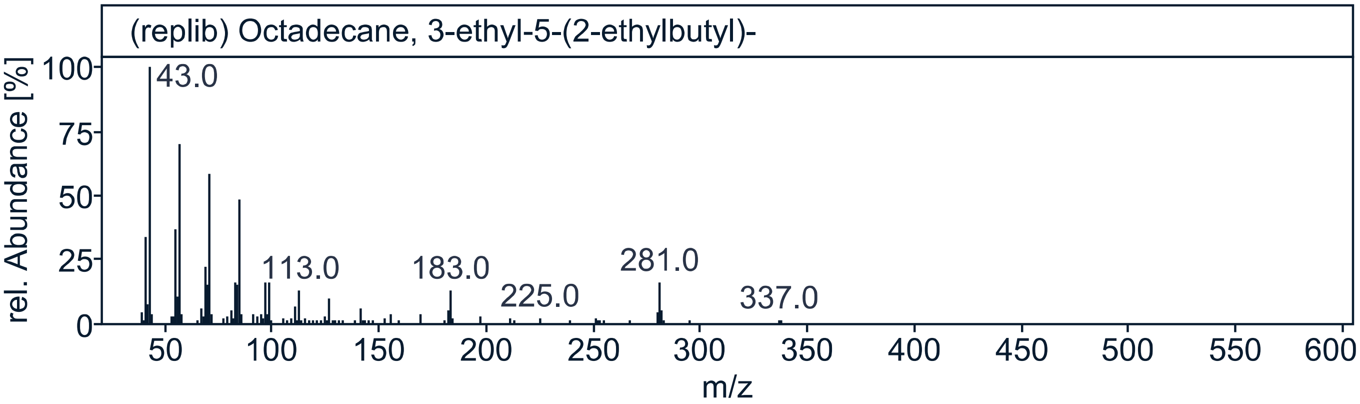

Ion Table

43.0 999 • 57.0 700 • 71.0 582 • 85.0 484 • 55.0 370 • 41.0 336

Compound Name	Score	Rev. Score	Prob. %	Library Name	CAS #	Library Id
Octadecane, 3-ethyl-5-(2-ethylbutyl)-	619	650	7.3	replib	55282-12-7	2669

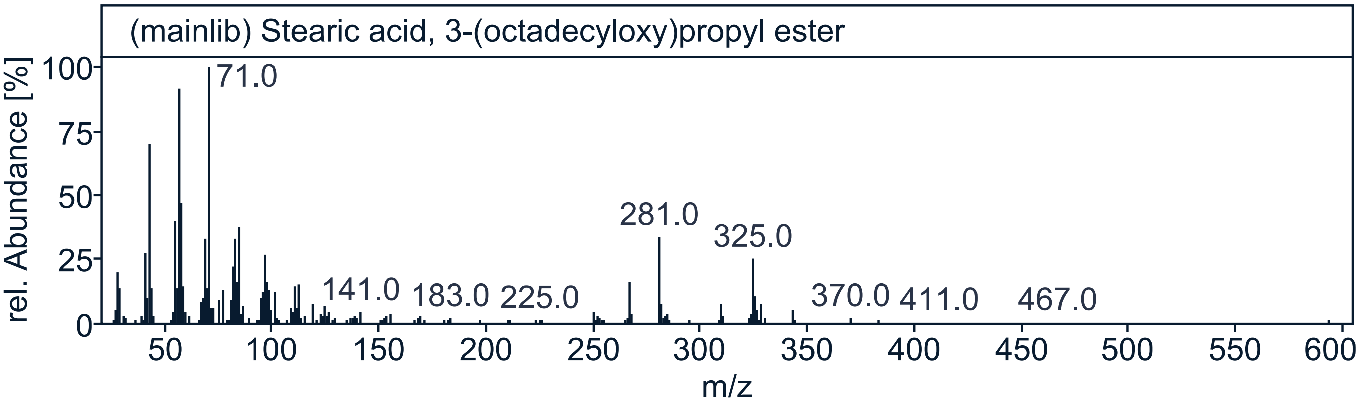

Ion Table

71.0 999 • 57.0 920 • 43.0 698 • 58.0 468 • 55.0 396 • 85.0 373

Compound Name	Score	Rev. Score	Prob. %	Library Name	CAS #	Library Id
Stearic acid, 3-(octadecyloxy)propyl ester	618	625	7.01	mainlib	17367-40-7	42013

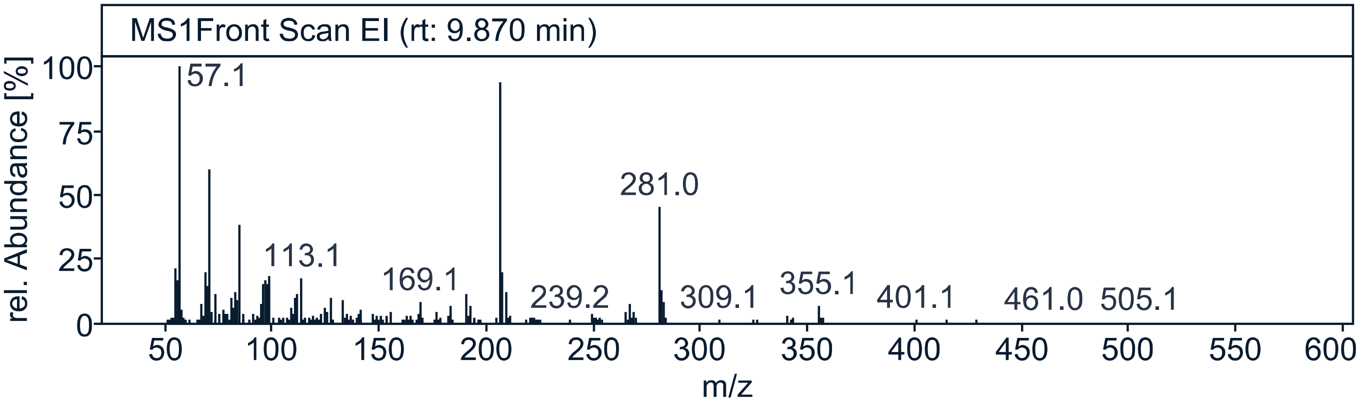

Ion Table

57.1 999 • 207.0 938 • 71.1 603 • 281.0 451 • 85.1 381 • 55.1 215

Summary Hit Table

Compound Name	Score	Rev. Score	Prob. %	Library Name	CAS #	Library Id
Octadecane, 1,1'-[1,3-propanediylbis(oxy)]bis-	644	647	23.93	mainlib	17367-38-3	241948
Octadecane, 3-ethyl-5-(2-ethylbutyl)-	619	650	7.3	replib	55282-12-7	2669
Stearic acid, 3-(octadecyloxy)propyl ester	618	625	7.01	mainlib	17367-40-7	42013

Peak @ 9.971 **Area** 1076096.110 **Area %** 1.82

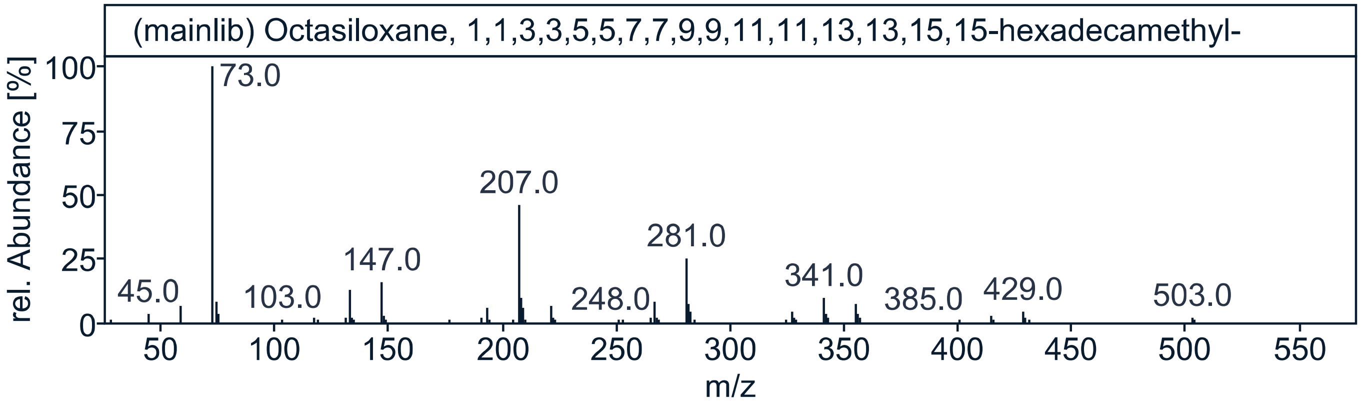

Ion Table

73.0 999 • 207.0 461 • 281.0 250 • 147.0 158 • 133.0 126 • 341.0 97

Compound Name	Score	Rev. Score	Prob. %	Library Name	CAS #	Library Id
Octasiloxane, 1,1,3,3,5,5,7,7,9,9,11,11 ,13,13,15,15-hexadec	706	791	43.06	mainlib	19095-24-0	47312

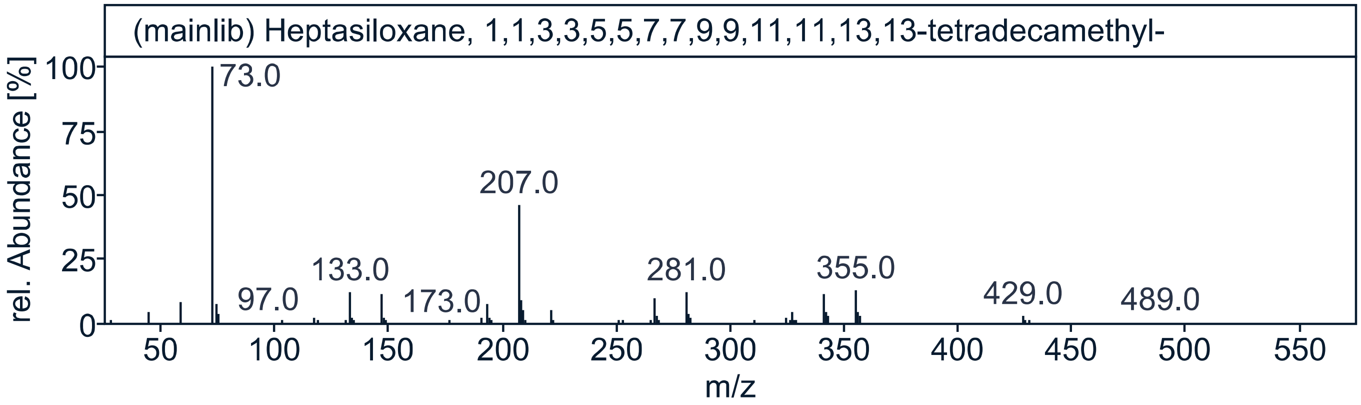

Ion Table

73.0 999 • 207.0 459 • 355.0 128 • 133.0 117 • 281.0 116 • 147.0 113

Compound Name	Score	Rev. Score	Prob. %	Library Name	CAS #	Library Id
Heptasiloxane, 1,1,3,3,5,5,7,7,9,9,11,11 ,13,13-tetradecamet	691	802	26.09	mainlib	19095-23-9	47313

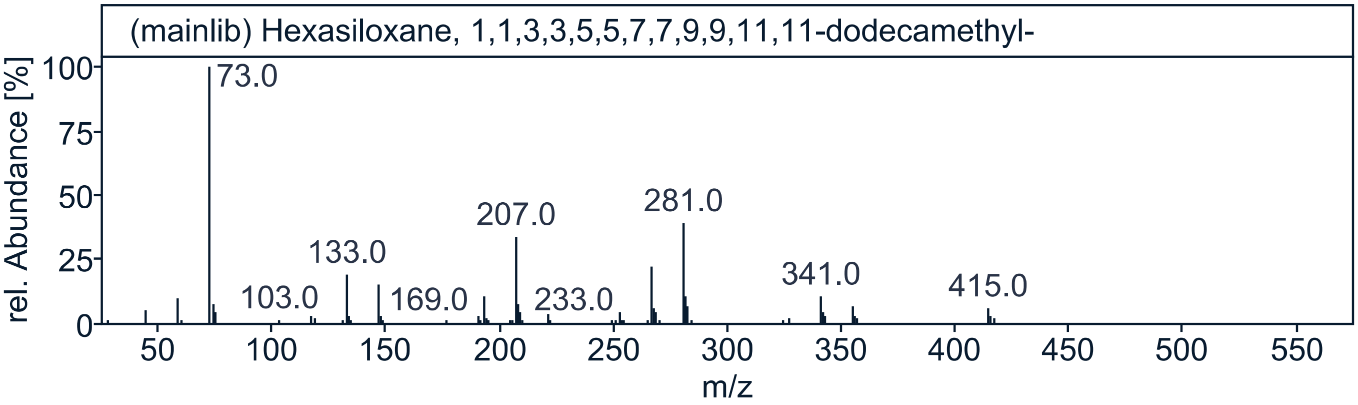

Ion Table

73.0 999 • 281.0 389 • 207.0 340 • 267.0 218 • 133.0 190 • 147.0 150

Compound Name	Score	Rev. Score	Prob. %	Library Name	CAS #	Library Id
Hexasiloxane, 1,1,3,3,5,5,7,7,9,9,11,11-dodecamethyl-	682	781	18.94	mainlib	995-82-4	47869

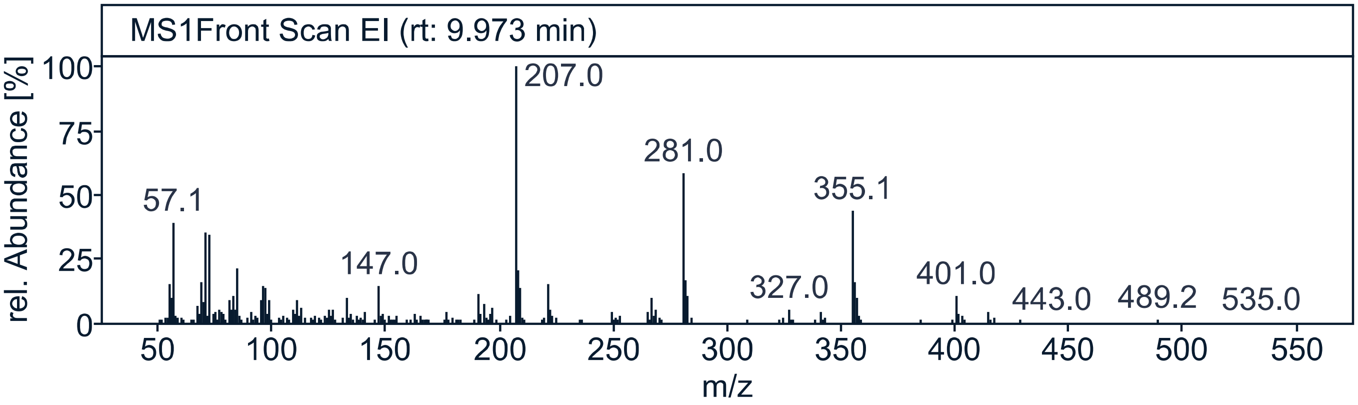

Ion Table

207.0 999 • 281.0 584 • 355.1 435 • 57.1 388 • 71.1 354 • 73.0 343

Summary Hit Table

Compound Name	Score	Rev. Score	Prob. %	Library Name	CAS #	Library Id
Octasiloxane, 1,1,3,3,5,5,7,7,9,9,11,11,13,13,15,15-hexadec	706	791	43.06	mainlib	19095-24-0	47312
Heptasiloxane, 1,1,3,3,5,5,7,7,9,9,11,11,13,13-tetradecamet	691	802	26.09	mainlib	19095-23-9	47313
Hexasiloxane, 1,1,3,3,5,5,7,7,9,9,11,11-dodecamethyl-	682	781	18.94	mainlib	995-82-4	47869

**Peak @**	10.246	**Area**	1991845.936	**Area %**	3.36

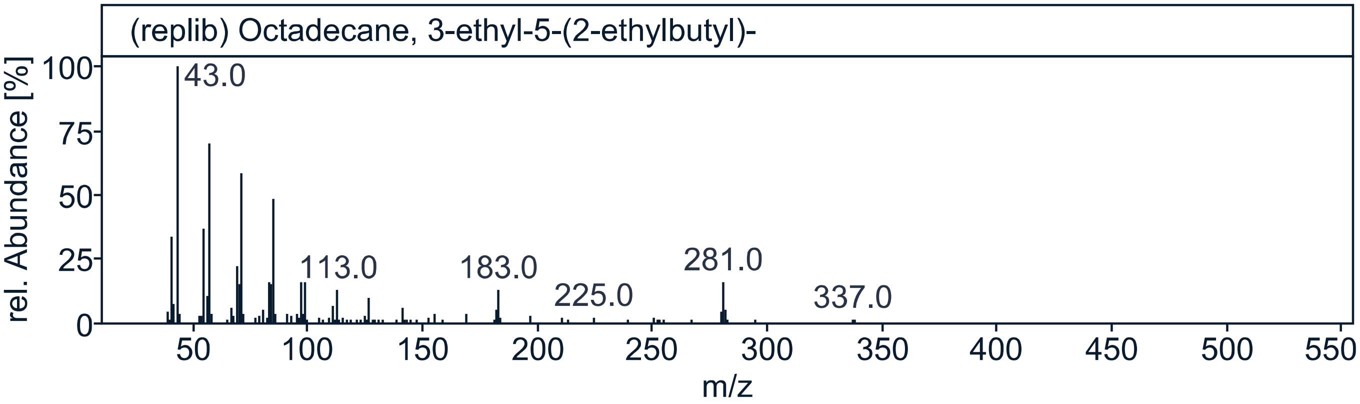

Ion Table

43.0 999 • 57.0 700 • 71.0 582 • 85.0 484 • 55.0 370 • 41.0 336

Compound Name	Score	Rev. Score	Prob. %	Library Name	CAS #	Library Id
Octadecane, 3-ethyl-5-(2-ethylbutyl)-	663	687	12.98	replib	55282-12-7	2669

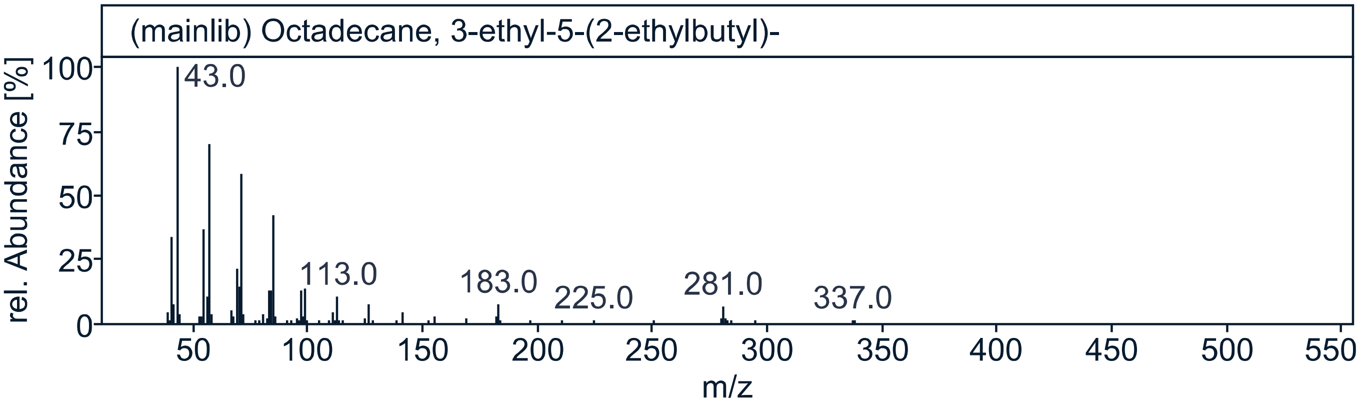

Ion Table

43.0 999 • 57.0 701 • 71.0 583 • 85.0 418 • 55.0 371 • 41.0 337

Compound Name	Score	Rev. Score	Prob. %	Library Name	CAS #	Library Id
Octadecane, 3-ethyl-5-(2-ethylbutyl)-	662	675	12.98	mainlib	55282-12-7	8437

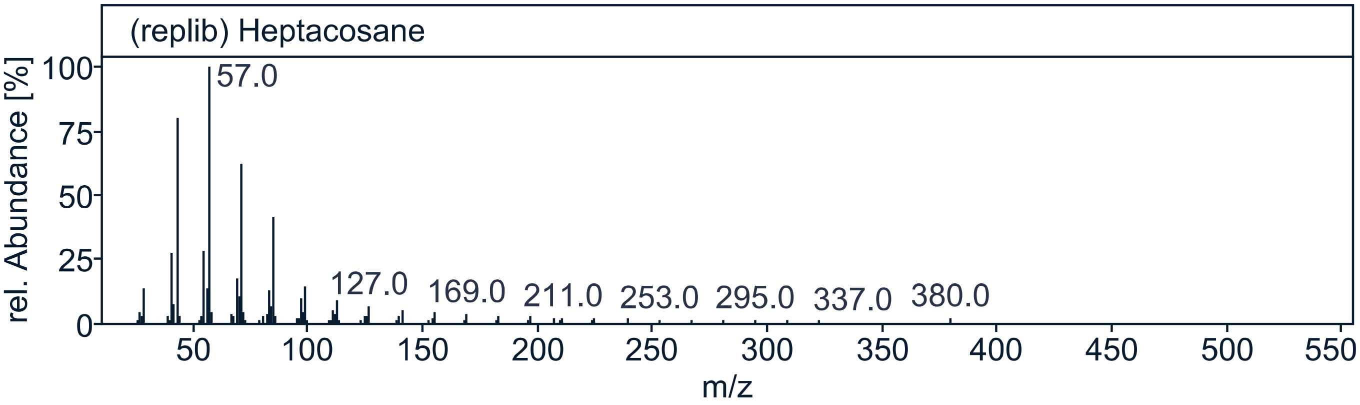

Ion Table

57.0 999 • 43.0 798 • 71.0 622 • 85.0 416 • 55.0 283 • 41.0 275

Compound Name	Score	Rev. Score	Prob. %	Library Name	CAS #	Library Id
Heptacosane	659	733	10.96	replib	593-49-7	7039

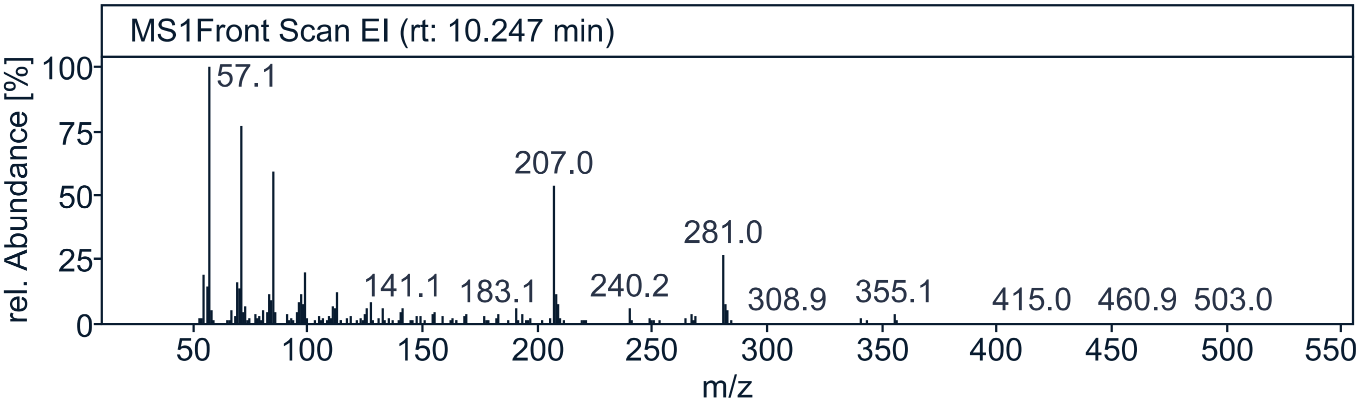

Ion Table

57.1 999 • 71.1 773 • 85.1 593 • 207.0 535 • 281.0 267 • 99.1 196

Summary Hit Table

Compound Name	Score	Rev. Score	Prob. %	Library Name	CAS #	Library Id
Octadecane, 3-ethyl-5-(2-ethylbutyl)-	663	687	12.98	replib	55282-12-7	2669
Octadecane, 3-ethyl-5-(2-ethylbutyl)-	662	675	12.98	mainlib	55282-12-7	8437
Heptacosane	659	733	10.96	replib	593-49-7	7039

**Peak @**	12.963	**Area**	1441568.271	**Area %**	2.43

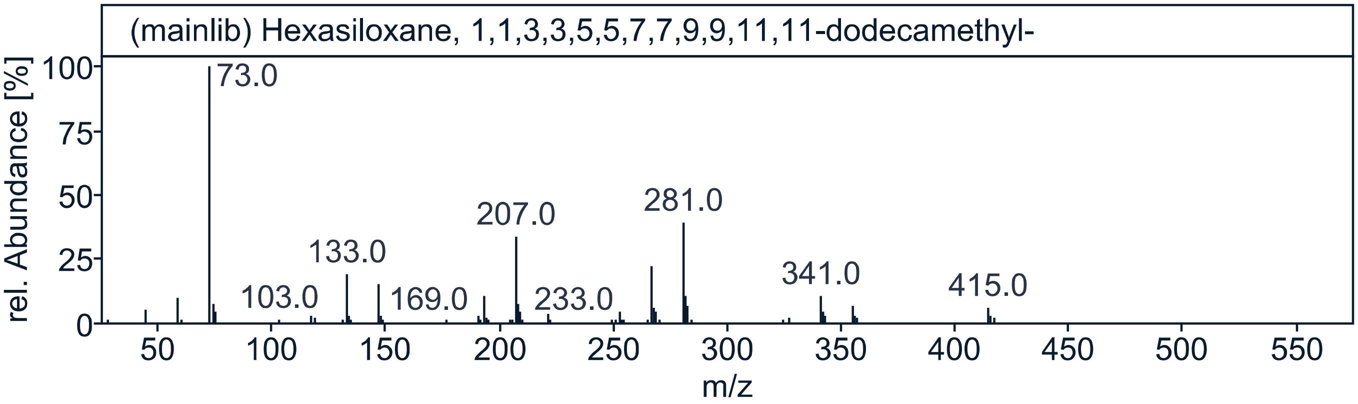

Ion Table

73.0 999 • 281.0 389 • 207.0 340 • 267.0 218 • 133.0 190 • 147.0 150

Compound Name	Score	Rev. Score	Prob. %	Library Name	CAS #	Library Id
Hexasiloxane, 1,1,3,3,5,5,7,7,9,9,11,11-dodecamethyl-	661	753	40.67	mainlib	995-82-4	47869

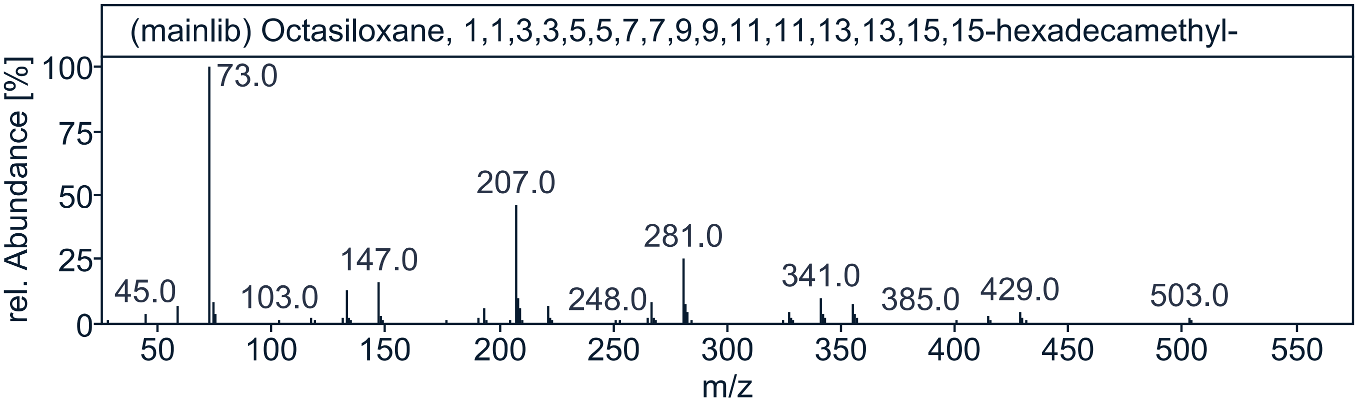

Ion Table

73.0 999 • 207.0 461 • 281.0 250 • 147.0 158 • 133.0 126 • 341.0 97

Compound Name	Score	Rev. Score	Prob. %	Library Name	CAS #	Library Id
Octasiloxane, 1,1,3,3,5,5,7,7,9,9,11,11,13,13,15,15-hexadec	639	734	16.07	mainlib	19095-24-0	47312

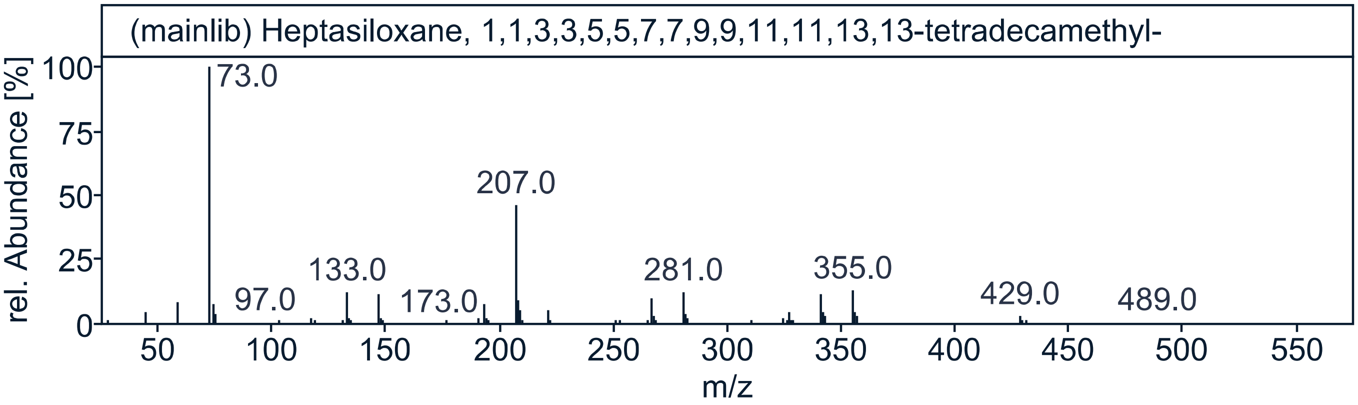

Ion Table

73.0 999 • 207.0 459 • 355.0 128 • 133.0 117 • 281.0 116 • 147.0 113

Compound Name	Score	Rev. Score	Prob. %	Library Name	CAS #	Library Id
Heptasiloxane, 1,1,3,3,5,5,7,7,9,9,11,11 ,13,13-tetradecamet	633	740	12.63	mainlib	19095-23-9	47313

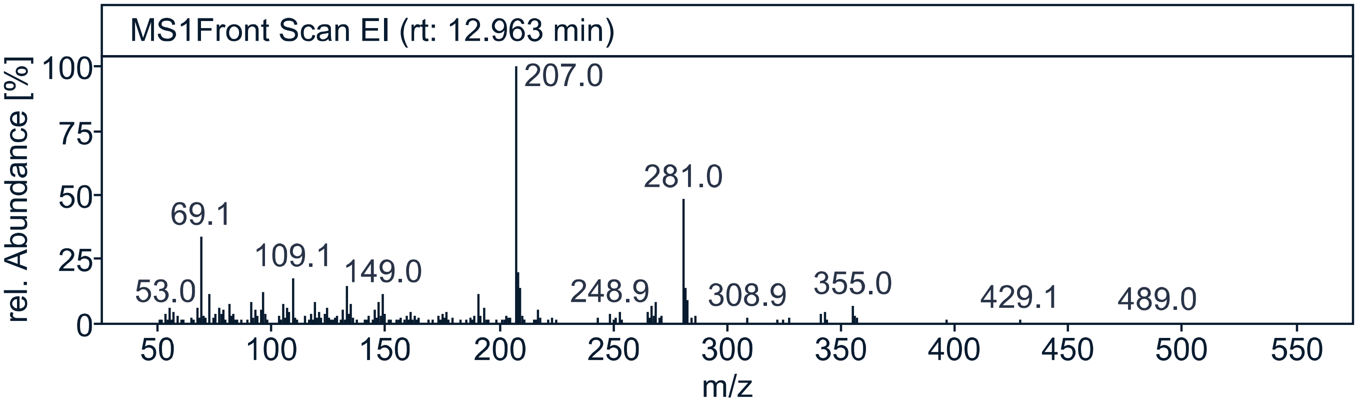

Ion Table

207.0 999 • 281.0 485 • 69.1 336 • 208.0 200 • 109.1 175 • 133.0 140

Summary Hit Table

Compound Name	Score	Rev. Score	Prob. %	Library Name	CAS #	Library Id
Hexasiloxane, 1,1,3,3,5,5,7,7,9,9,11,11-dodecamethyl-	661	753	40.67	mainlib	995-82-4	47869
Octasiloxane, 1,1,3,3,5,5,7,7,9,9,11,11 ,13,13,15,15-hexadec	639	734	16.07	mainlib	19095-24-0	47312
Heptasiloxane, 1,1,3,3,5,5,7,7,9,9,11,11 ,13,13-tetradecamet	633	740	12.63	mainlib	19095-23-9	47313

Peak @ 13.295 **Area** 914183.256 **Area %** 1.54

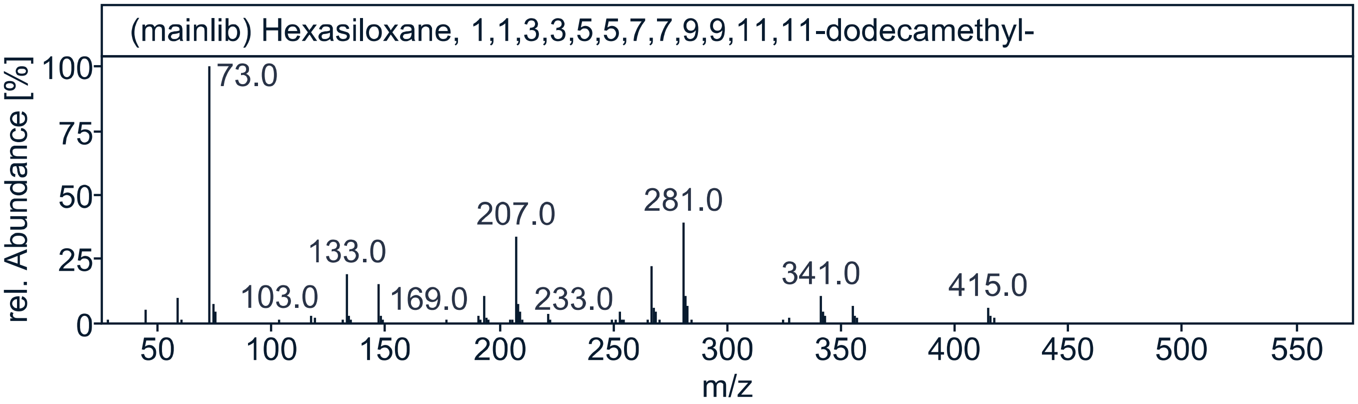

Ion Table

73.0 999 • 281.0 389 • 207.0 340 • 267.0 218 • 133.0 190 • 147.0 150

Compound Name	Score	Rev. Score	Prob. %	Library Name	CAS #	Library Id
Hexasiloxane, 1,1,3,3,5,5,7,7,9,9,11,11-dodecamethyl-	683	762	43.07	mainlib	995-82-4	47869

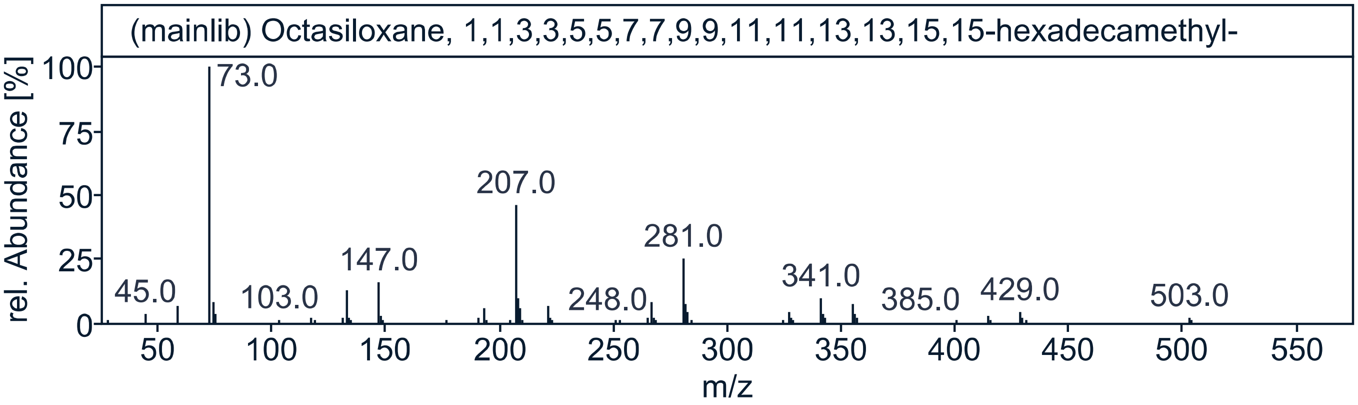

Ion Table

73.0 999 • 207.0 461 • 281.0 250 • 147.0 158 • 133.0 126 • 341.0 97

Compound Name	Score	Rev. Score	Prob. %	Library Name	CAS #	Library Id
Octasiloxane, 1,1,3,3,5,5,7,7,9,9,11,11,13,13,15,15-hexadec	669	749	26.96	mainlib	19095-24-0	47312

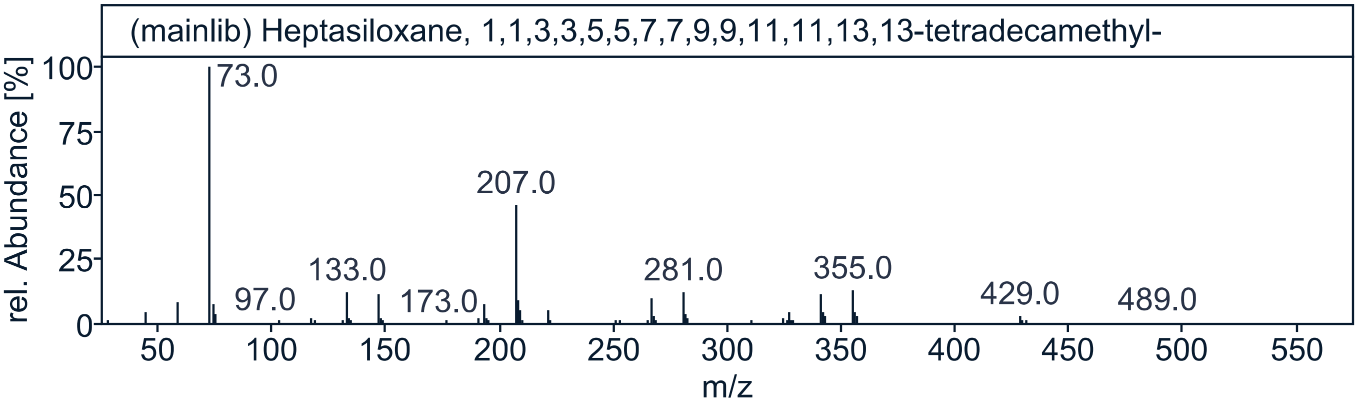

Ion Table

73.0 999 • 207.0 459 • 355.0 128 • 133.0 117 • 281.0 116 • 147.0 113

Compound Name	Score	Rev. Score	Prob. %	Library Name	CAS #	Library Id
Heptasiloxane, 1,1,3,3,5,5,7,7,9,9,11,11 ,13,13-tetradecamet	652	745	14.71	mainlib	19095-23-9	47313

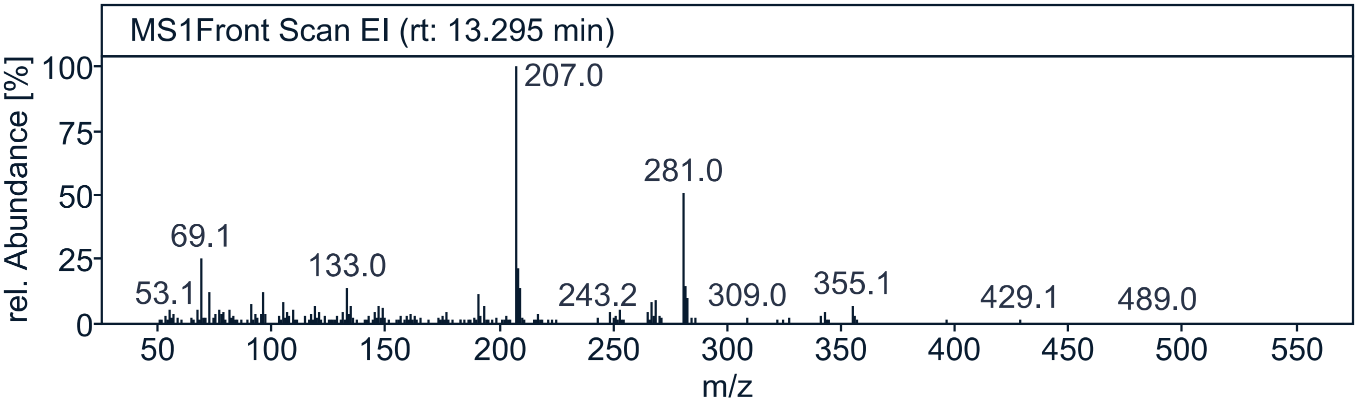

Ion Table

207.0 999 • 281.0 504 • 69.1 252 • 208.0 212 • 282.0 146 • 209.0 139

Summary Hit Table

Compound Name	Score	Rev. Score	Prob. %	Library Name	CAS #	Library Id
Hexasiloxane, 1,1,3,3,5,5,7,7,9,9,11,11-dodecamethyl-	683	762	43.07	mainlib	995-82-4	47869
Octasiloxane, 1,1,3,3,5,5,7,7,9,9,11,11 ,13,13,15,15-hexadec	669	749	26.96	mainlib	19095-24-0	47312
Heptasiloxane, 1,1,3,3,5,5,7,7,9,9,11,11 ,13,13-tetradecamet	652	745	14.71	mainlib	19095-23-9	47313
