# Supplementary material for: Comparative Antioxidant Evaluation and GC-MS Identification of Bioactive Constituents in Litsea cubeba (Lour.) Pers. Fractions
Source: Molecules. 2026 Apr 30;31(9):1506. doi: 10.3390/molecules31091506 (PMC13164878; doi:10.3390/molecules31091506)
Supplement: Supplementary file 1 [file molecules-31-01506-s001.zip › Supplementary File. S2.pdf]

**Data file:**	/TO2021/CL/Results/20250401 XCY 01 BS2021001.rslt\02 Sample 1.dx		
**Sample name:**	Sample 1		
**Description:**			
**Sample amount:**	0.000	**Sample type:**	Sample
**Instrument:**	BS2021001	**Location:**	102
**Injection date:**	2025-04-01 13:22:07+08:00	**Injection:**	1 of 1
**Acq. method:**	RS-GC-MS(Front) 02.amx	**Injection volume:**	0.010
**Analysis method:**	*20250401 XCY 01 PM 01.pmx	**Acq. operator:**	chunyuan xia
**Last changed:**	2025-04-01 14:03:32+08:00		

**Data Analysis Method:**	**20250401 XCY 01 PM 01.pmx**
**Path:**	**/TO2021/CL/Results/20250401 XCY 01 BS2021001.rslt**

Method Parameters

MS Library Search Parameters

Automatically search TIC peaks:	Yes
MS Library:	C:\NIST17\MSSEARCH\mainlib
Maximum number of hits returned:	5
Minimum spectrum match score:	600

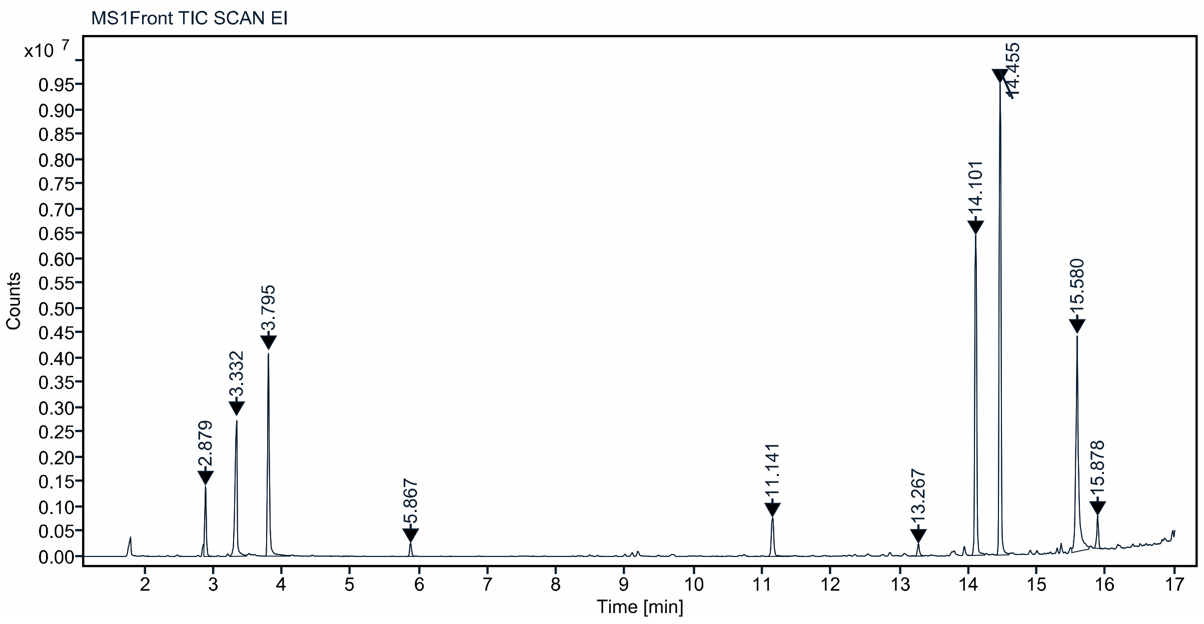

Peak @ 2.879 **Area** 2458868.171 **Area %** 3.98

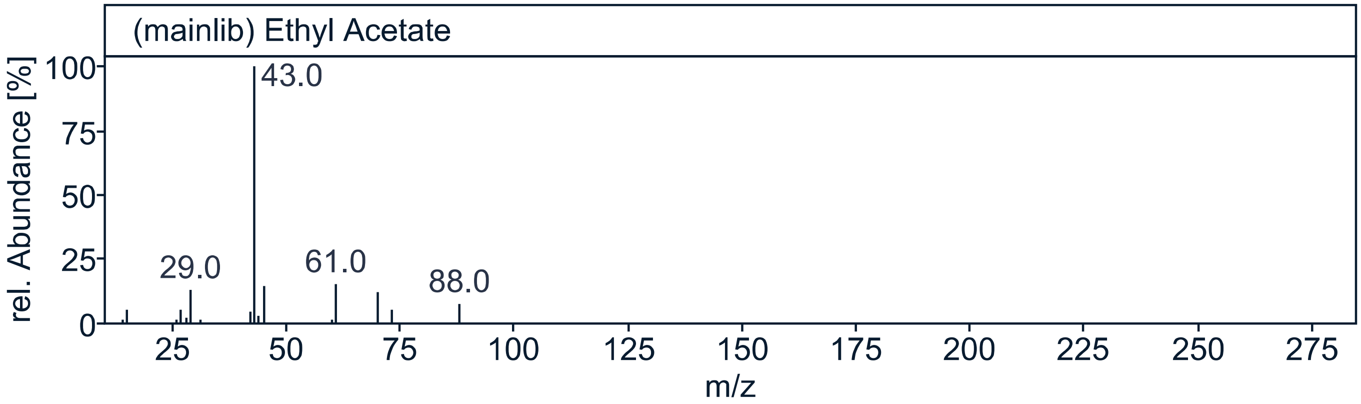

Ion Table

43.0 999 • 61.0 153 • 45.0 146 • 29.0 124 • 70.0 118 • 88.0 70

Compound Name	Score	Rev. Score	Prob. %	Library Name	CAS #	Library Id
Ethyl Acetate	913	913	98.18	mainlib	141-78-6	8863

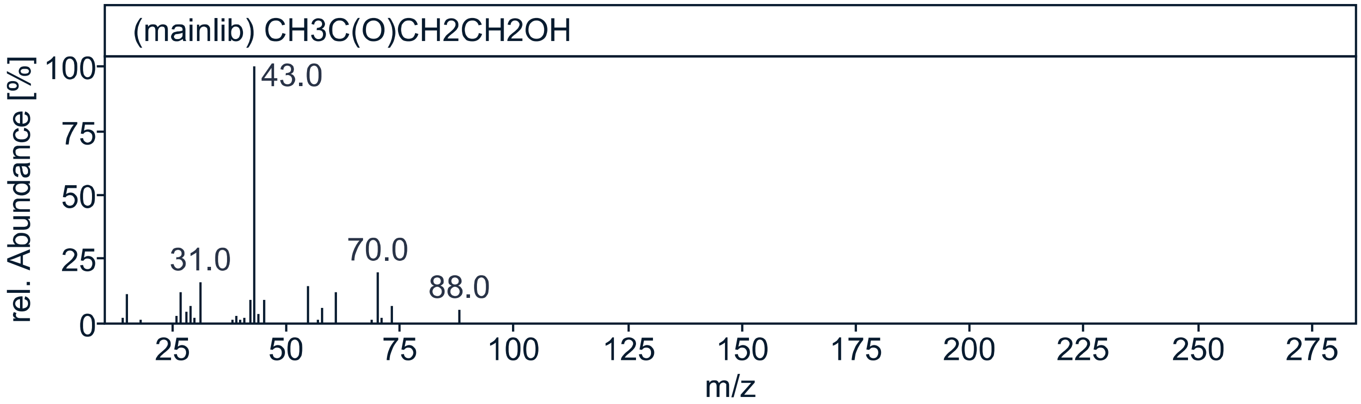

Ion Table

43.0 999 • 70.0 198 • 31.0 155 • 55.0 144 • 61.0 120 • 27.0 116

Compound Name	Score	Rev. Score	Prob. %	Library Name	CAS #	Library Id
CH3C(O)CH2CH2OH	718	719	1.37	mainlib	590-90-9	9217

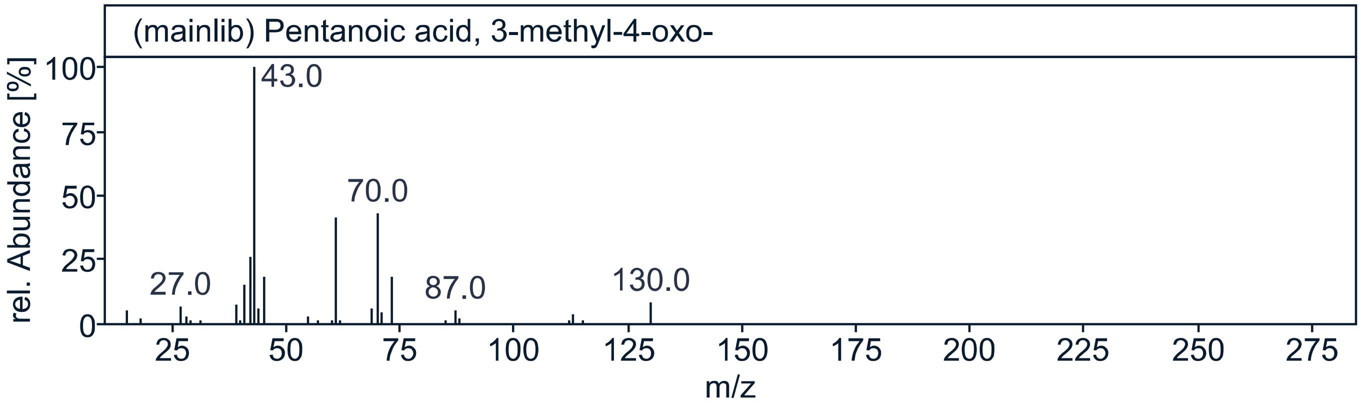

Ion Table

43.0 999 • 70.0 427 • 61.0 411 • 42.0 261 • 73.0 183 • 45.0 179

Compound Name	Score	Rev. Score	Prob. %	Library Name	CAS #	Library Id
Pentanoic acid, 3-methyl-4-oxo-	653	653	0.22	mainlib	6628-79-1	9266

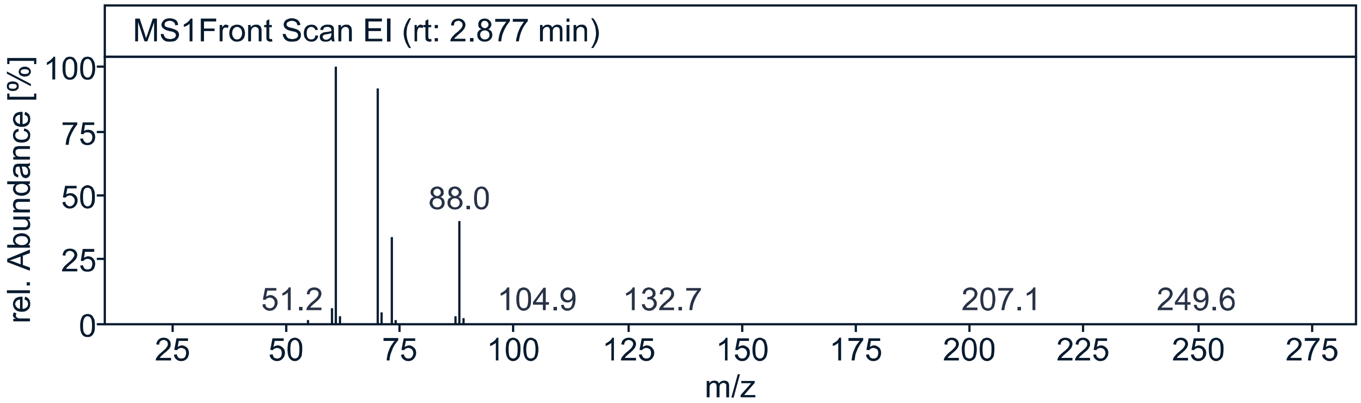

Ion Table

61.0 999 • 70.0 919 • 88.0 394 • 73.0 338 • 60.0 58 • 71.0 44

Summary Hit Table

Compound Name	Score	Rev. Score	Prob. %	Library Name	CAS #	Library Id
Ethyl Acetate	913	913	98.18	mainlib	141-78-6	8863
CH3C(O)CH2CH2OH	718	719	1.37	mainlib	590-90-9	9217
Pentanoic acid, 3-methyl-4-oxo-	653	653	0.22	mainlib	6628-79-1	9266

**Peak @**	3.332	**Area**	6606504.773	**Area %**	10.69

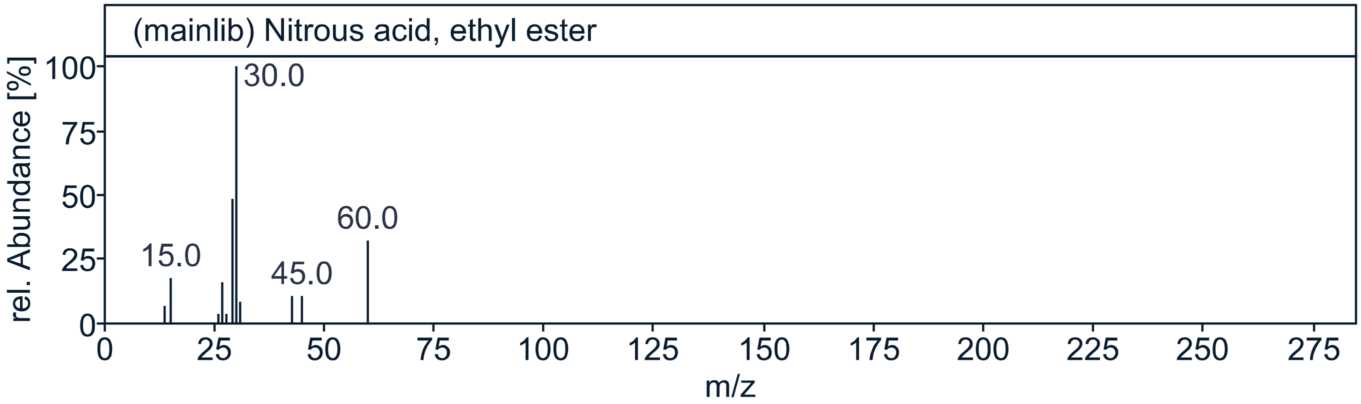

Ion Table

30.0 999 • 29.0 481 • 60.0 323 • 15.0 174 • 27.0 158 • 45.0 106

Compound Name	Score	Rev. Score	Prob. %	Library Name	CAS #	Library Id
Nitrous acid, ethyl ester	957	999	23.41	mainlib	109-95-5	995

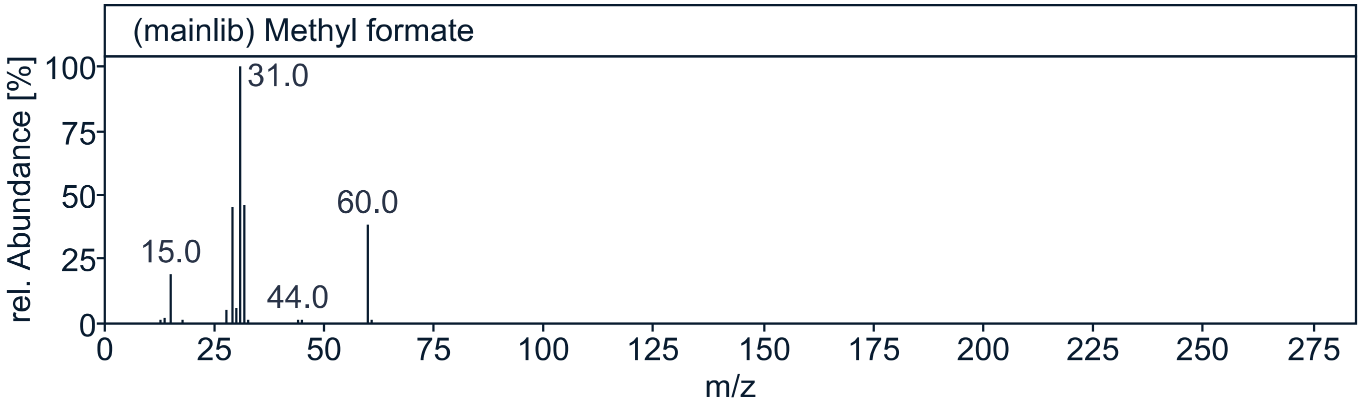

Ion Table

31.0 999 • 32.0 457 • 29.0 454 • 60.0 379 • 15.0 187 • 30.0 60

Compound Name	Score	Rev. Score	Prob. %	Library Name	CAS #	Library Id
Methyl formate	957	963	23.41	mainlib	107-31-3	1710

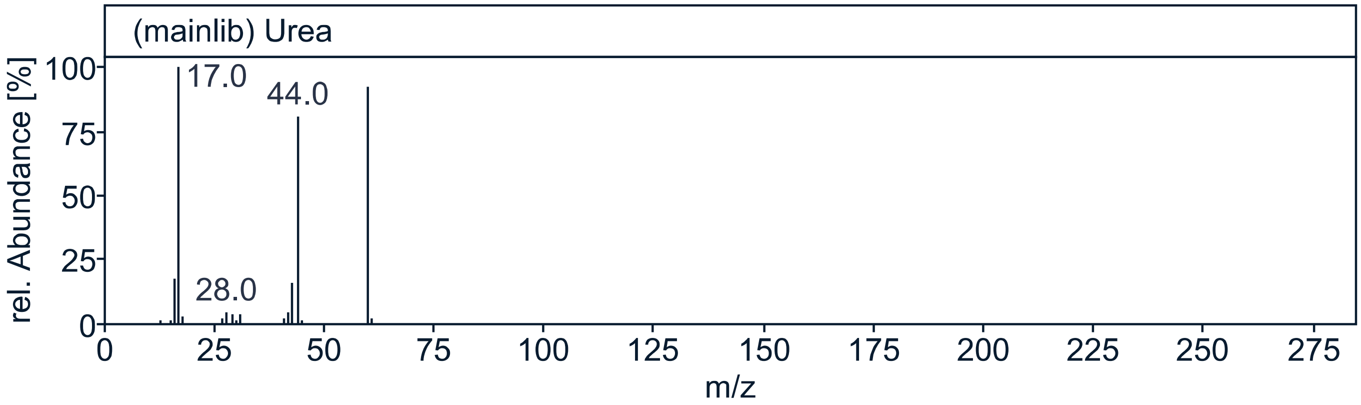

Ion Table

17.0 999 • 60.0 926 • 44.0 807 • 16.0 172 • 43.0 156 • 28.0 42

Compound Name	Score	Rev. Score	Prob. %	Library Name	CAS #	Library Id
Urea	951	958	18.4	mainlib	57-13-6	61

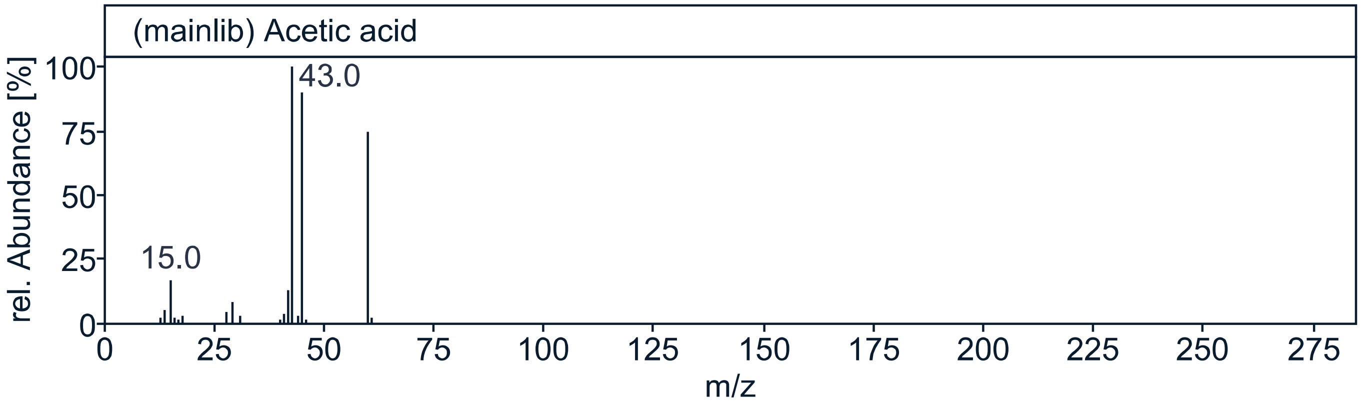

Ion Table

43.0 999 • 45.0 903 • 60.0 747 • 15.0 170 • 42.0 130 • 29.0 84

Compound Name	Score	Rev. Score	Prob. %	Library Name	CAS #	Library Id
Acetic acid	947	951	15.54	mainlib	64-19-7	7393

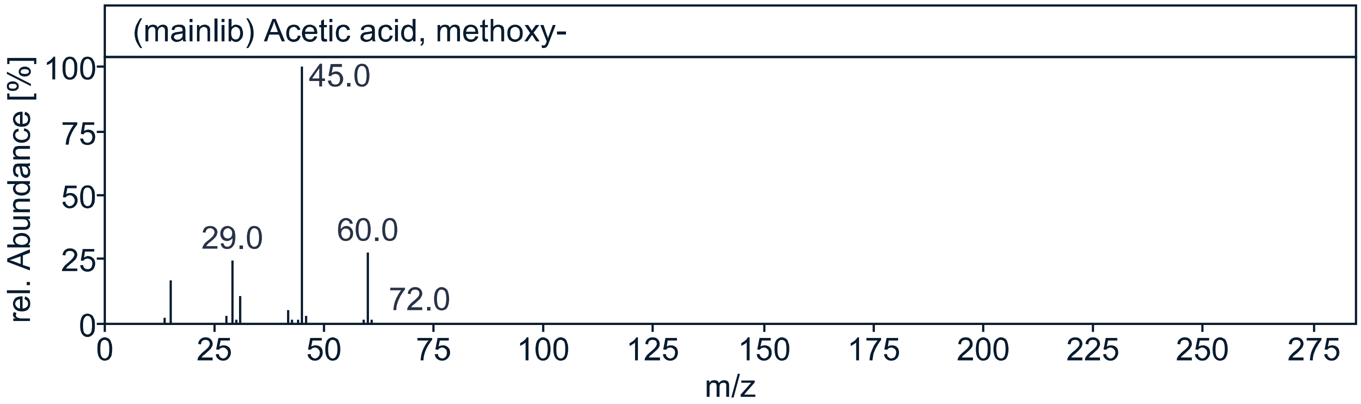

Ion Table

45.0 999 • 60.0 271 • 29.0 244 • 15.0 165 • 31.0 107 • 42.0 54

Compound Name	Score	Rev. Score	Prob. %	Library Name	CAS #	Library Id
Acetic acid, methoxy-	934	939	10.04	mainlib	625-45-6	18611

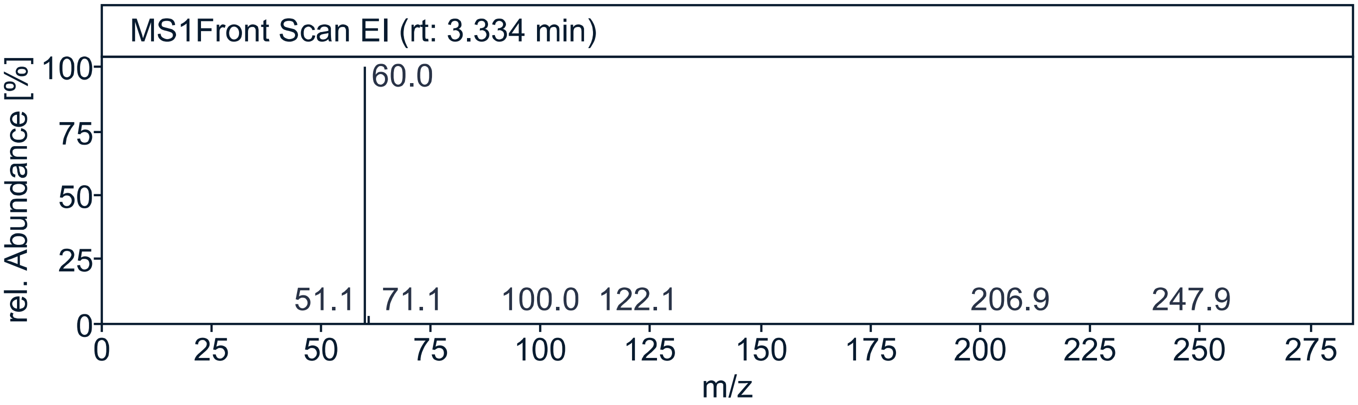

Ion Table

60.0 999 • 61.0 27 • 62.0 5 • 56.0 3 • 57.0 3 • 71.1 3

Summary Hit Table

Compound Name	Score	Rev. Score	Prob. %	Library Name	CAS #	Library Id
Nitrous acid, ethyl ester	957	999	23.41	mainlib	109-95-5	995
Methyl formate	957	963	23.41	mainlib	107-31-3	1710
Urea	951	958	18.4	mainlib	57-13-6	61
Acetic acid	947	951	15.54	mainlib	64-19-7	7393
Acetic acid, methoxy-	934	939	10.04	mainlib	625-45-6	18611

Peak @ 3.795 **Area** 8352079.667 **Area %** 13.51

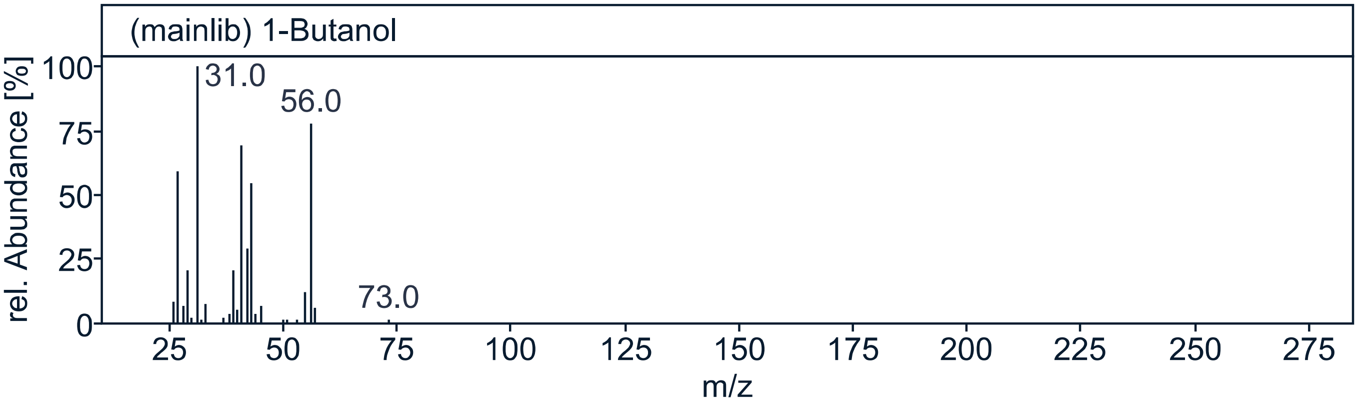

Ion Table

31.0 999 • 56.0 781 • 41.0 694 • 27.0 593 • 43.0 549 • 42.0 292

Compound Name	Score	Rev. Score	Prob. %	Library Name	CAS #	Library Id
1-Butanol	943	949	54.32	mainlib	71-36-3	1764

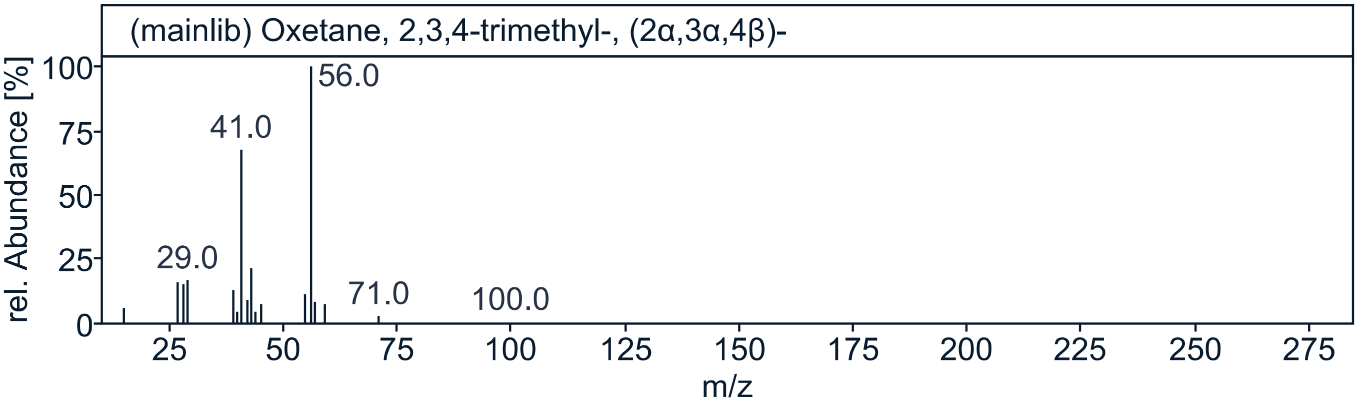

Ion Table

56.0 999 • 41.0 680 • 43.0 210 • 29.0 170 • 27.0 160 • 28.0 150

Compound Name	Score	Rev. Score	Prob. %	Library Name	CAS #	Library Id
Oxetane, 2,3,4-trimethyl-, (2α,3α,4β)-	871	913	7.56	mainlib	32347-12-9	23740

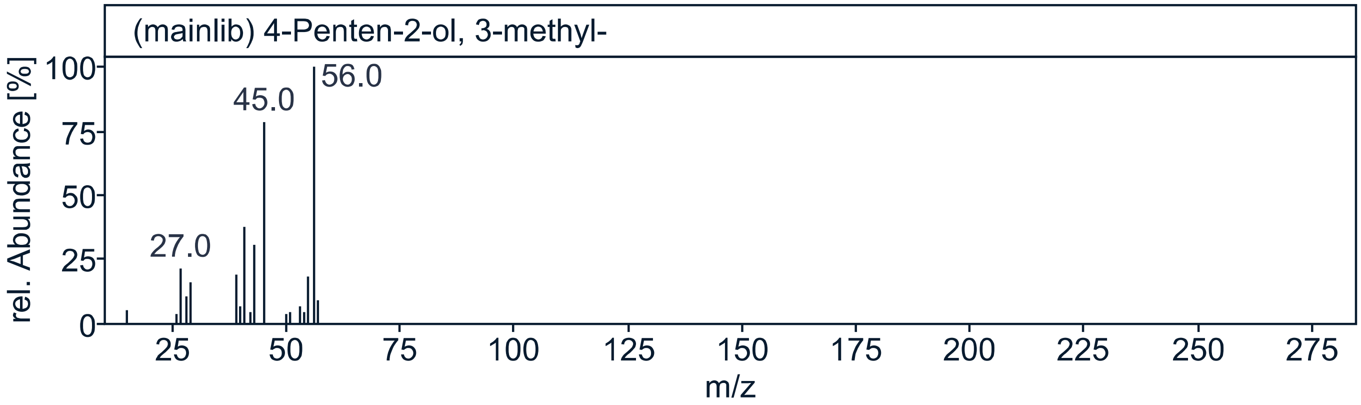

Ion Table

56.0 999 • 45.0 785 • 41.0 375 • 43.0 302 • 27.0 212 • 39.0 186

Compound Name	Score	Rev. Score	Prob. %	Library Name	CAS #	Library Id
4-Penten-2-ol, 3-methyl-	868	896	6.68	mainlib	1569-59-1	24021

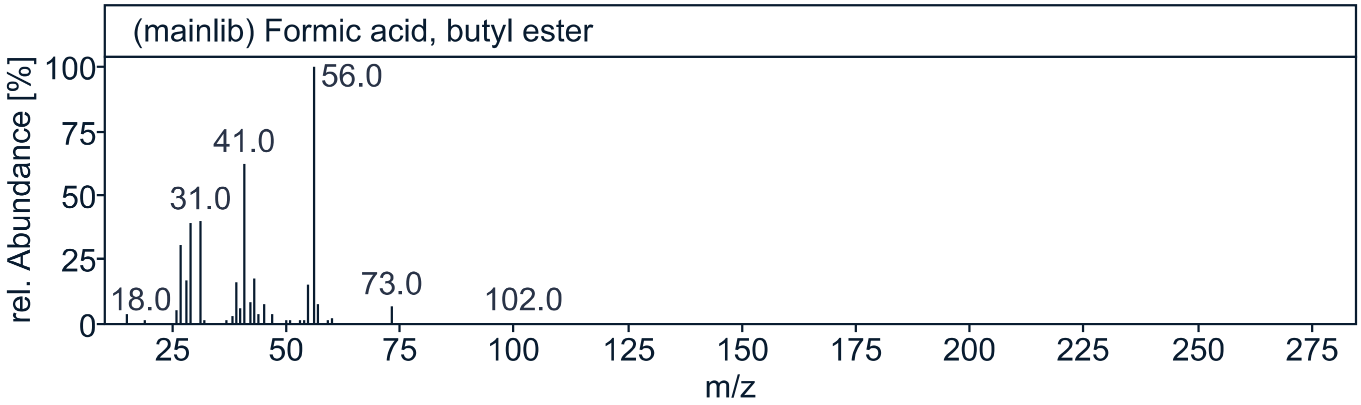

Ion Table

56.0 999 • 41.0 621 • 31.0 400 • 29.0 391 • 27.0 307 • 43.0 176

Compound Name	Score	Rev. Score	Prob. %	Library Name	CAS #	Library Id
Formic acid, butyl ester	865	869	5.9	mainlib	592-84-7	23703

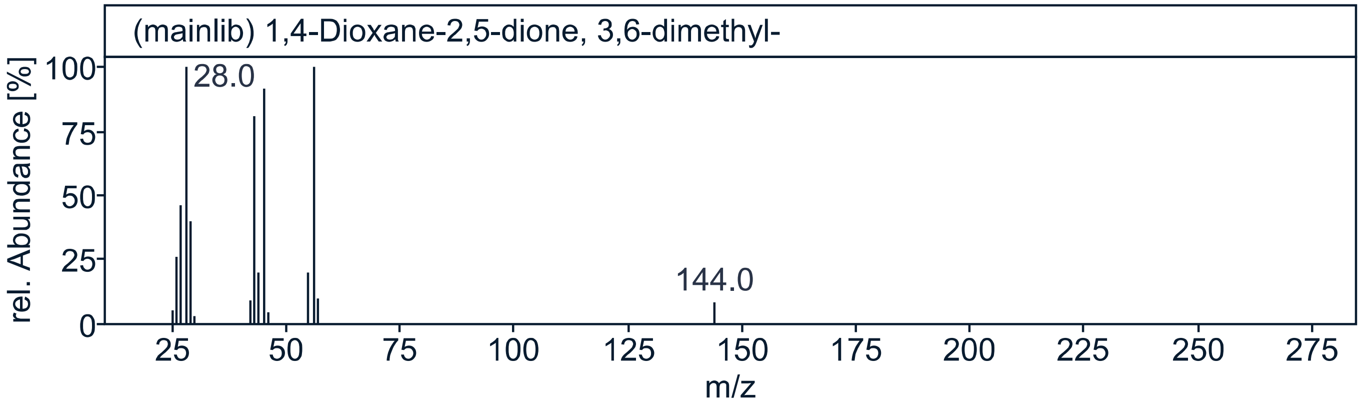

Ion Table

28.0 999 • 56.0 999 • 45.0 920 • 43.0 810 • 27.0 460 • 29.0 400

Compound Name	Score	Rev. Score	Prob. %	Library Name	CAS #	Library Id
1,4-Dioxane-2,5-dione, 3,6-dimethyl-	848	882	3.22	mainlib	95-96-5	250

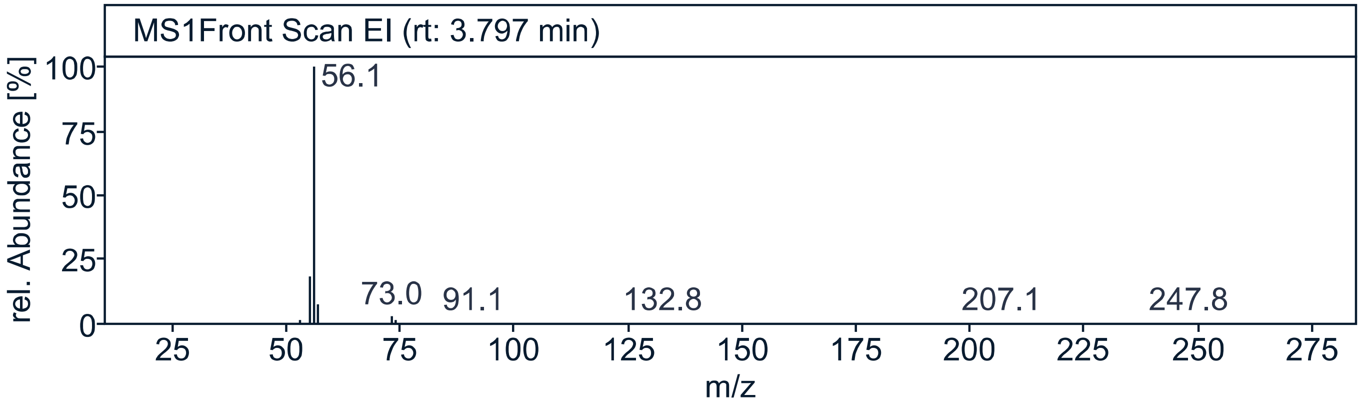

Ion Table

56.1 999 • 55.1 185 • 57.0 75 • 73.0 25 • 53.0 13 • 74.1 9

Summary Hit Table

Compound Name	Score	Rev. Score	Prob. %	Library Name	CAS #	Library Id
1-Butanol	943	949	54.32	mainlib	71-36-3	1764
Oxetane, 2,3,4-trimethyl-, (2α,3α,4β)-	871	913	7.56	mainlib	32347-12-9	23740
4-Penten-2-ol, 3-methyl-	868	896	6.68	mainlib	1569-59-1	24021
Formic acid, butyl ester	865	869	5.9	mainlib	592-84-7	23703
1,4-Dioxane-2,5-dione, 3,6-dimethyl-	848	882	3.22	mainlib	95-96-5	250

Peak @ 5.867 **Area** 477067.190 **Area %** 0.77

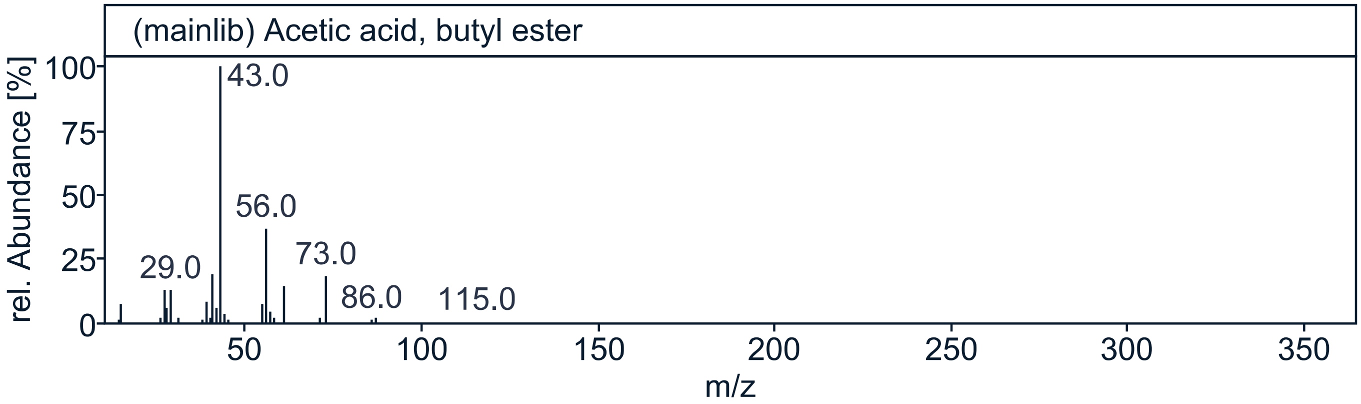

Ion Table

43.0 999 • 56.0 371 • 41.0 189 • 73.0 181 • 61.0 146 • 29.0 129

Compound Name	Score	Rev. Score	Prob. %	Library Name	CAS #	Library Id
Acetic acid, butyl ester	931	951	96.54	mainlib	123-86-4	8067

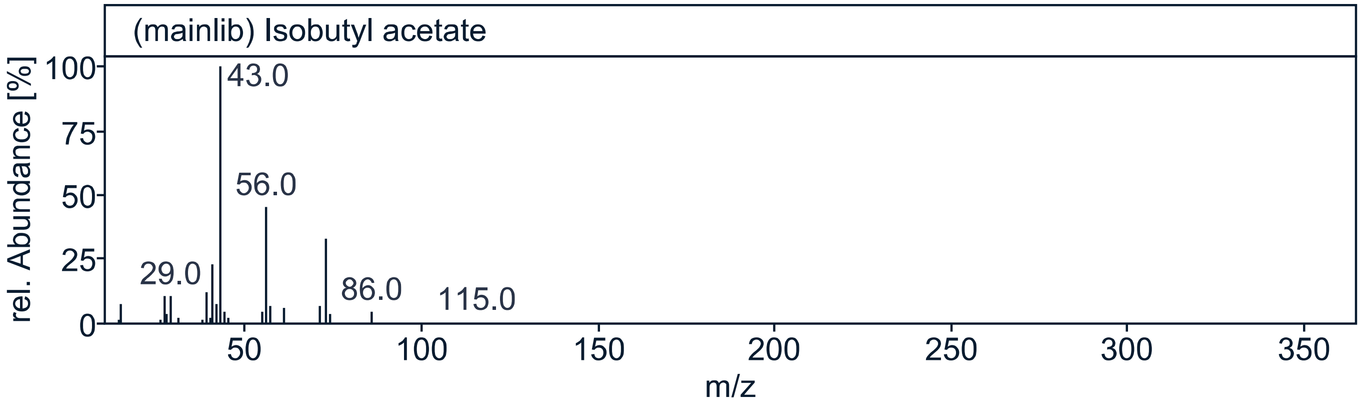

Ion Table

43.0 999 • 56.0 450 • 73.0 332 • 41.0 225 • 39.0 122 • 29.0 104

Compound Name	Score	Rev. Score	Prob. %	Library Name	CAS #	Library Id
Isobutyl acetate	776	800	2.51	mainlib	110-19-0	8100

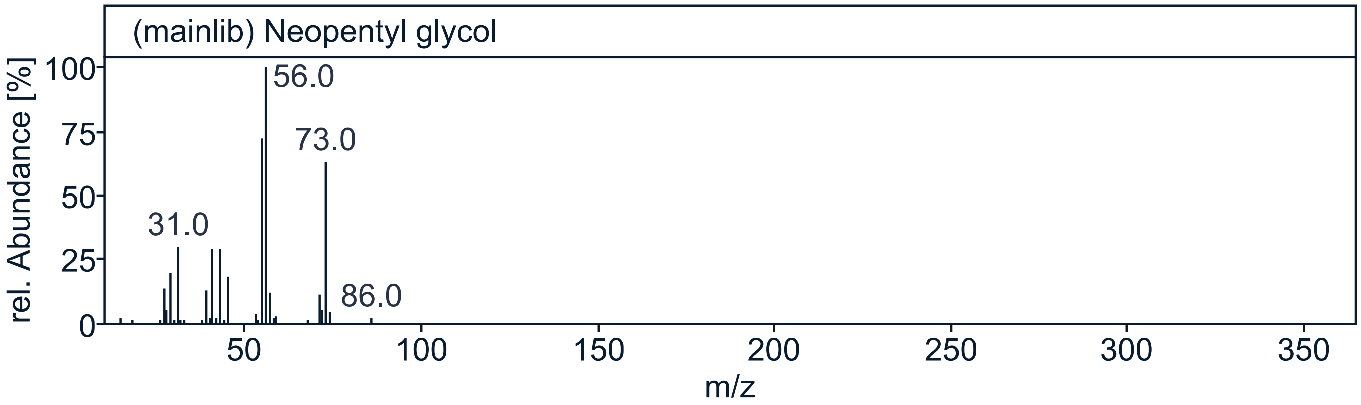

Ion Table

56.0 999 • 55.0 724 • 73.0 633 • 31.0 297 • 41.0 290 • 43.0 290

Compound Name	Score	Rev. Score	Prob. %	Library Name	CAS #	Library Id
Neopentyl glycol	662	689	0.15	mainlib	126-30-7	24100

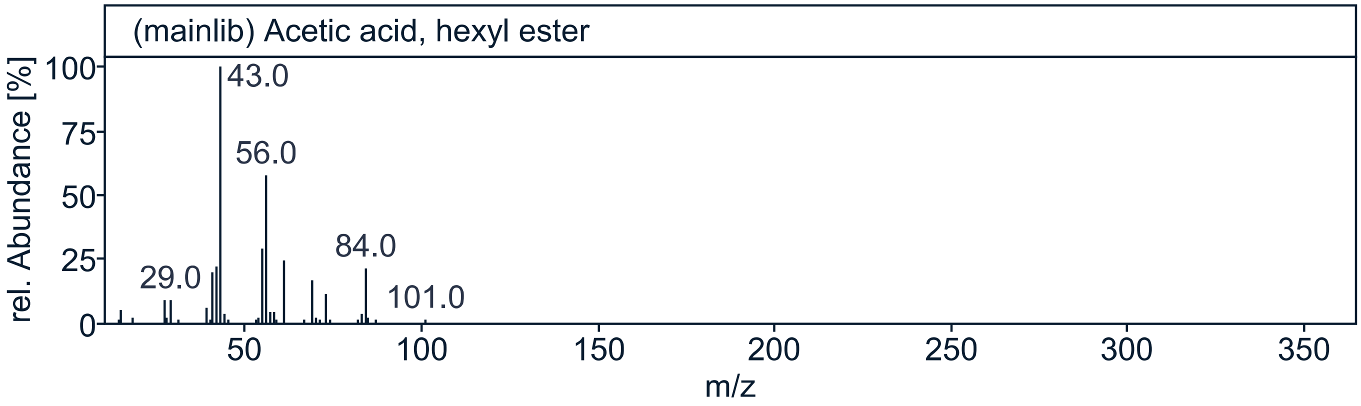

Ion Table

43.0 999 • 56.0 573 • 55.0 292 • 61.0 246 • 42.0 224 • 84.0 214

Compound Name	Score	Rev. Score	Prob. %	Library Name	CAS #	Library Id
Acetic acid, hexyl ester	652	662	0.1	mainlib	142-92-7	8084

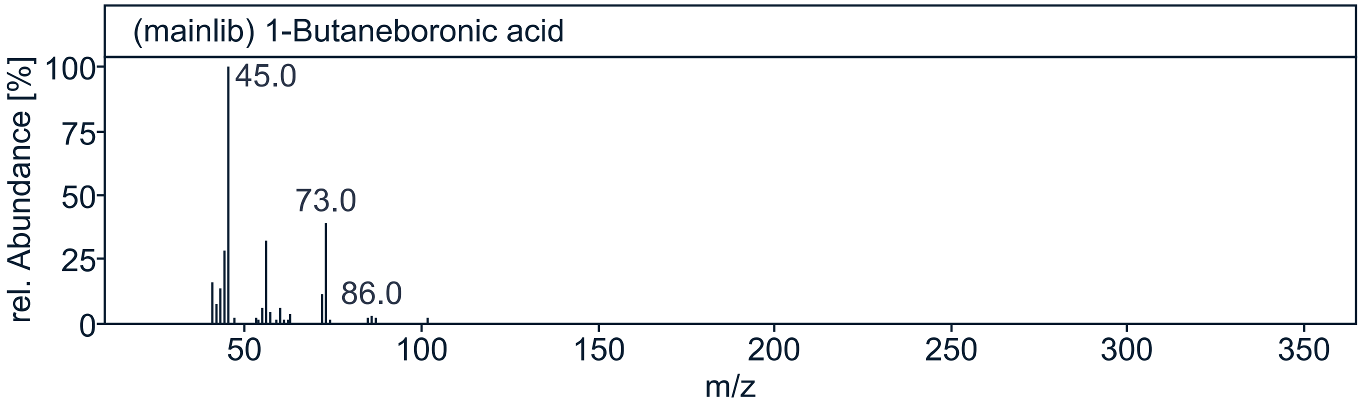

Ion Table

45.0 999 • 73.0 391 • 56.0 318 • 44.0 283 • 41.0 155 • 43.0 132

Compound Name	Score	Rev. Score	Prob. %	Library Name	CAS #	Library Id
1-Butaneboronic acid	650	664	0.09	mainlib	4426-47-5	18734

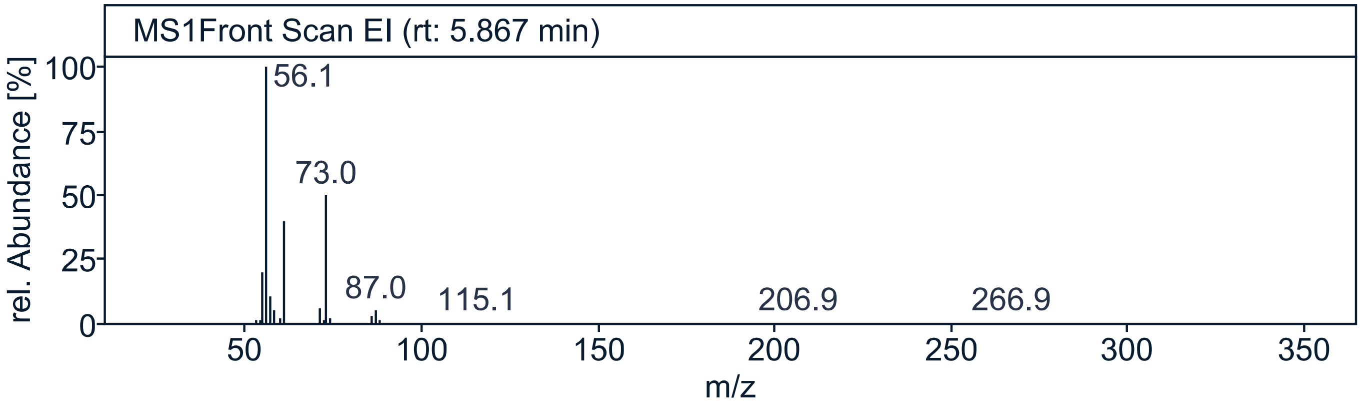

Ion Table

56.1 999 • 73.0 498 • 61.0 399 • 55.0 194 • 57.1 101 • 71.1 56

Summary Hit Table

Compound Name	Score	Rev. Score	Prob. %	Library Name	CAS #	Library Id
Acetic acid, butyl ester	931	951	96.54	mainlib	123-86-4	8067
Isobutyl acetate	776	800	2.51	mainlib	110-19-0	8100
Neopentyl glycol	662	689	0.15	mainlib	126-30-7	24100
Acetic acid, hexyl ester	652	662	0.1	mainlib	142-92-7	8084
1-Butaneboronic acid	650	664	0.09	mainlib	4426-47-5	18734

Peak @ 11.141 **Area** 1937418.765 **Area %** 3.13

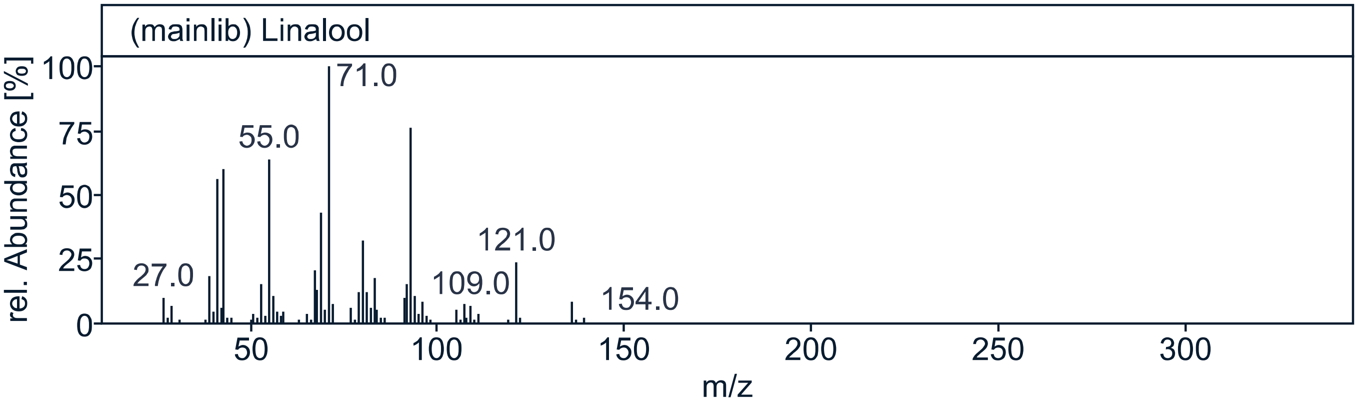

Ion Table

71.0 999 • 93.0 760 • 55.0 637 • 43.0 598 • 41.0 564 • 69.0 426

Compound Name	Score	Rev. Score	Prob. %	Library Name	CAS #	Library Id
Linalool	873	878	26.61	mainlib	78-70-6	42410

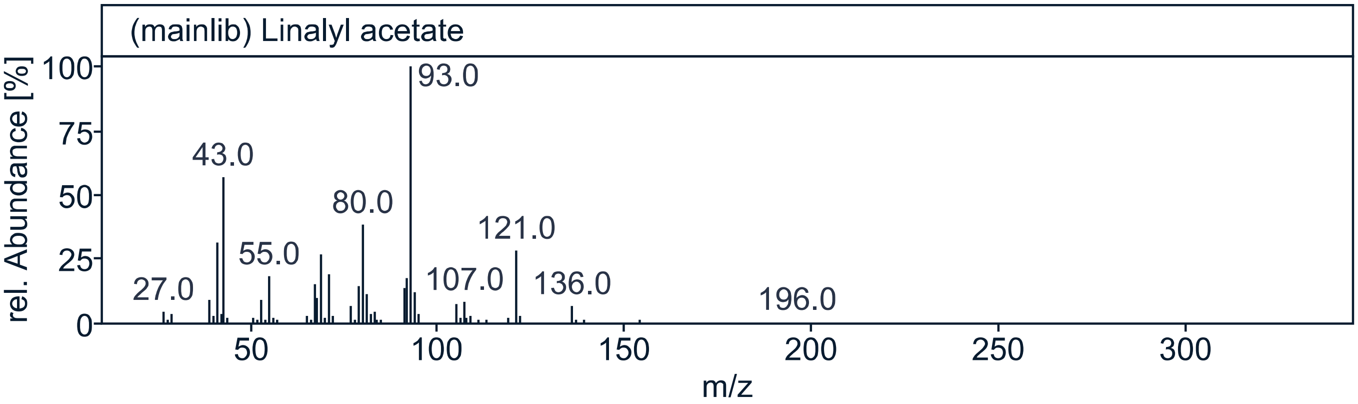

Ion Table

93.0 999 • 43.0 566 • 80.0 386 • 41.0 311 • 121.0 284 • 69.0 263

Compound Name	Score	Rev. Score	Prob. %	Library Name	CAS #	Library Id
Linalyl acetate	871	878	24.54	mainlib	115-95-7	72420

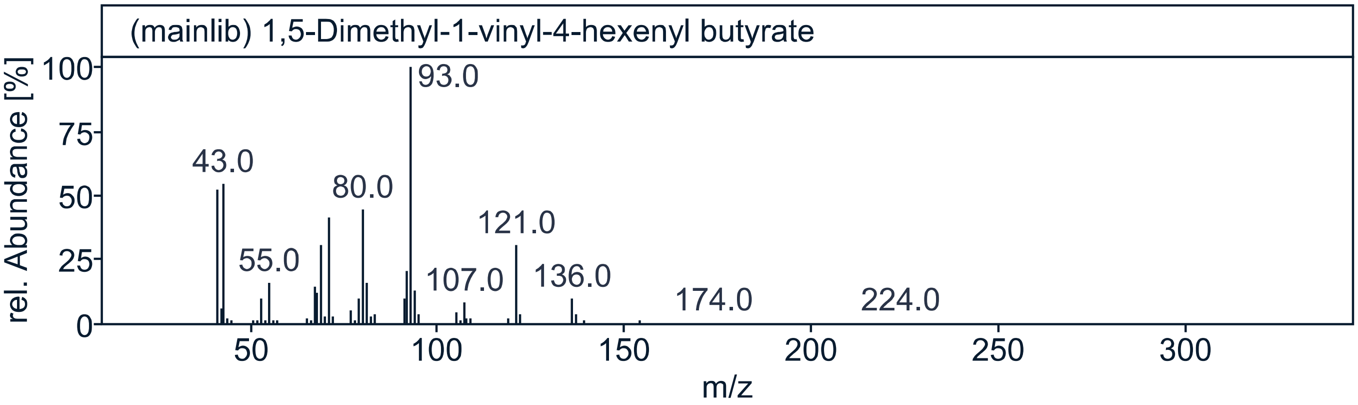

Ion Table

93.0 999 • 43.0 545 • 41.0 522 • 80.0 444 • 71.0 417 • 121.0 309

Compound Name	Score	Rev. Score	Prob. %	Library Name	CAS #	Library Id
1,5-Dimethyl-1-vinyl-4-hexenyl butyrate	853	856	12.65	mainlib	78-36-4	72406

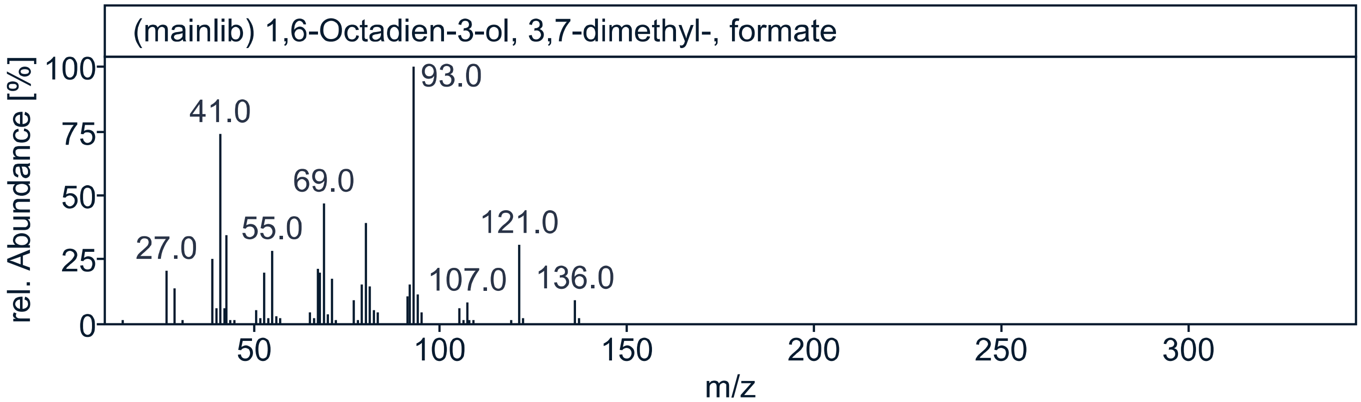

Ion Table

93.0 999 • 41.0 741 • 69.0 471 • 80.0 393 • 43.0 346 • 121.0 304

Compound Name	Score	Rev. Score	Prob. %	Library Name	CAS #	Library Id
1,6-Octadien-3-ol, 3,7-dimethyl-, formate	844	892	9.19	mainlib	115-99-1	72371

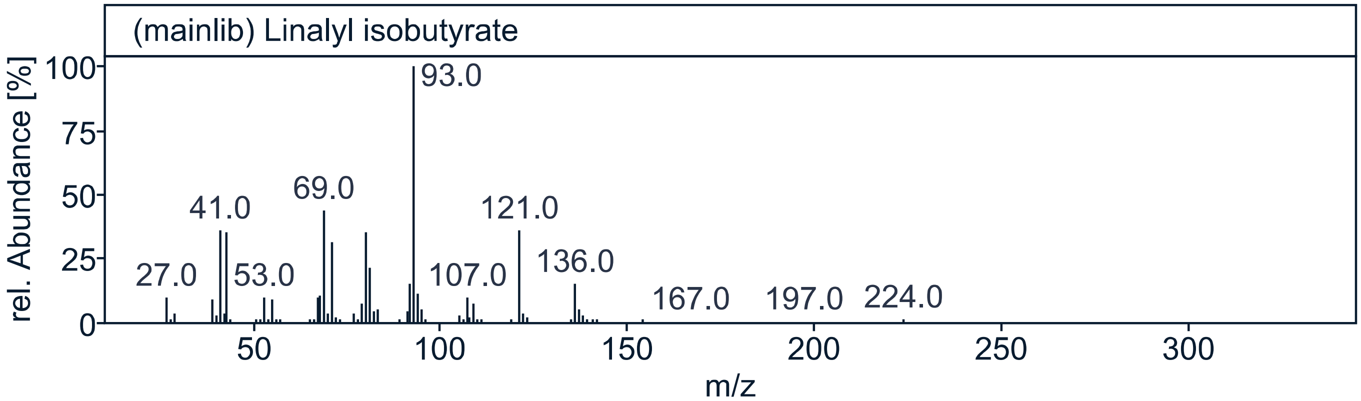

Ion Table

93.0 999 • 69.0 440 • 41.0 360 • 121.0 356 • 80.0 354 • 43.0 350

Compound Name	Score	Rev. Score	Prob. %	Library Name	CAS #	Library Id
Linalyl isobutyrate	818	821	2.75	mainlib	78-35-3	72592

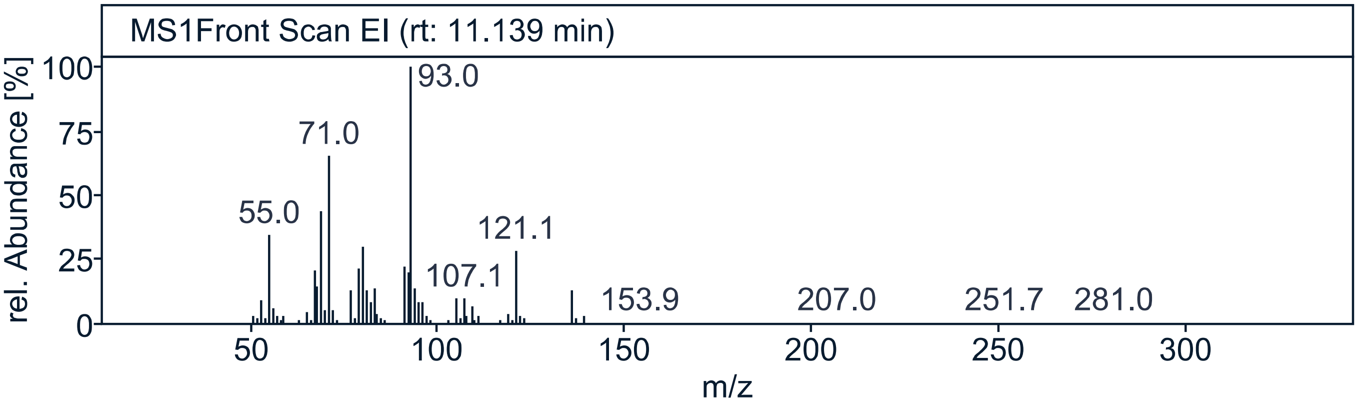

Ion Table

93.0 999 • 71.0 656 • 69.1 441 • 55.0 341 • 80.0 294 • 121.1 281

Summary Hit Table

Compound Name	Score	Rev. Score	Prob. %	Library Name	CAS #	Library Id
Linalool	873	878	26.61	mainlib	78-70-6	42410
Linalyl acetate	871	878	24.54	mainlib	115-95-7	72420
1,5-Dimethyl-1-vinyl-4-hexenyl butyrate	853	856	12.65	mainlib	78-36-4	72406
1,6-Octadien-3-ol, 3,7-dimethyl-, formate	844	892	9.19	mainlib	115-99-1	72371
Linalyl isobutyrate	818	821	2.75	mainlib	78-35-3	72592

Peak @ 13.267 **Area** 568083.829 **Area %** 0.92

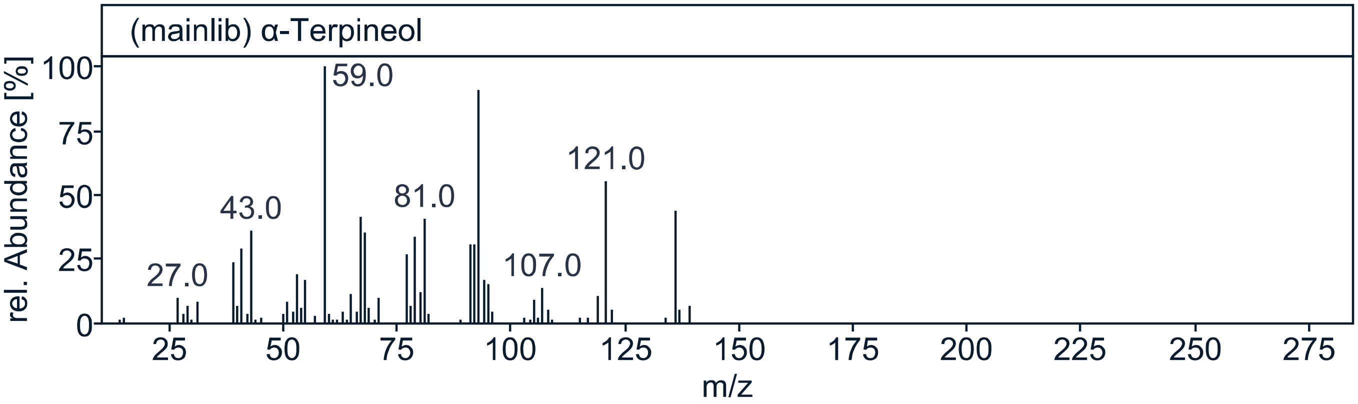

Ion Table

59.0 999 • 93.0 909 • 121.0 555 • 136.0 438 • 67.0 417 • 81.0 408

Compound Name	Score	Rev. Score	Prob. %	Library Name	CAS #	Library Id
α-Terpineol	889	930	44.45	mainlib	98-55-5	32871

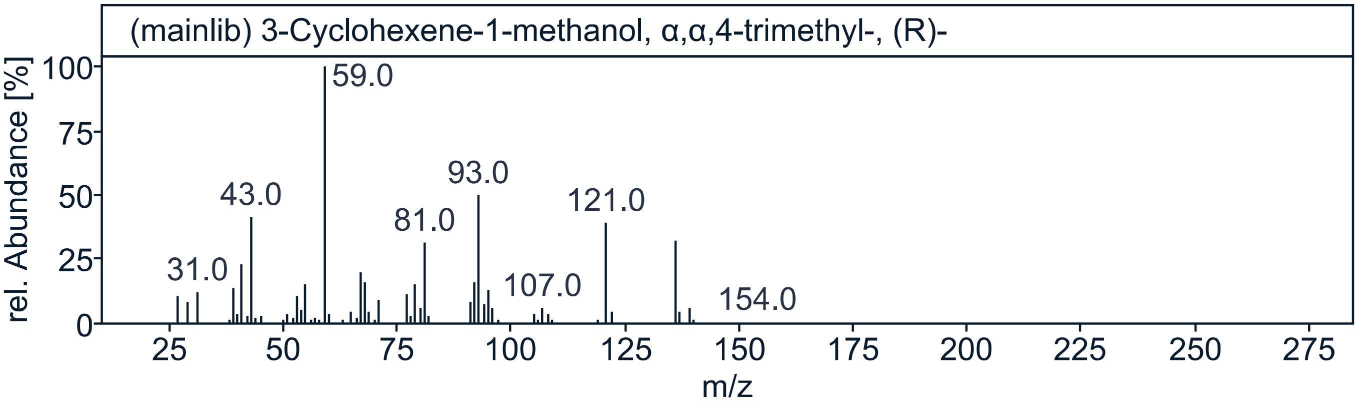

Ion Table

59.0 999 • 93.0 496 • 43.0 414 • 121.0 389 • 136.0 318 • 81.0 313

Compound Name	Score	Rev. Score	Prob. %	Library Name	CAS #	Library Id
3-Cyclohexene-1-methanol, α,α,4-trimethyl-, (R)-	866	893	16.23	mainlib	7785-53-7	32867

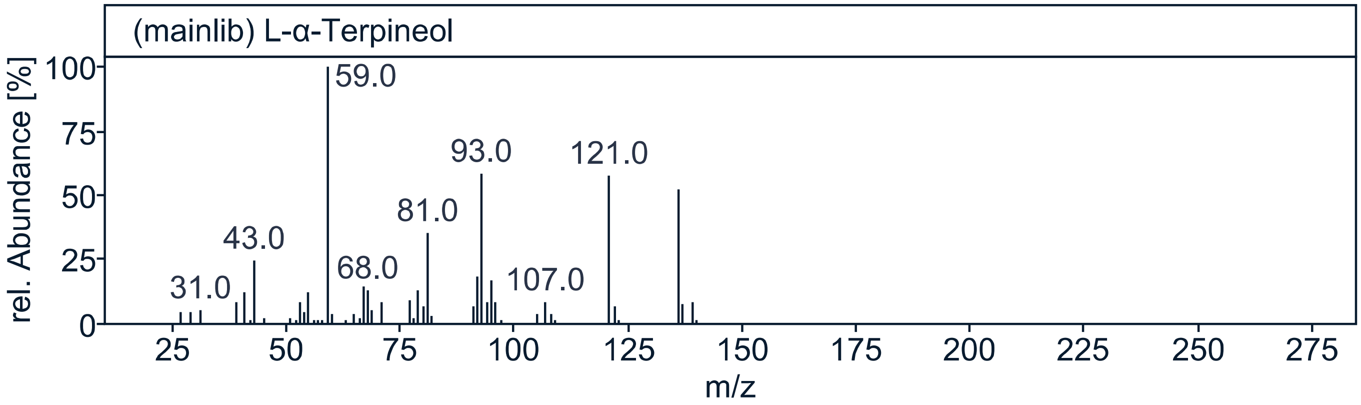

Ion Table

59.0 999 • 93.0 584 • 121.0 576 • 136.0 524 • 81.0 348 • 43.0 240

Compound Name	Score	Rev. Score	Prob. %	Library Name	CAS #	Library Id
L-α-Terpineol	854	868	10.81	mainlib	10482-56-1	32870

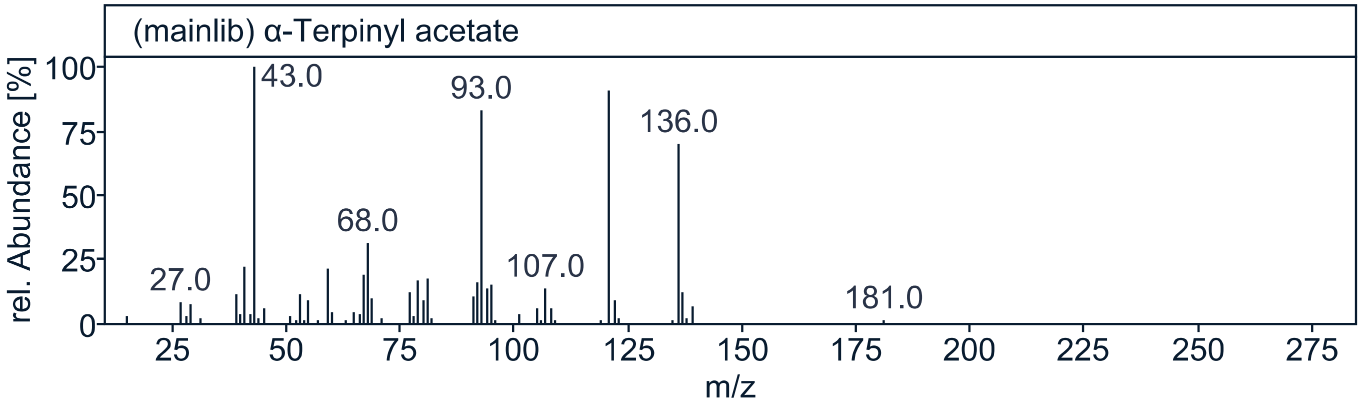

Ion Table

43.0 999 • 121.0 905 • 93.0 834 • 136.0 702 • 68.0 315 • 41.0 217

Compound Name	Score	Rev. Score	Prob. %	Library Name	CAS #	Library Id
α-Terpinyl acetate	843	881	7.41	mainlib	80-26-2	12465

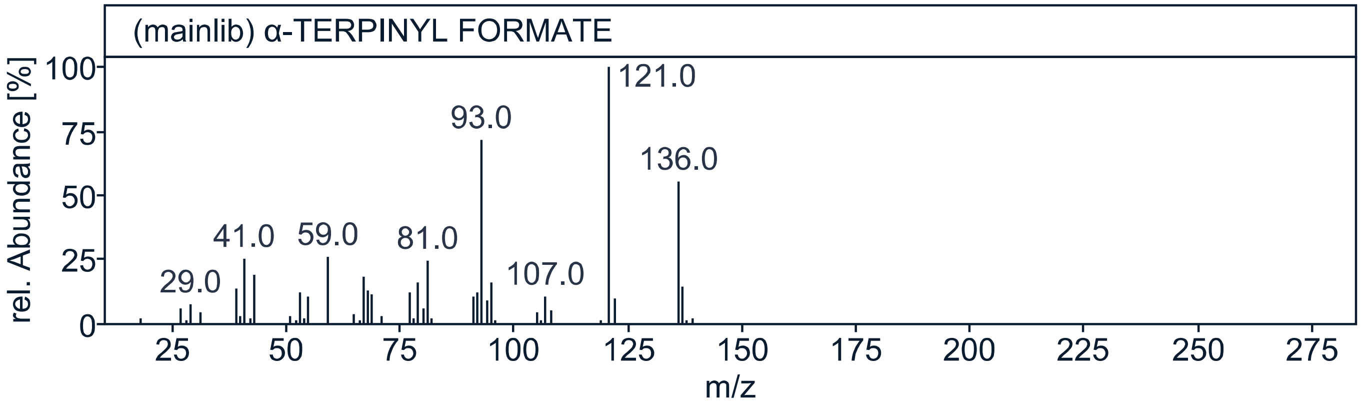

Ion Table

121.0 999 • 93.0 716 • 136.0 554 • 59.0 259 • 41.0 254 • 81.0 240

Compound Name	Score	Rev. Score	Prob. %	Library Name	CAS #	Library Id
α-TERPINYL FORMATE	819	875	2.48	mainlib		112330

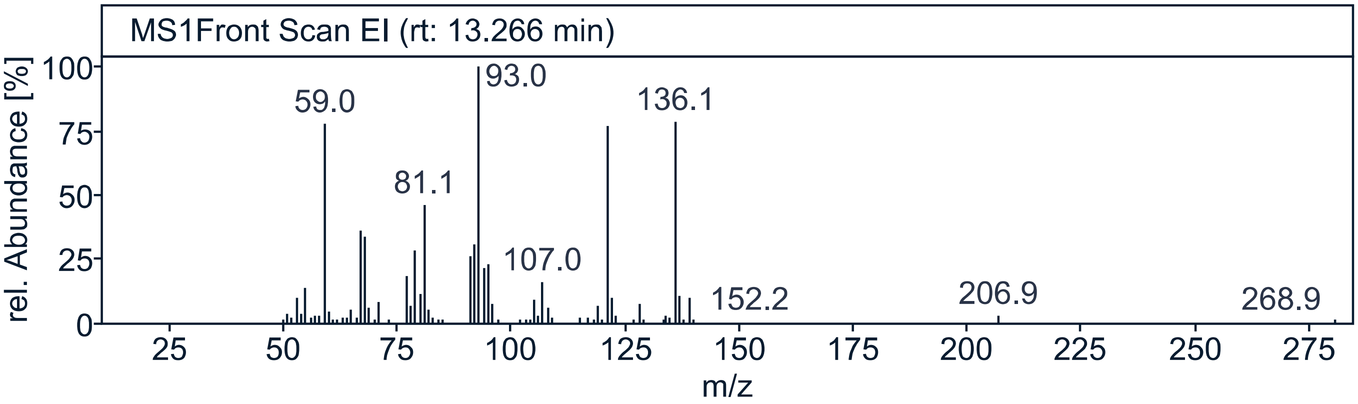

Ion Table

93.0 999 • 136.1 785 • 59.0 779 • 121.1 772 • 81.1 461 • 67.0 357

Summary Hit Table

Compound Name	Score	Rev. Score	Prob. %	Library Name	CAS #	Library Id
α-Terpineol	889	930	44.45	mainlib	98-55-5	32871
3-Cyclohexene-1-methanol, α,α,4-trimethyl-, (R)-	866	893	16.23	mainlib	7785-53-7	32867
L-α-Terpineol	854	868	10.81	mainlib	10482-56-1	32870
α-Terpinyl acetate	843	881	7.41	mainlib	80-26-2	12465
α-TERPINYL FORMATE	819	875	2.48	mainlib		112330

Peak @ 14.101 **Area** 11753721.898 **Area %** 19.02

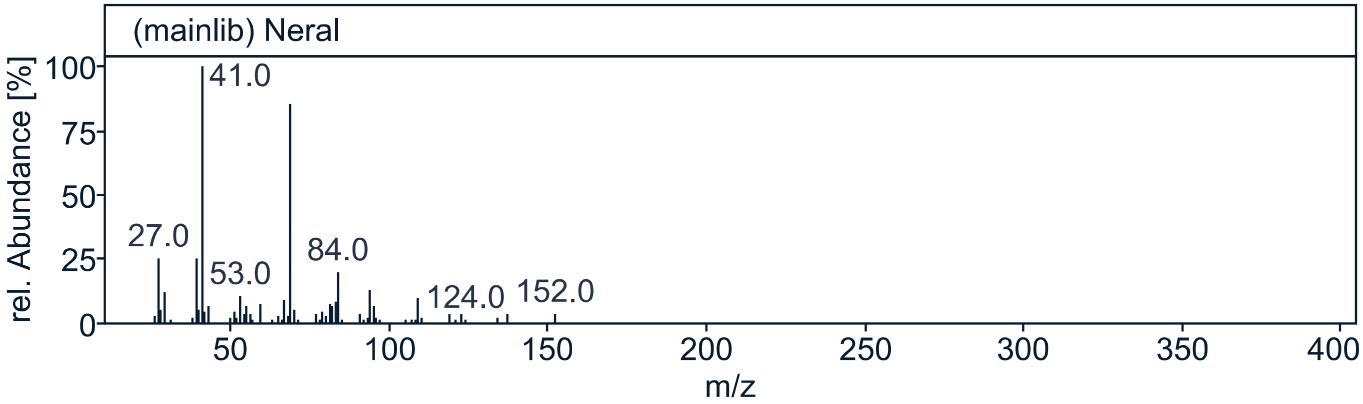

Ion Table

41.0 999 • 69.0 853 • 27.0 254 • 39.0 248 • 84.0 197 • 94.0 130

Compound Name	Score	Rev. Score	Prob. %	Library Name	CAS #	Library Id
Neral	892	897	30.44	mainlib	106-26-3	3557

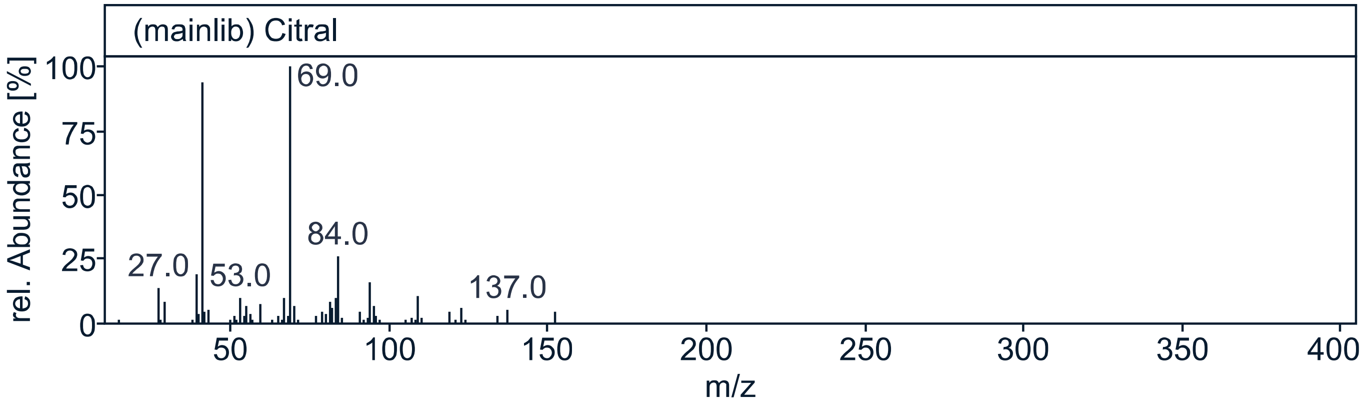

Ion Table

69.0 999 • 41.0 939 • 84.0 256 • 39.0 186 • 94.0 155 • 27.0 136

Compound Name	Score	Rev. Score	Prob. %	Library Name	CAS #	Library Id
Citral	888	889	25.71	mainlib	5392-40-5	37075

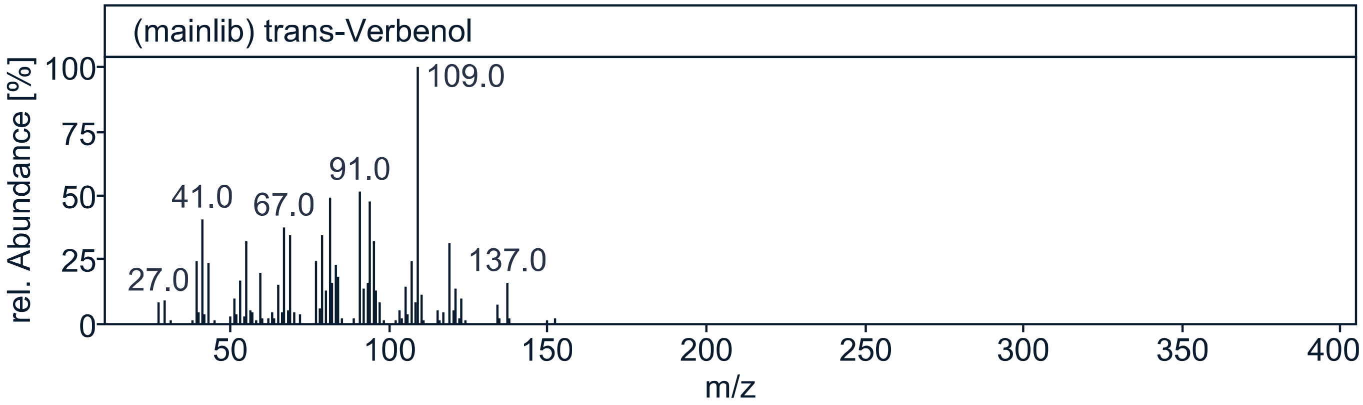

Ion Table

109.0 999 • 91.0 517 • 81.0 489 • 94.0 476 • 41.0 407 • 67.0 376

Compound Name	Score	Rev. Score	Prob. %	Library Name	CAS #	Library Id
trans-Verbenol	860	868	7.42	mainlib	1820-09-3	93946

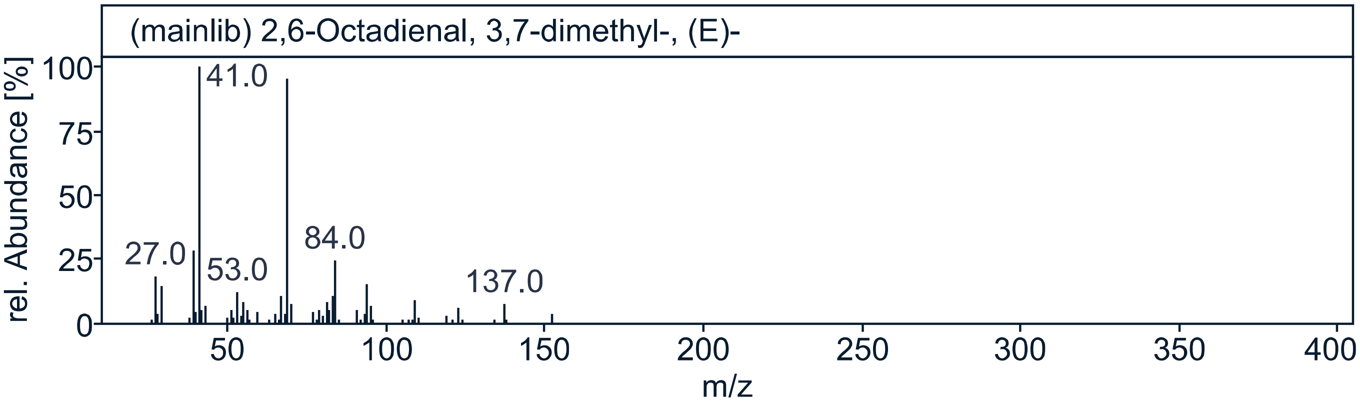

Ion Table

41.0 999 • 69.0 956 • 39.0 281 • 84.0 242 • 27.0 184 • 94.0 150

Compound Name	Score	Rev. Score	Prob. %	Library Name	CAS #	Library Id
2,6-Octadienal, 3,7-dimethyl-, (E)-	860	865	7.42	mainlib	141-27-5	3605

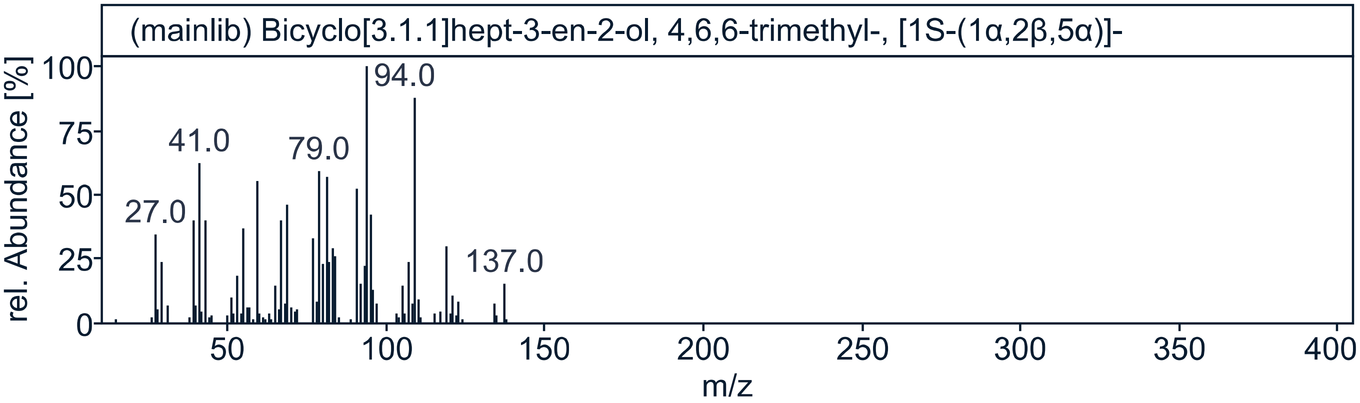

Ion Table

94.0 999 • 109.0 877 • 41.0 622 • 79.0 592 • 81.0 569 • 59.0 552

Compound Name	Score	Rev. Score	Prob. %	Library Name	CAS #	Library Id
Bicyclo[3.1.1]hept-3-en-2-ol, 4,6,6-trimethyl-, [1S-(1α,2β,	856	863	6.27	mainlib	18881-04-4	74073

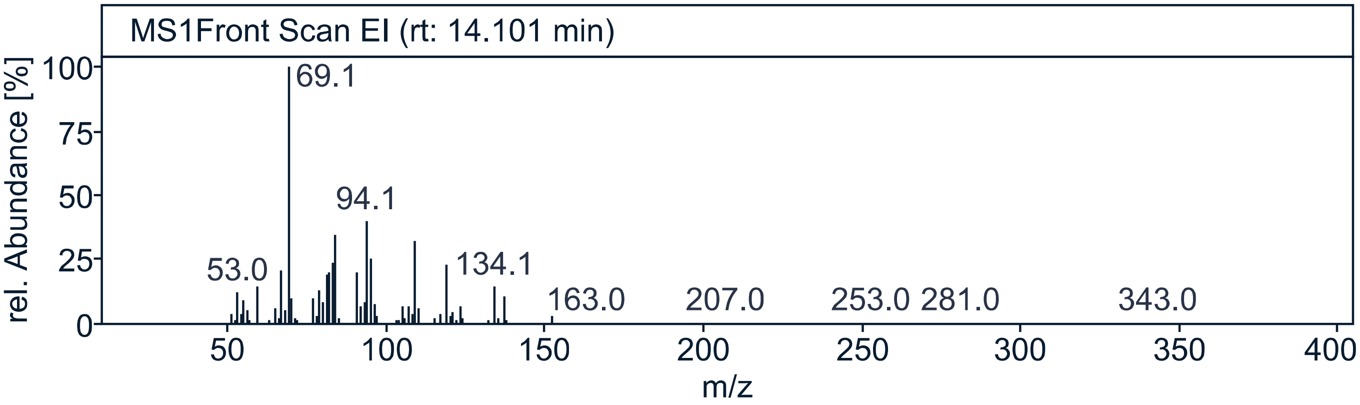

Ion Table

69.1 999 • 94.1 401 • 84.0 345 • 109.1 321 • 95.0 249 • 83.0 233

Summary Hit Table

Compound Name	Score	Rev. Score	Prob. %	Library Name	CAS #	Library Id
Neral	892	897	30.44	mainlib	106-26-3	3557
Citral	888	889	25.71	mainlib	5392-40-5	37075
trans-Verbenol	860	868	7.42	mainlib	1820-09-3	93946
2,6-Octadienal, 3,7-dimethyl-, (E)-	860	865	7.42	mainlib	141-27-5	3605
Bicyclo[3.1.1]hept-3-en-2-ol, 4,6,6-trimethyl-, [1S-(1α,2β,	856	863	6.27	mainlib	18881-04-4	74073

Peak @ 14.455 **Area** 17028120.384 **Area %** 27.55

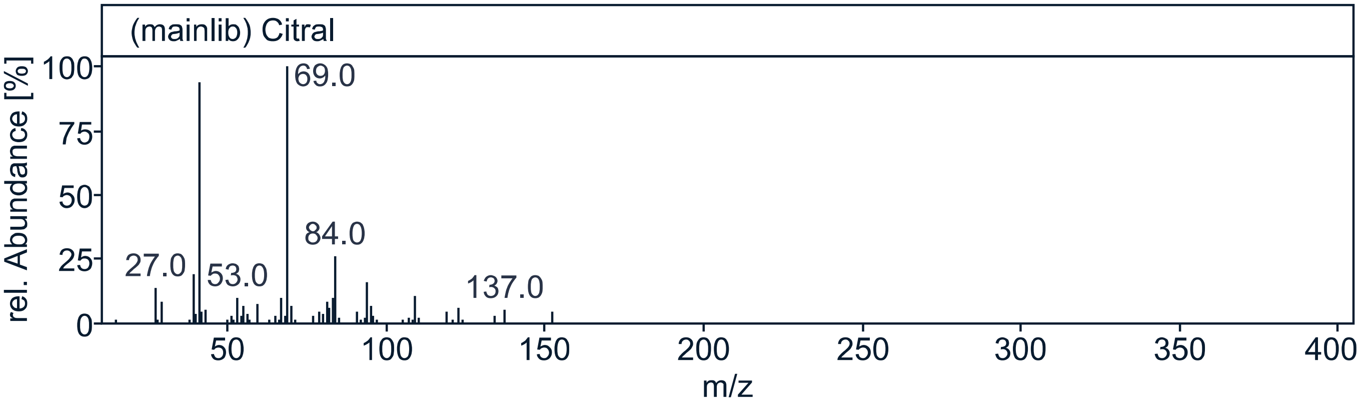

Ion Table

69.0 999 • 41.0 939 • 84.0 256 • 39.0 186 • 94.0 155 • 27.0 136

Compound Name	Score	Rev. Score	Prob. %	Library Name	CAS #	Library Id
Citral	938	938	45.26	mainlib	5392-40-5	37075

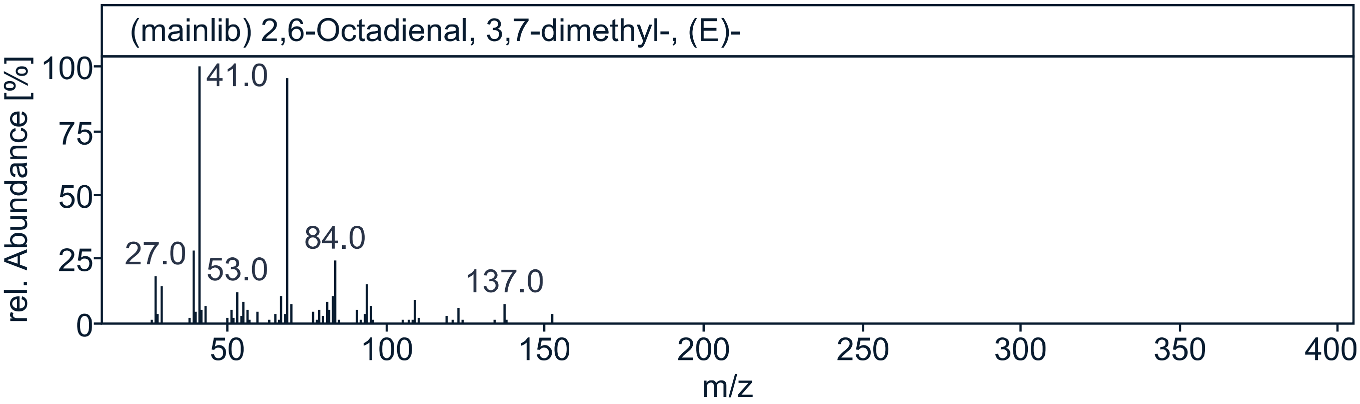

Ion Table

41.0 999 • 69.0 956 • 39.0 281 • 84.0 242 • 27.0 184 • 94.0 150

Compound Name	Score	Rev. Score	Prob. %	Library Name	CAS #	Library Id
2,6-Octadienal, 3,7-dimethyl-, (E)-	933	935	36.48	mainlib	141-27-5	3605

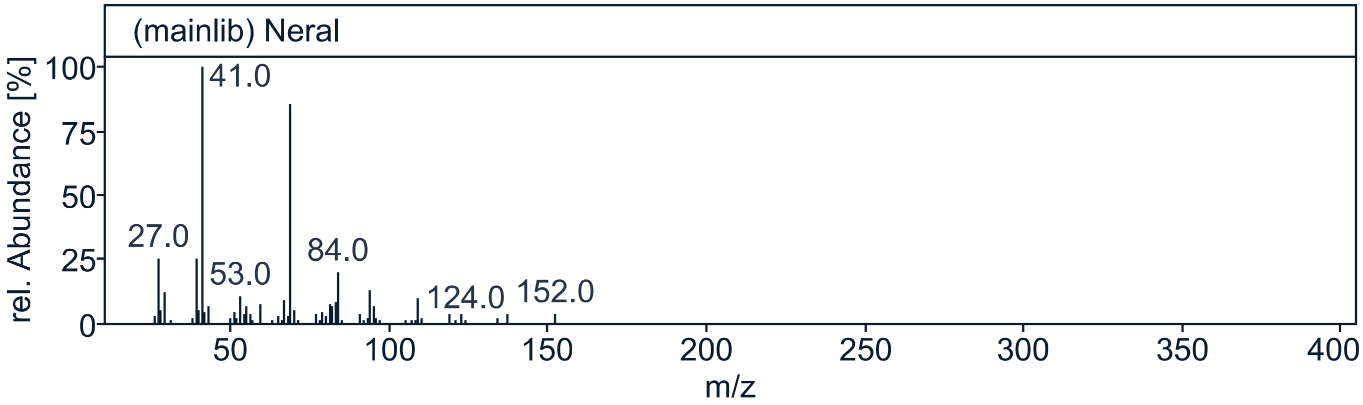

Ion Table

41.0 999 • 69.0 853 • 27.0 254 • 39.0 248 • 84.0 197 • 94.0 130

Compound Name	Score	Rev. Score	Prob. %	Library Name	CAS #	Library Id
Neral	911	912	14.42	mainlib	106-26-3	3557

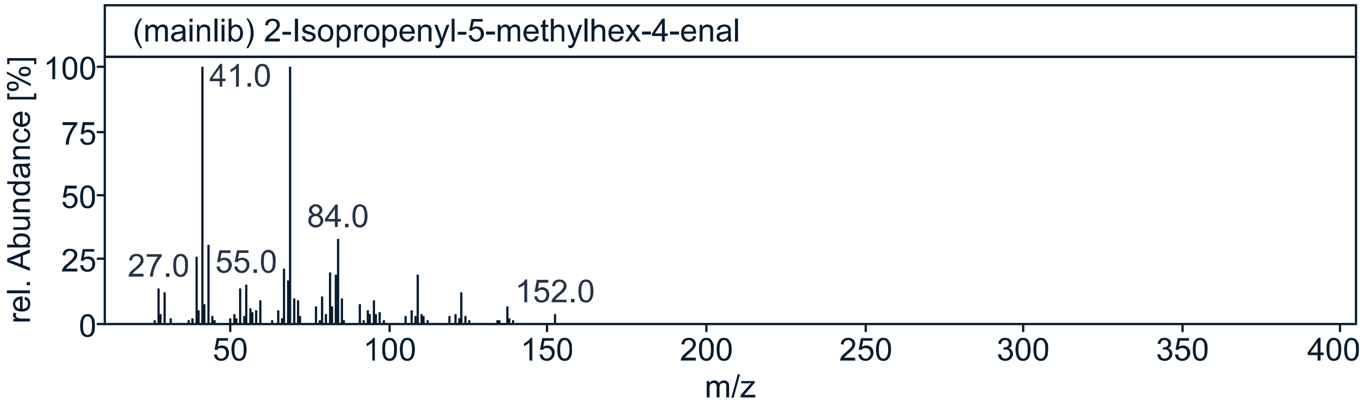

Ion Table

41.0 999 • 69.0 999 • 84.0 329 • 43.0 307 • 39.0 256 • 67.0 210

Compound Name	Score	Rev. Score	Prob. %	Library Name	CAS #	Library Id
2-Isopropenyl-5-methylhex-4-enal	773	778	0.51	mainlib	75697-98-2	3735

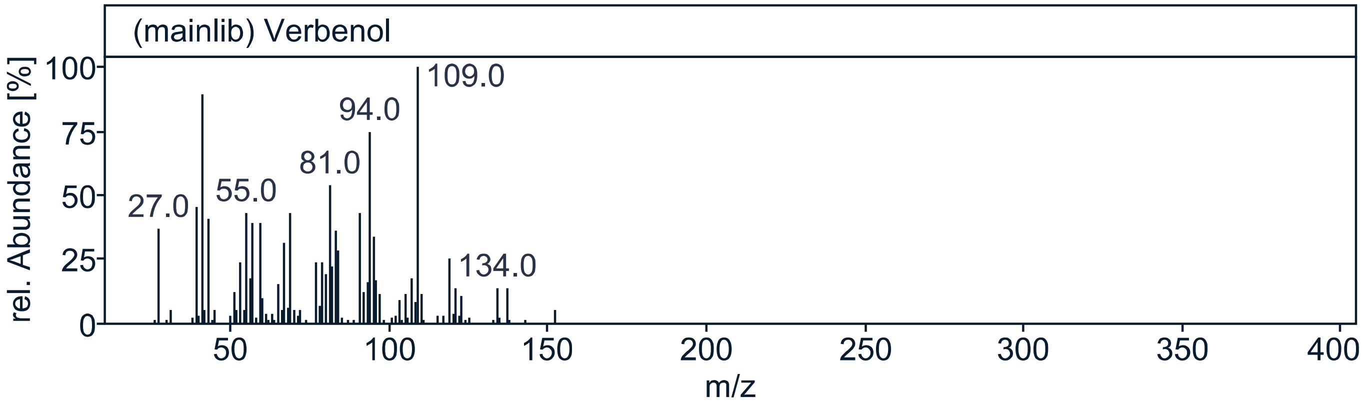

Ion Table

109.0 999 • 41.0 893 • 94.0 743 • 81.0 539 • 39.0 451 • 69.0 430

Compound Name	Score	Rev. Score	Prob. %	Library Name	CAS #	Library Id
Verbenol	763	766	0.36	mainlib	473-67-6	93487

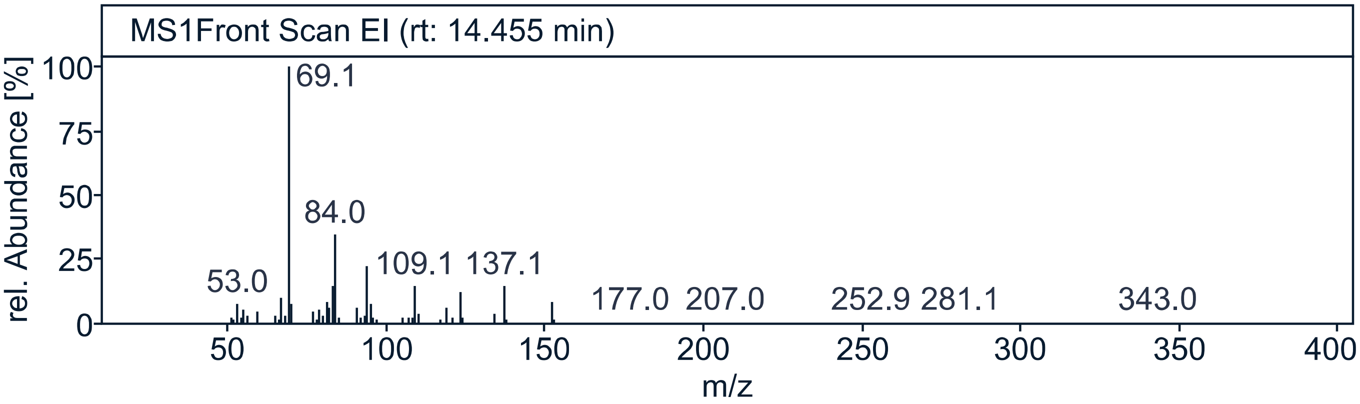

Ion Table

69.1 999 • 84.0 343 • 94.1 223 • 83.0 145 • 109.1 144 • 137.1 143

Summary Hit Table

Compound Name	Score	Rev. Score	Prob. %	Library Name	CAS #	Library Id
Citral	938	938	45.26	mainlib	5392-40-5	37075
2,6-Octadienal, 3,7-dimethyl-, (E)-	933	935	36.48	mainlib	141-27-5	3605
Neral	911	912	14.42	mainlib	106-26-3	3557
2-Isopropenyl-5-methylhex-4-enal	773	778	0.51	mainlib	75697-98-2	3735
Verbenol	763	766	0.36	mainlib	473-67-6	93487

Peak @ 15.580 **Area** 11398865.456 **Area %** 18.44

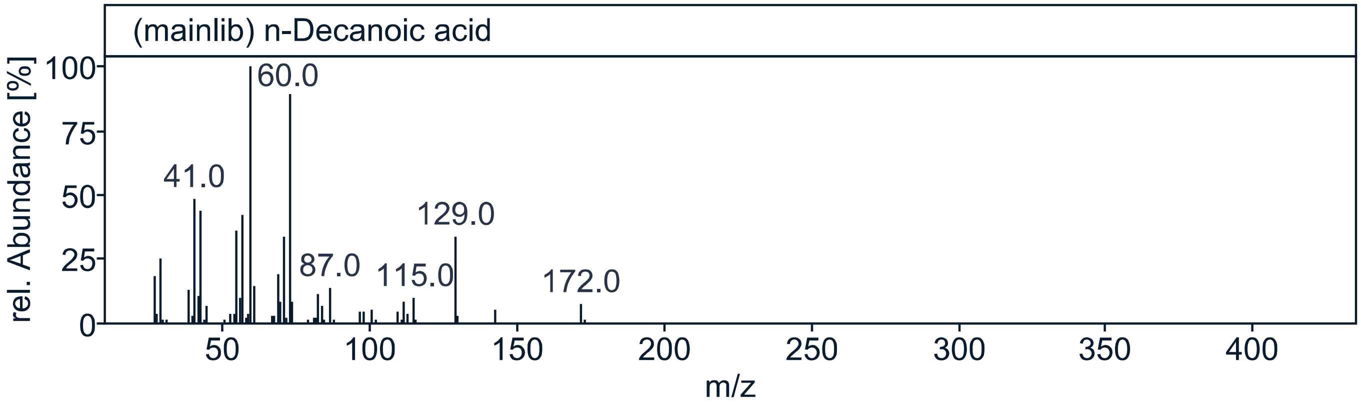

Ion Table

60.0 999 • 73.0 891 • 41.0 484 • 43.0 435 • 57.0 420 • 55.0 357

Compound Name	Score	Rev. Score	Prob. %	Library Name	CAS #	Library Id
n-Decanoic acid	898	916	85.25	mainlib	334-48-5	33538

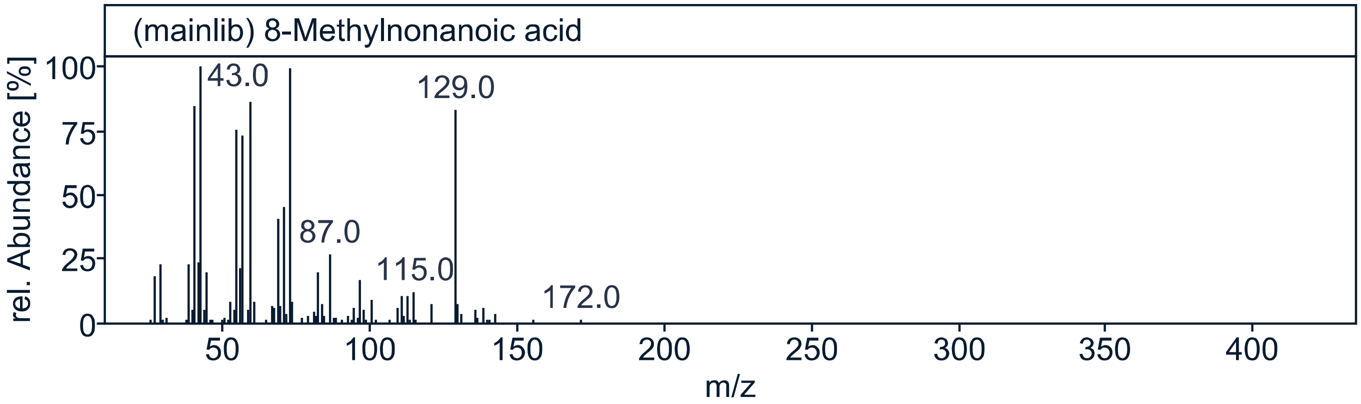

Ion Table

43.0 999 • 73.0 990 • 60.0 862 • 41.0 849 • 129.0 835 • 55.0 755

Compound Name	Score	Rev. Score	Prob. %	Library Name	CAS #	Library Id
8-Methylnonanoic acid	802	831	6.79	mainlib	5963-14-4	9949

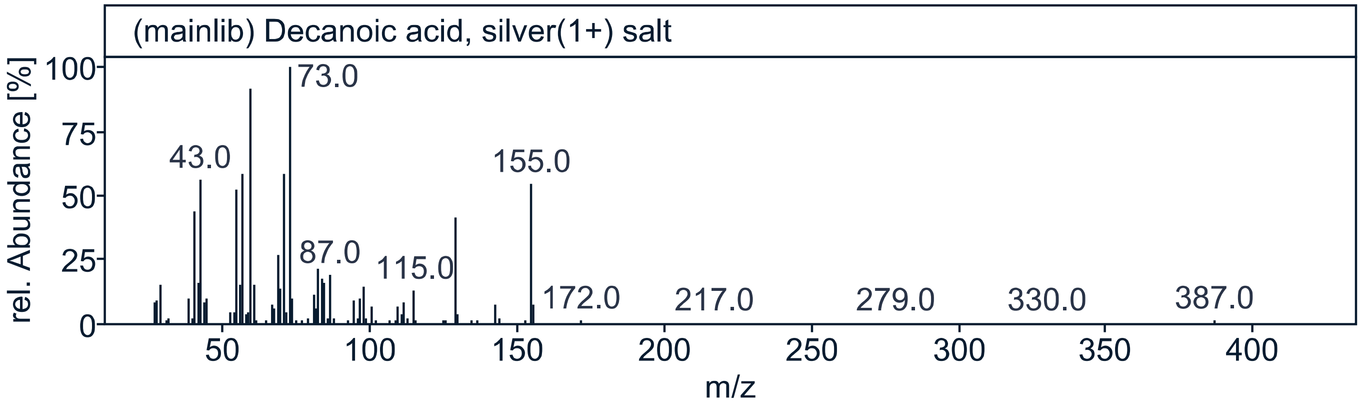

Ion Table

73.0 999 • 60.0 920 • 57.0 586 • 71.0 580 • 43.0 563 • 155.0 547

Compound Name	Score	Rev. Score	Prob. %	Library Name	CAS #	Library Id
Decanoic acid, silver(1+) salt	774	799	1.96	mainlib	13126-67-5	44901

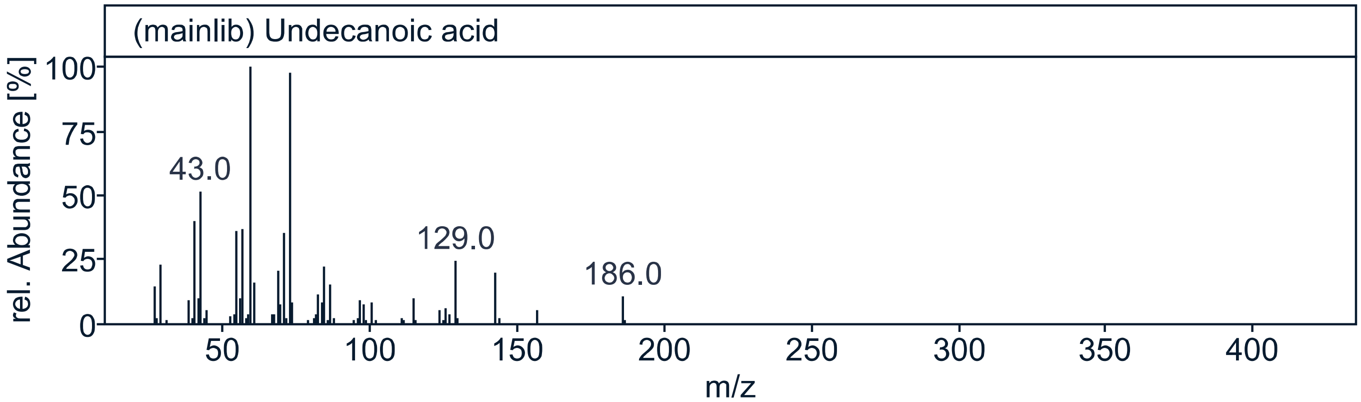

Ion Table

60.0 999 • 73.0 975 • 43.0 517 • 41.0 398 • 57.0 364 • 55.0 357

Compound Name	Score	Rev. Score	Prob. %	Library Name	CAS #	Library Id
Undecanoic acid	773	797	1.88	mainlib	112-37-8	33547

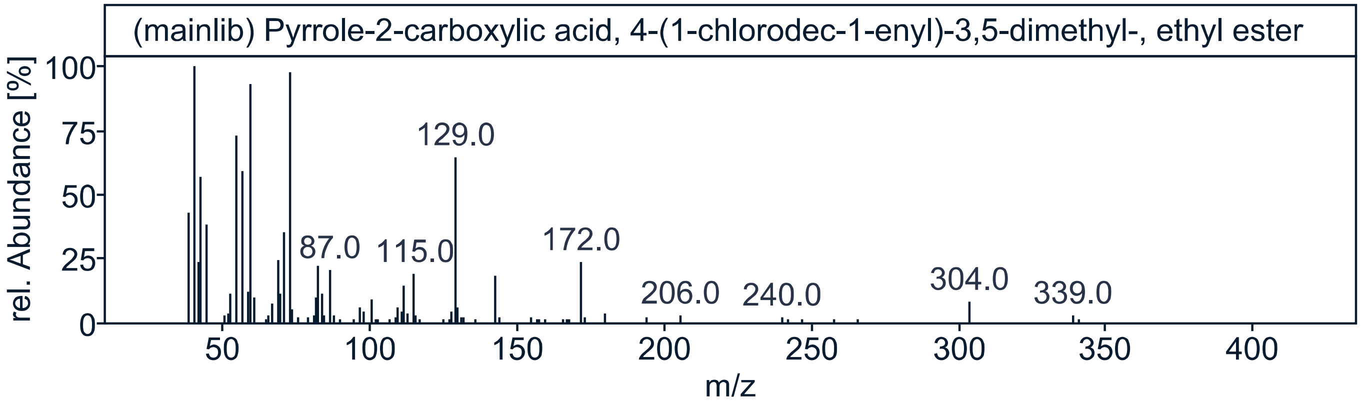

Ion Table

41.0 999 • 73.0 979 • 60.0 932 • 55.0 734 • 129.0 647 • 57.0 593

Compound Name	Score	Rev. Score	Prob. %	Library Name	CAS #	Library Id
Pyrrole-2-carboxylic acid, 4-(1-chlorodec-1-enyl)-3,5-dimet	763	793	1.33	mainlib		3871

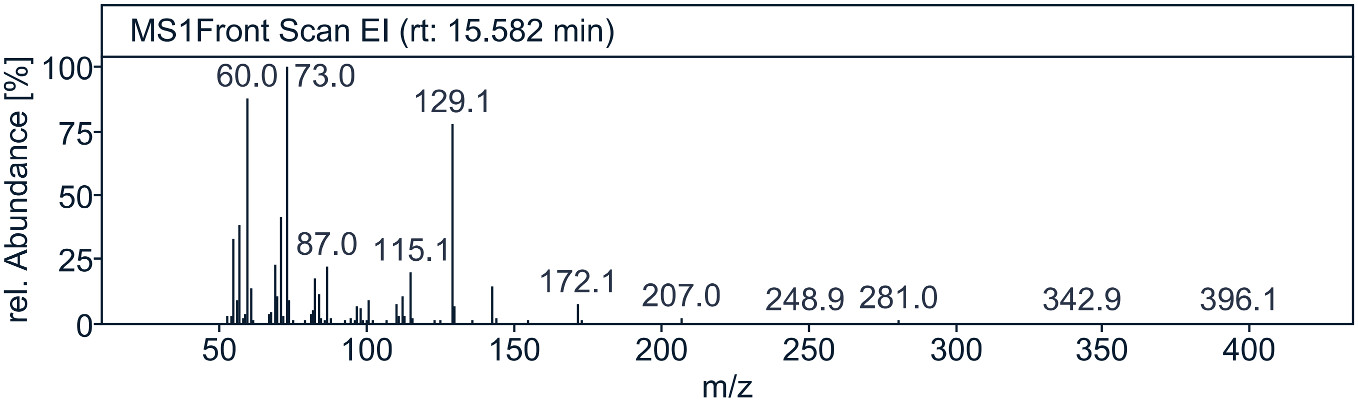

Ion Table

73.0 999 • 60.0 882 • 129.1 775 • 71.1 412 • 57.0 383 • 55.0 327

Summary Hit Table

Compound Name	Score	Rev. Score	Prob. %	Library Name	CAS #	Library Id
n-Decanoic acid	898	916	85.25	mainlib	334-48-5	33538
8-Methylnonanoic acid	802	831	6.79	mainlib	5963-14-4	9949
Decanoic acid, silver(1+) salt	774	799	1.96	mainlib	13126-67-5	44901
Undecanoic acid	773	797	1.88	mainlib	112-37-8	33547
Pyrrole-2-carboxylic acid, 4-(1-chlorodec-1-enyl)-3,5-dimet	763	793	1.33	mainlib		3871

**Peak @**	15.878	**Area**	1225380.586	**Area %**	1.98

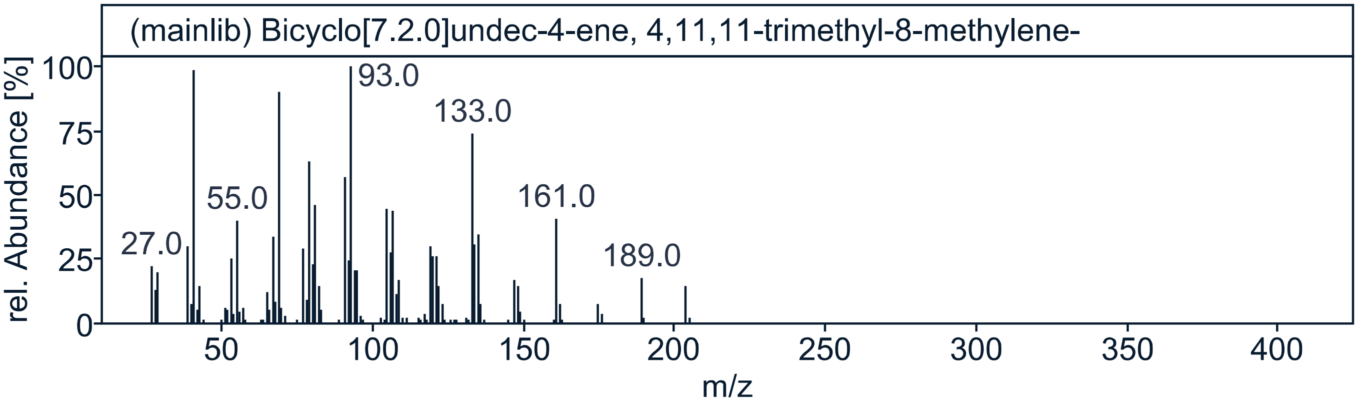

Ion Table

93.0 999 • 41.0 988 • 69.0 902 • 133.0 735 • 79.0 631 • 91.0 567

**Compound Name**	**Score**	**Rev. Score**	**Prob. %**	**Library Name**	**CAS #**	**Library Id**
Bicyclo[7.2.0]undec-4-ene, 4,11,11-trimethyl-8-methylene-	794	897	8.37	mainlib	13877-93-5	72373

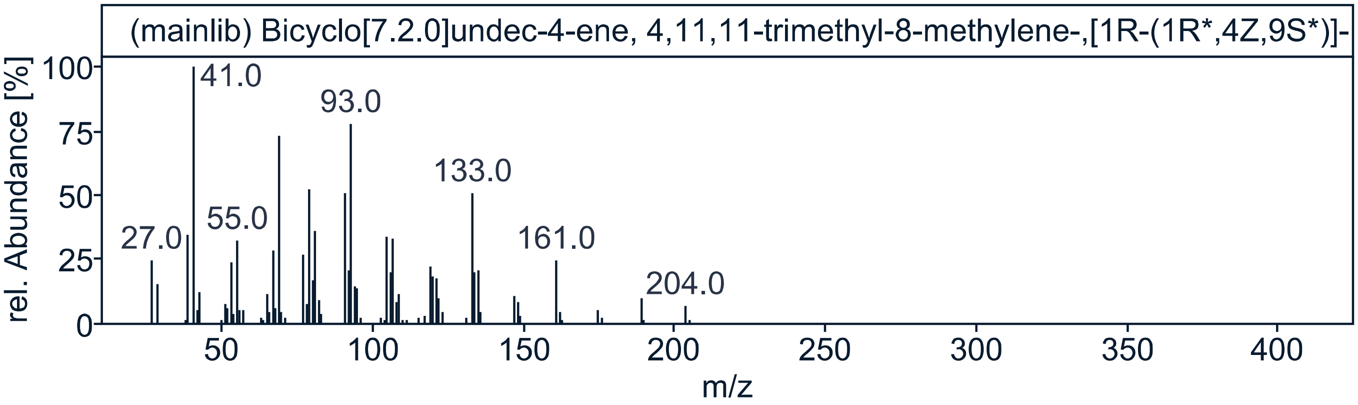

Ion Table

41.0 999 • 93.0 779 • 69.0 730 • 79.0 519 • 91.0 506 • 133.0 505

**Compound Name**	**Score**	**Rev. Score**	**Prob. %**	**Library Name**	**CAS #**	**Library Id**
Bicyclo[7.2.0]undec-4-ene, 4,11,11-trimethyl-8-methylene-,[	784	885	5.91	mainlib	118-65-0	4101

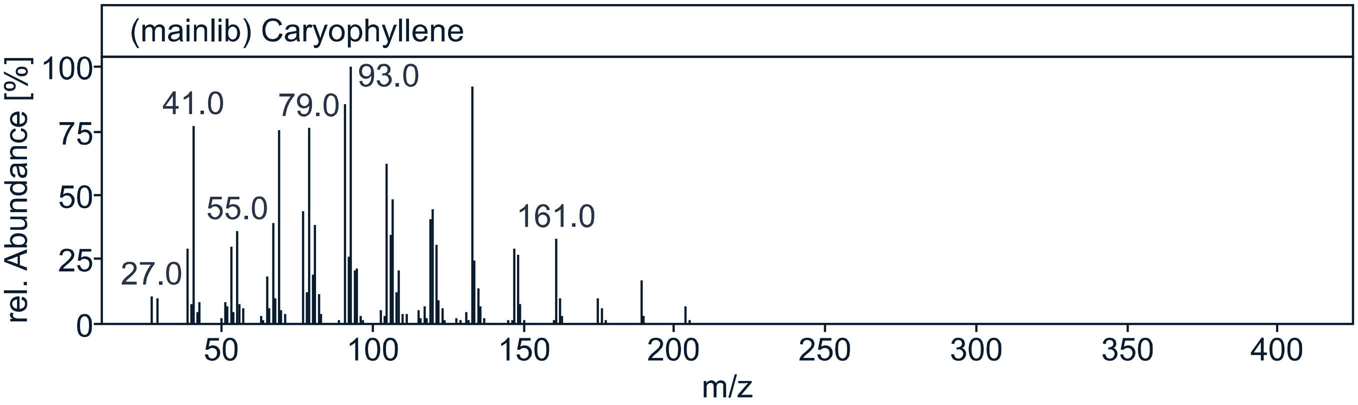

Ion Table

93.0 999 • 133.0 921 • 91.0 858 • 41.0 769 • 79.0 763 • 69.0 754

Compound Name	Score	Rev. Score	Prob. %	Library Name	CAS #	Library Id
Caryophyllene	783	872	5.68	mainlib	87-44-5	73222

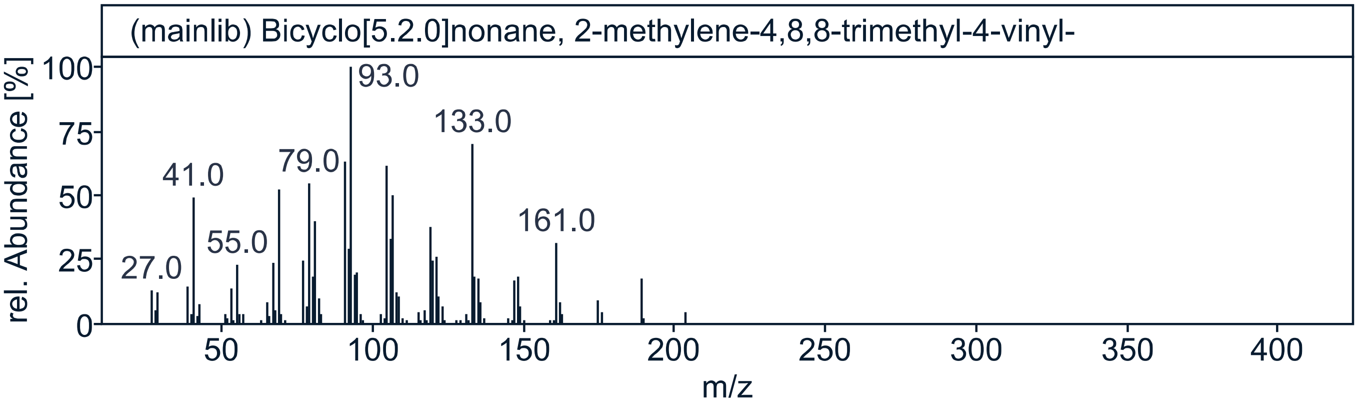

Ion Table

93.0 999 • 133.0 702 • 91.0 631 • 105.0 616 • 79.0 548 • 69.0 519

Compound Name	Score	Rev. Score	Prob. %	Library Name	CAS #	Library Id
Bicyclo[5.2.0]nonane, 2-methylene-4,8,8-trimethyl-4-vinyl-	778	865	4.58	mainlib	242794-76-9	73221

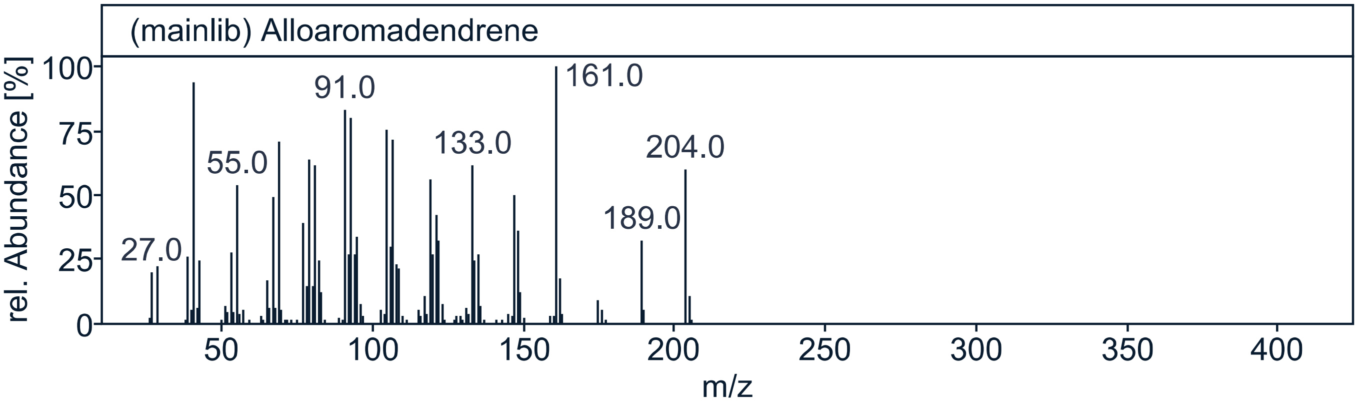

Ion Table

161.0 999 • 41.0 940 • 91.0 835 • 93.0 800 • 105.0 752 • 107.0 716

Compound Name	Score	Rev. Score	Prob. %	Library Name	CAS #	Library Id
Alloaromadendrene	771	862	3.5	mainlib	25246-27-9	164982

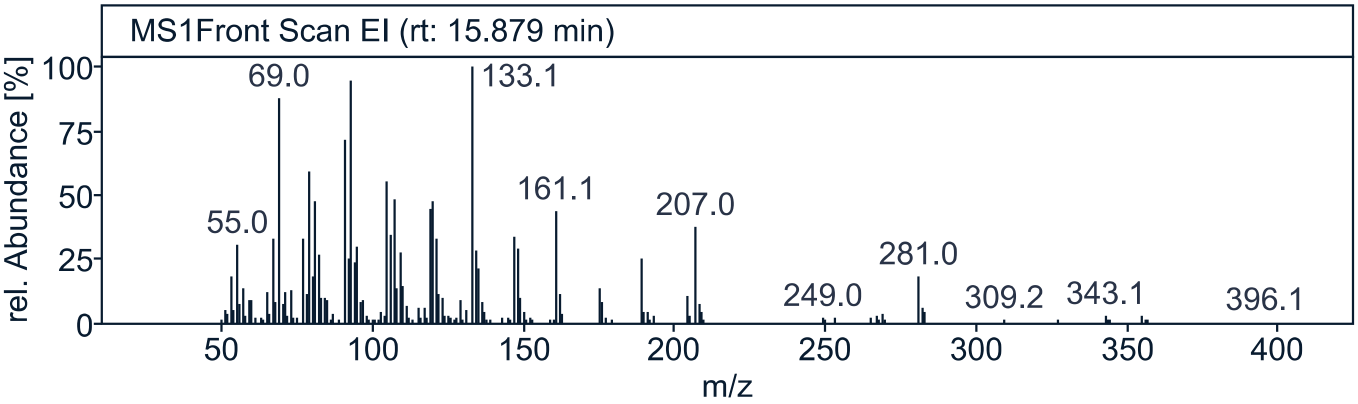

Ion Table

133.1 999 • 93.1 950 • 69.0 877 • 91.0 716 • 79.0 591 • 105.0 550

Summary Hit Table

Compound Name	Score	Rev. Score	Prob. %	Library Name	CAS #	Library Id
Bicyclo[7.2.0]undec-4-ene, 4,11,11-trimethyl-8-methylene-	794	897	8.37	mainlib	13877-93-5	72373
Bicyclo[7.2.0]undec-4-ene, 4,11,11-trimethyl-8-methylene-,[	784	885	5.91	mainlib	118-65-0	4101
Caryophyllene	783	872	5.68	mainlib	87-44-5	73222
Bicyclo[5.2.0]nonane, 2-methylene-4,8,8-trimethyl-4-vinyl-	778	865	4.58	mainlib	242794-76-9	73221
Alloaromadendrene	771	862	3.5	mainlib	25246-27-9	164982
